# Investigation of Three-Dimensional Microstructure of Tricalcium Silicate (C_3_S) by Electron Microscopy

**DOI:** 10.3390/ma11071110

**Published:** 2018-06-29

**Authors:** Fei Yang, Xianping Liu, Yongjuan Zhao, Yongming Zhang, Peiming Wang, Ian Robinson, Bo Chen

**Affiliations:** 1 School of Materials Science and Engineering, Tongji University, Shanghai 201804, China; 1610413@tongji.edu.cn (F.Y.); 1730602@tongji.edu.cn (Y.Zhao.); zym126@tongji.edu.cn (Y.Zhang.); tjwpm@126.com (P.W.); i.robinson@ucl.ac.uk (I.R.); 2 Key Laboratory of Advanced Civil Engineering Materials (Tongji University), Ministry of Education, Shanghai 201804, China; 3 London Centre for Nanotechnology, University College London, London WC1H 0AH, UK; 4 Division of Condensed Matter Physics and Materials Science, Brookhaven National Laboratory, Upton, NY 11973, USA

**Keywords:** serial block-face scanning electron microscopy (SBFSEM), three-dimensional (3D) structure, porosity, tricalcium silicate (C_3_S)

## Abstract

A serial block-face scanning electron microscopy (SBFSEM) system, composed of a scanning electron microscope (SEM) and an ultra-microtome installed within the SEM vacuum chamber, has been used to characterize the three-dimensional (3D) microstructure of tricalcium silicate (C_3_S) grains embedded in epoxy resin. A selection of C_3_S grains were segmented and rendered with 3D-image processing software, which allowed the C_3_S grains to be clearly visualized and enabled statistically quantitative analysis. The results show that about 5% of the C_3_S grains have volumes larger than 1 μm^3^ and the average volume of the grains is 25 μm^3^. Pores can also be clearly seen in the biggest C_3_S grain, the volume of which is 3.6 × 10^4^ μm^3^, and the mean volume and total volume of all the pores within this grain are 4.8 μm^3^ and 3.0 × 10^3^ μm^3^, respectively. The reported work provides a new approach for the characterization of the 3D spatial structure of raw C_3_S materials, and the resulting 3D structure of the raw C_3_S is important for further systematic research on the relationships between the spatial microstructure and the hydration kinetics of C_3_S and other cement minerals.

## 1. Introduction

As it is well known, tricalcium silicate (3CaO·SiO_2_, or simplified as C_3_S) is the main component of ordinary Portland cement, and it plays an important role in building the strength of the cement paste during its hydration [1,2]. What is more, the C_3_S mineral phase controls the normal setting rate and the early-stage strength development of Portland cement paste, mortar and concrete [3]. The hydration process of cement is influenced by various factors and is very complicated. Nevertheless, the morphology of C_3_S grains within the materials has a direct effect on the hydration capacity of cement, the amount of required cement in the cement-based materials, as well as the eventual properties and performance of the cement paste and concrete. For these reasons, researchers often treat the monophase C_3_S mineral as a simplified cement for study [4,5,6,7], and so the properties and hydration behavior of C_3_S have been extensively investigated [8,9,10,11]. Up to now, the two-dimensional microstructure of cement particles has been extensively studied to correlate their morphologies with their properties [12,13,14,15,16,17,18,19,20]. However, the reported work extends such study to three dimensions, where the capabilities to monitor the grain porosity and volume-related properties have significantly improved. The properties of C_3_S or cement paste are heavily influenced by the morphology and size distribution of the (raw) particles within them [21]. As Xi [22] pointed out, pore structure is a vital component of the microstructure of cement-based materials, which directly affects the macroscopic properties such as the strength, durability, elastic modulus, corrosion resistance, shrinkage, and permeability of the concrete.

In this work, the three-dimensional (3D) structure of the raw C_3_S grains, including the pore structure within the grains, was observed using serial block-face scanning electron microscopy (SBFSEM) [23,24,25], which can reveal the 3D microstructure of the measured objects with a spatial resolution of up to 2–3 nm in the XY planes (imaging planes) and of about 15 nm along the Z-axis (slicing direction). SBFSEM has been proven to be a very powerful tool for the investigation of the internal structure of biological specimens, with a considerable amount of literature having been reported [26,27,28,29]. This is because both the sample preparation and imaging methods are well-matched with soft-condensed matters and materials, for which the SBFSEM was initially developed. Meanwhile, the investigation of hard-condensed materials using SBFSEM is in progress [30,31]. However, there is still no report on studying the cement components such as C_3_S, tricalcium aluminate(C_3_A), dicalcium silicate(C_2_S) or cement-based materials by SBFSEM. 

In the reported work, SBFSEM, a 3D-structure investigation technique normally used for biological materials, was extended further to be applied to cement-based materials that are represented by the C_3_S grains here. This work demonstrates a new approach for studying the monocrystalline component of cement and the results can be used in future studies on the mechanism of C_3_S hydration.

## 2. Materials and Methods

**Figure 1:**
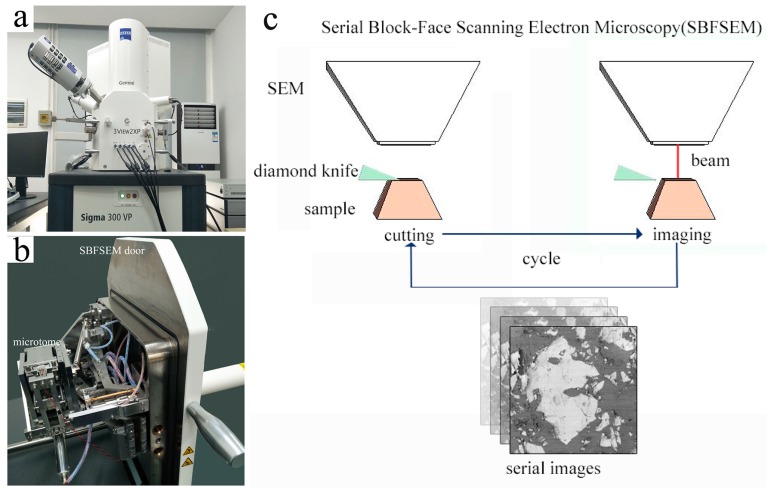
Serial block-face scanning electron microscopy (SBFSEM) system and its workflow: (**a**) The SBFSEM instrument; (**b**) Ultra-microtome attached to the inner side of the SEM vacuum chamber door; (**c**) Schematic diagram showing the workflow of acquiring serial image slices by using SBFSEM.

The SBFSEM system (Figure 1) mainly consisted of a scanning electron microscope (SEM, Zeiss Sigma 300 VP, Carl Zeiss Microscopy GmbH, Oberkochen, Germany), an ultra-microtome with a diamond knife (Gatan 3View2XP, Gatan UK, Abingdon, UK) installed within the vacuum chamber of the microscope (see Figure 1b), and the controlling hardware and software to manage the serial sectioning and imaging processes. For the measurements, the well-shaped, pre-prepared sample was firstly inserted into the SBFSEM sample holder in the ultra-microtome system, and then a manual approach procedure was carried out under the observation of an optical microscope to move the sample surface up to until it could be just cut by the diamond knife. The SEM chamber was then closed and pumped down, the imaging parameters were set, and the automatic acquisition was started. Serial imaging of the samples was conducted by using a highly efficient backscattered electron (BSE) detector. Each time, once the region of interest on the freshly cut sample surface had been imaged, the sample was moved up by the nominal slice thickness (usually in the range of 15–100 nm) and then the diamond knife cut the entire surface of the sample block again. After the cutting, the area of interest was imaged on the freshly exposed surface again. These sectioning-and-imaging cycles run consecutively and can acquire a series of thousands of BSE micrography slices without user interaction, if desired. A schematic diagram of the workflow of acquiring serial image slices by using SBFSEM is shown in Figure 1c. The sequence of the sectioning and imaging was automated, and hence has the additional advantage that all the images were correctly aligned and almost free of distortion [32,33].

C_3_S was synthesized according to the procedures used by De la Torre et al. [34]. All the raw materials including CaCO_3_, Mg(OH)_2.4_MgCO_3.5_H_2_O, SiO_2_, and Al_2_O_3_ were from Sinopharm Chemical Reagent Co. Ltd., Shanghai, China. They were weighed according to the stoichiometric ratio of Ca_2.92_Mg_0.06_O_3_(SiO_2_)_0.98_(Al_2_O_3_)_0.02_. The raw materials with this ratio were then mixed and placed in a Pt crucible, then preheated in a furnace at 1000 °C for 6 h. Subsequently, the preheated materials were ground into granules in an agate mortar, poured into a metal grinder, and ground again. Then, they were pressed into a cylinder by a compressor, heated in a furnace at 1450 °C for 12 h, and then cooled. The obtained materials were crushed, grinded, pressed and heated again in the furnace at 1500 °C for another 6 h and then cooled. The above procedures were repeated until the content of C_3_S in the materials reached more than 98% (measured by powder X-ray diffraction).

The synthesized C_3_S was then ground into powder, embedded and fixed in an epoxy resin for SBFSEM measurements. A multiple component Agar 100 resin was selected to embed the C_3_S grains following the technical procedure offered by Agar Scientific Ltd. [35]. Subsequently, the top of the C_3_S embedded sample was trimmed to a pyramid shape using a Leica EM UC7 microtome (Leica Microsystems Inc., Buffalo, IL, USA) with a glass knife. The trimmed sample was then super-glued onto the top of a small rivet-shaped Al sample mount and placed and fixed into a removable sample holder with a diameter of 1 cm, which was then installed in the sample stage of the ultra-microtome system of SBFSEM. In order to reduce the charging effect from the sample, the side surface and the bottom of the sample were covered by conductive silver paint to connect the sample with the body of the Al sample mount to form an electrically conductive bridge between the sample stage and the sample surface.

The C_3_S embedded sample was then measured by SBFSEM, and the instrument was operated in high vacuum (<3 × 10^−4^ Pa) at an acceleration voltage of 1 kV with a beam current of 200 nA. The cutting speed of the diamond knife was set at 0.5 mm/s. The BSE detector collected signals with a dwell time of 2 µs on each pixel and generated BSE micrographs with a pixel size of 49 × 49 nm. In total, 200 slices of the sample, of 50-nm thickness with a field of view of 100 × 100 µm, were acquired after about 2 h of sequential measurement.

## 3. Results and Discussions

**Figure 2:**
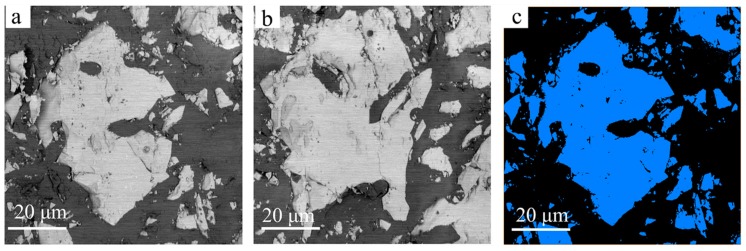
Two-dimensional images of the embedded tricalcium silicate (C_3_S) grains from SBFSEM: (**a**) The first backscattered electron (BSE) micrography slice of the C_3_S grains acquired by SBFSEM; (**b**) The last BSE micrography slice of the C_3_S grains; (**c**) Binary image of the first slice of the stack of slice images as shown in panel (**a**).

The first and last two-dimensional (2D) BSE micrography slices out of the series of 200 slices of the embedded C_3_S sample are shown as Figure 2a,b with an interval distance of 10 µm. Since the BSE signals scale with the atomic masses and electron densities within the sample, the brighter features within the BSE micrographs were identified as C_3_S grains, the darker regions among different brighter grains as epoxy resin, and the remaining darker regions within the grains as pores. The distribution of C_3_S grains and pores within them can be clearly seen in these images. The first step of analyzing the 3D image data is to binarize these micrography slices of the embedded C_3_S sample, and one of the resulting images is shown in Figure 2c, which is the first slice of the stack of slice images as shown in Figure 2a. For the binary image (Figure 2c), the blue parts represent C_3_S grains obtained through noise reduction and threshold segmentation by Avizo. It is an advanced 3D-image analysis software that provides optimized workflows for image processing and materials property simulations.

**Figure 3:**
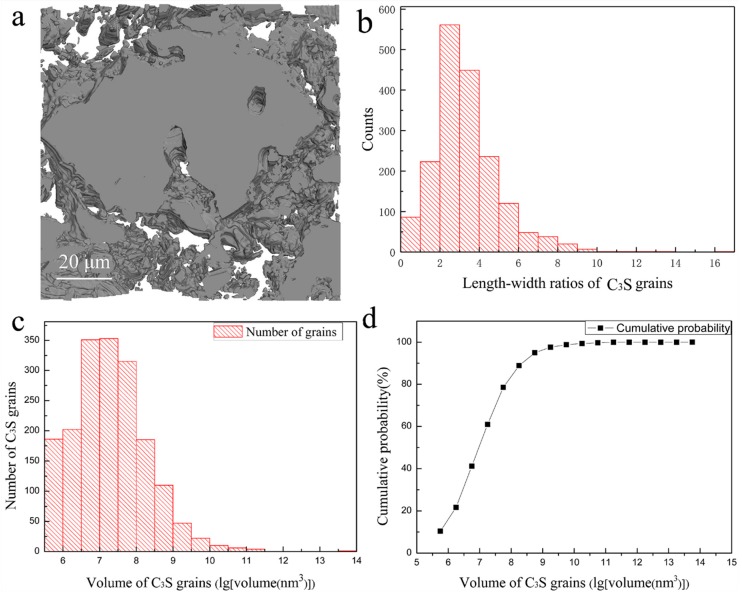
3D rendering and characterization of the C_3_S grains: (**a**) 3D Rendering of the acquired volume containing C_3_S grains; (**b**) A histogram (with step of 1) of the length–width ratios of the measured C_3_S grains; (**c**) A histogram of the volume distribution (with 0.5 step in logarithm coordinate) of the C_3_S grains; (**d**) The curve of volumetric cumulative probability of the C_3_S grains.

The obtained serial slice images were then stacked together by Avizo to generate the 3D image for quantitative analysis. Noise reduction, image registration, gray-scale based segmentation, volume fraction quantification and 3D rendering of the structure were performed by Avizo [36], and Figure 3a shows the 3D image obtained according to the above procedure, in which the gray parts are C_3_S grains. It clearly reveals the spatial distribution of the C_3_S grains fixed within the epoxy resin and the pores within the grains. From Figure 2 and Figure 3a, it can be seen that the outer shape of the C_3_S grains is irregular and the grain sizes are not uniform. The length–width ratios (LWRs) of the C_3_S grains are presented in Figure 3b, which shows that most of the LWR values are between 3 and 5. This means that most of the particles are quasi-acicular shaped rather than spherical shaped. This would lead the actually required quantity of water, i.e., the water–cement ratio, to be higher than the theoretically required quantity for the full hydration. This can have a significant impact on the physical and hydration properties of the C_3_S grains themselves. The LWR is defined as the ratio of the maximum Feret diameters against its minimum ones [37]. All the data extracted from a total of 1792 grains are listed in Supplementary Spreadsheet S1. From the spreadsheet and Figure 3c,d, it is clear that the volumes of the C_3_S grains range from 4.8 × 10^−4^ μm^3^ to 3.6 × 10^4^ μm^3^.These C_3_S grains have a relatively wide volume distribution, while about 95% of the grains are less than 1 μm^3^ big and the smallest volume that can be measured by SBFSEM is 4.8 × 10^−4^ μm^3^. Based on the segmentation of the 3D images, the statistical mean values of length, width, and volume of all the measured C_3_S grains are 1174 nm, 332 nm, and 25 μm^3^, respectively. The largest grain in the measured volume is 3.6 × 10^4^ μm^3^, which is crossing the whole observed volume. This means that it is not fully visualized by the SBFSEM measurement, i.e., the real volume of this largest grain is actually larger than 3.6 × 10^4^ μm^3^.

**Figure 4:**
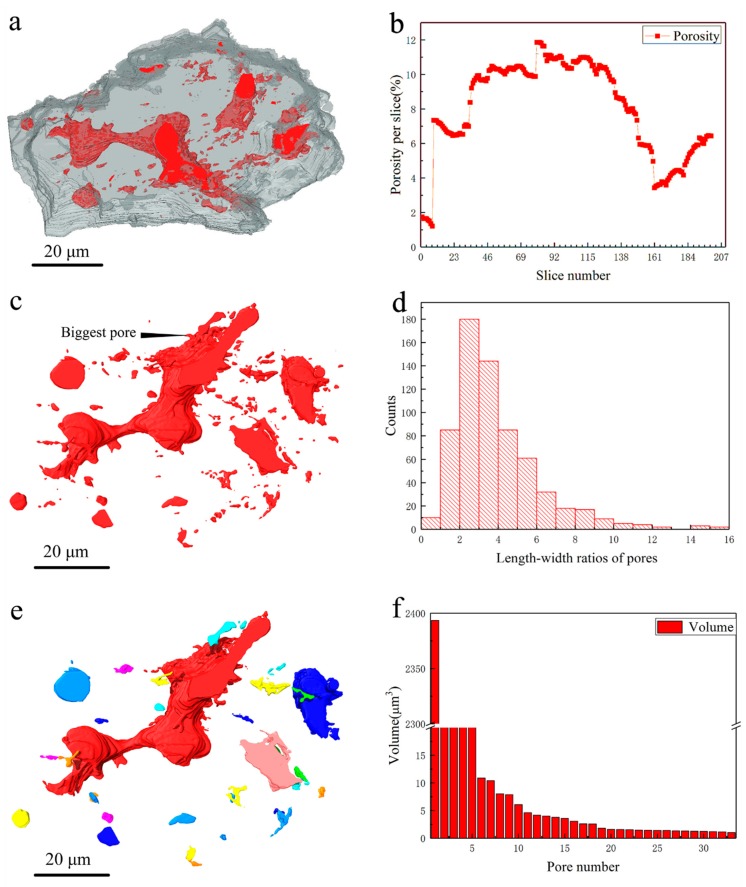
Characterization of the biggest C_3_S grain and pores within it: (**a**) 3D rendering image of the biggest C_3_S grain and the pores within it (labeled in red); (**b**) A profile of porosity per slice; (**c**) 3D rendering image of the pores (within the biggest C_3_S grain) alone; (**d**) A histogram (with step of 1) of length–width ratios of pores; (**e**) Segmented pores, bigger than 1μm^3^, rendered in different colors; (**f**) Volume distribution of pores bigger than 1 μm^3^.

The largest C_3_S grain was located, segmented individually from the rest of the particles, and displayed in Figure 4a, in which the pores that were rendered in red can be easily seen. The 3D spatial distribution of the pores in this large C_3_S grain is also shown in Figure 4c. Both Figure 4a,c show that the sizes and shapes of the pores in the grain are quite different from each other. The biggest pore, with a volume of 2.4 × 10^3^ μm^3^, as shown in the middle of Figure 4c, runs through the whole grain and has a high surface-area-to-volume ratio. This could play an important role in the hydration of C_3_S because it can increase the contact of C_3_S grains with water. As Figure 4b presents, the porosity per slice (defined as the area of pores within the grain against the whole area of the grain within the slice) within the single C_3_S grain varies significantly along the sectioning direction. It ranges between 1.2% and 11.9% (Figure 4b), and all the values are numerically presented in Supplementary Spreadsheet S2. Since the porosity represents morphometric parameters in the serial 2D slice images over the volume of the largest C_3_S grain, it can demonstrate the evolution of pores inside this C_3_S grain. For example, in slice 8, the porosity has a minimum value of 1.2%, and the porosity reaches 7.3% in slice 9, which indicates the appearance of new pore(s). 

Compared with 2D slice data only, after segmentation and rendering of the acquired 3D structural image, the real shape, volumes/sizes, orientation, and connection of the pores within the largest C_3_S grain can be obtained through the data analysis. This gives the quantitative information on the material porosity. In turn, it can help to reveal one of the most important characteristics of the microstructure of hardened cement paste: pore structure. From Supplementary Spreadsheet S3, it can be known that the pores within the C_3_S grain have a relatively wide size distribution, with about 5% of them being larger than 1 μm^3^, and the mean length, width, and volume of all the pores are 1.3 µm, 0.3 µm, and 4.8 μm^3^, respectively. Based on the statistical result obtained from Supplementary Spreadsheet S3, the total volume of the pores in the grain is 3.1 × 10^3^ μm^3^ and the porosity of the grain is about 8%. From Figure 4d, it can be seen that the LWRs of most of the pores are between 1 and 5. With further analysis of the porosity, the independent pores larger than 1 μm^3^ are displayed in Figure 4e in different colors, and about 85% of them have volumes between 1 μm^3^ and 10 μm^3^ (Figure 4f). 

## 4. Conclusions

SBFSEM has been shown to be an effective method to investigate the 3D structure of hard condensed matters and materials such as raw C_3_S mineral particles. This method provides high resolution and enables nanoscale details of the C_3_S sample, as small as about 4.8 × 10^−4^ μm^3^ (likely 60 × 60 × 133 nm^3^), to be obtained. 

In the epoxy-embedded C_3_S grain samples, the average volume of the C_3_S grains is 25 μm^3^ and more than 95% of the C_3_S grains are smaller than 1 μm^3^. This indicates that the sample is composed of large amounts of fine particles and relatively small amount of larger particles, which results in a large surface area of C_3_S and will be beneficial to its hydration process. The pore structure within the largest raw C_3_S grain is very rich: the mean volume of the pores is 4.8 μm^3^ and more than half of the pores have LWR values around 3. The largest pore is 2.4 × 10^3^ μm^3^, which penetrates through the whole largest C_3_S grain. This quantitative information can also be used for further analysis of the relationship between the kinetics of C_3_S hydration process and their spatial nanostructure and microstructure, including the porous structure within the ingredient particles. 

The results prove that SBFSEM could be used for investigation of the internal pore structure of the cement materials such as raw C_3_S grains, and it could be applied to study the nanoscale 3D spatial structure of hard condensed matters and materials in future. 

## Supplementary Materials

The following supplementary materials are available online at http://www.mdpi.com/1996-1944/11/7/1110/s1, Spreadsheet S1: Volumes, areas, lengths, widths, and length–width ratios of C_3_S grains; Spreadsheet S2: Areas of C_3_S and pores per slice image and the porosity per slice; Spreadsheet S3: Volumes, areas, lengths, widths, and length–width ratios of pores within the largest C_3_S grain.
