# Supplementary material for: Investigation of Three-Dimensional Microstructure of Tricalcium Silicate (C3S) by Electron Microscopy
Source: Materials (Basel). 2018 Jun 29;11(7):1110. doi: 10.3390/ma11071110 (PMC6073500; doi:10.3390/ma11071110)
Supplement: Supplementary file 1 [file materials-11-01110-s001.zip › Supplementary Spreadsheet/Supplementary Spreadsheet S1_proofread.docx]

Spreadsheet S1: Volumes, areas, lengths, widths and length-width ratios of C_3_S grains

| index | Volume(μm^3^) | Area (μm^2^)) | Length(μm) (μm) | Width(μm) μm) | Length-width ratio | |
| --- | --- | --- | --- | --- | --- | --- |
| 1 | 44213.1 | 37242.7 | 129.046 | 19.0391 | 6.77795 |  |
| 2 | 0.322694 | 3.75261 | 3.0194 | 0.463992 | 6.50742 |  |
| 3 | 0.0019208 | 0.0728226 | 0.279904 | 0.069318 | 4.03799 |  |
| 4 | 1.61251 | 13.1939 | 3.79805 | 1.58306 | 2.39919 |  |
| 5 | 0.0854756 | 1.24678 | 1.23598 | 0.346588 | 3.56613 |  |
| 6 | 0.0009604 | 0.0381041 | 0.208251 | 0.092506 | 2.25122 |  |
| 7 | 0.0004802 | 0.019606 | 0.059534 | 0.059534 | 1 |  |
| 8 | 0.0398566 | 0.843505 | 1.40001 | 0.299333 | 4.67709 |  |
| 9 | 0.0388962 | 0.759 | 0.905728 | 0.188385 | 4.80784 |  |
| 10 | 0.0038416 | 0.141675 | 0.411104 | 0.079101 | 5.19718 |  |
| 11 | 0.012005 | 0.303215 | 0.710671 | 0.138635 | 5.1262 |  |
| 12 | 0.114288 | 1.38691 | 1.24165 | 0.317237 | 3.91395 |  |
| 13 | 0.0033614 | 0.124869 | 0.356027 | 0.079101 | 4.50089 |  |
| 14 | 70.6 | 333.089 | 16.5048 | 4.18111 | 3.94746 |  |
| 15 | 0.0129654 | 0.340114 | 0.643925 | 0.138635 | 4.64474 |  |
| 16 | 0.0067228 | 0.245245 | 0.637737 | 0.079102 | 8.06226 |  |
| 17 | 2.57483 | 18.4832 | 5.85774 | 0.847219 | 6.91409 |  |
| 18 | 0.02401 | 0.490058 | 0.818302 | 0.274171 | 2.98464 |  |
| 19 | 0.0014406 | 0.0560173 | 0.279904 | 0.069318 | 4.03799 |  |
| 20 | 0.0004802 | 0.019606 | 0.059534 | 0.059534 | 1 |  |
| 21 | 0.0643468 | 0.895789 | 0.945325 | 0.287885 | 3.28368 |  |
| 22 | 0.009604 | 0.273732 | 0.511804 | 0.128851 | 3.97205 |  |
| 23 | 0.0129654 | 0.294734 | 0.509023 | 0.225735 | 2.25496 |  |
| 24 | 0.106124 | 1.40441 | 1.40938 | 0.356372 | 3.95481 |  |
| 25 | 0.0033614 | 0.114329 | 0.356025 | 0.119068 | 2.9901 |  |
| 26 | 0.002401 | 0.0907357 | 0.355335 | 0.069318 | 5.12619 |  |
| 27 | 1.58754 | 13.4163 | 5.00356 | 0.649882 | 7.69919 |  |
| 28 | 0.502289 | 4.38703 | 1.91113 | 0.514574 | 3.71401 |  |
| 29 | 0.0763518 | 1.24795 | 1.27869 | 0.247087 | 5.17506 |  |
| 30 | 0.133015 | 3.94092 | 2.01058 | 0.285391 | 7.04499 |  |
| 31 | 0.0475398 | 0.932654 | 1.05257 | 0.207953 | 5.06157 |  |
| 32 | 6.18498 | 51.2526 | 8.00417 | 1.27457 | 6.27989 |  |
| 33 | 0.033614 | 1.17793 | 1.30603 | 0.157371 | 8.29907 |  |
| 34 | 0.021609 | 0.506505 | 0.796001 | 0.217737 | 3.6558 |  |
| 35 | 0.258348 | 2.96886 | 1.99248 | 0.39717 | 5.01671 |  |
| 36 | 0.23914 | 3.4653 | 1.84254 | 0.309117 | 5.96068 |  |
| 37 | 0.0067228 | 0.244075 | 0.511805 | 0.088885 | 5.75809 |  |
| 38 | 0.0806736 | 2.79584 | 1.80241 | 0.225856 | 7.98035 |  |
| 39 | 0.0038416 | 0.120009 | 0.356028 | 0.119068 | 2.99014 |  |
| 40 | 0.0273714 | 0.564092 | 0.789191 | 0.198169 | 3.98241 |  |
| 41 | 0.543586 | 6.30115 | 3.47292 | 0.406122 | 8.55143 |  |
| 42 | 0.0057624 | 0.143611 | 0.374719 | 0.168818 | 2.21966 |  |
| 43 | 0.0052822 | 0.172579 | 0.497696 | 0.119068 | 4.17994 |  |
| 44 | 0.148862 | 2.06091 | 1.89832 | 0.376771 | 5.0384 |  |
| 45 | 1.06076 | 12.2319 | 5.04086 | 1.18821 | 4.2424 |  |
| 46 | 0.0014406 | 0.0566022 | 0.308397 | 0.059534 | 5.18018 |  |
| 47 | 0.0038416 | 0.14226 | 0.416545 | 0.069318 | 6.00923 |  |
| 48 | 0.130614 | 1.94888 | 2.04853 | 0.40666 | 5.03746 |  |
| 49 | 0.114768 | 2.05166 | 1.97935 | 0.267487 | 7.39981 |  |
| 50 | 0.21705 | 2.49138 | 1.91476 | 0.502947 | 3.80708 |  |
| 51 | 0.0134456 | 0.365592 | 0.708391 | 0.138635 | 5.10975 |  |
| 52 | 1.63796 | 12.2638 | 3.53582 | 0.63281 | 5.58749 |  |
| 53 | 0.0316932 | 0.633553 | 0.867352 | 0.2937 | 2.95319 |  |
| 54 | 0.0533022 | 0.86112 | 1.1154 | 0.238135 | 4.68387 |  |
| 55 | 0.0043218 | 0.146011 | 0.411102 | 0.119068 | 3.45267 |  |
| 56 | 1.57938 | 10.5607 | 3.14036 | 0.95171 | 3.2997 |  |
| 57 | 0.0038416 | 0.120009 | 0.356215 | 0.119068 | 2.9917 |  |
| 58 | 5.88821 | 47.5676 | 7.37313 | 1.95982 | 3.76214 |  |
| 59 | 0.0057624 | 0.212742 | 0.703953 | 0.088885 | 7.91982 |  |
| 60 | 0.0715498 | 1.05186 | 0.947434 | 0.278102 | 3.40679 |  |
| 61 | 0.0547428 | 0.829689 | 0.890968 | 0.381255 | 2.33694 |  |
| 62 | 0.0436982 | 0.969133 | 1.11623 | 0.188386 | 5.92525 |  |
| 63 | 0.0206486 | 0.472354 | 0.801241 | 0.178602 | 4.48619 |  |
| 64 | 0.0067228 | 0.18333 | 0.445485 | 0.159034 | 2.80119 |  |
| 65 | 0.0038416 | 0.142782 | 0.416545 | 0.079101 | 5.26597 |  |
| 66 | 0.0067228 | 0.245245 | 0.627594 | 0.088885 | 7.06074 |  |
| 67 | 0.0019208 | 0.0728226 | 0.279906 | 0.069318 | 4.03802 |  |
| 68 | 0.0067228 | 0.193563 | 0.426044 | 0.128851 | 3.30648 |  |
| 69 | 0.0441784 | 0.760166 | 1.04357 | 0.312579 | 3.33857 |  |
| 70 | 0.140699 | 1.69479 | 1.29757 | 0.34742 | 3.73487 |  |
| 71 | 0.337581 | 4.75211 | 3.65343 | 0.623858 | 5.85619 |  |
| 72 | 0.162788 | 2.34309 | 1.60736 | 0.306622 | 5.24215 |  |
| 73 | 0.014406 | 0.398713 | 0.707169 | 0.148419 | 4.76469 |  |
| 74 | 0.0268912 | 0.637623 | 1.05257 | 0.198169 | 5.31148 |  |
| 75 | 1.6927 | 10.2014 | 2.93694 | 1.13031 | 2.59834 |  |
| 76 | 0.0014406 | 0.0566022 | 0.308397 | 0.059534 | 5.18019 |  |
| 77 | 0.187278 | 6.44336 | 3.13913 | 0.362828 | 8.65186 |  |
| 78 | 0.012005 | 0.317499 | 0.628515 | 0.138635 | 4.53359 |  |
| 79 | 0.0921984 | 3.20062 | 2.95044 | 0.34326 | 8.59533 |  |
| 80 | 0.0086436 | 0.232584 | 0.733872 | 0.214032 | 3.4288 |  |
| 81 | 0.251625 | 3.34847 | 2.12164 | 0.793677 | 2.67317 |  |
| 82 | 0.0398566 | 0.825697 | 1.14137 | 0.198169 | 5.75956 |  |
| 83 | 0.429779 | 14.6697 | 4.20437 | 0.411746 | 10.2111 |  |
| 84 | 1.66341 | 11.1418 | 4.13348 | 0.991905 | 4.16721 |  |
| 85 | 0.0172872 | 0.371735 | 0.638659 | 0.23148 | 2.75902 |  |
| 86 | 1.15536 | 8.90806 | 3.03396 | 0.514574 | 5.89606 |  |
| 87 | 1.98323 | 18.91 | 4.98272 | 0.799133 | 6.23516 |  |
| 88 | 0.0225694 | 0.472339 | 0.767073 | 0.229184 | 3.34698 |  |
| 89 | 0.0897974 | 1.3871 | 1.20044 | 0.356371 | 3.3685 |  |
| 90 | 0.0014406 | 0.0560173 | 0.275414 | 0.069318 | 3.97323 |  |
| 91 | 0.0038416 | 0.14226 | 0.416553 | 0.069318 | 6.00934 |  |
| 92 | 10.6263 | 61.8335 | 6.76392 | 2.01187 | 3.362 |  |
| 93 | 0.0422576 | 0.78794 | 1.07785 | 0.352239 | 3.06 |  |
| 94 | 0.577681 | 5.6531 | 2.51231 | 0.425689 | 5.90175 |  |
| 95 | 0.0345744 | 0.733882 | 0.995327 | 0.237304 | 4.19432 |  |
| 96 | 1.32007 | 11.1724 | 3.75776 | 0.793508 | 4.73563 |  |
| 97 | 0.040817 | 1.42964 | 1.72922 | 0.206289 | 8.3825 |  |
| 98 | 6.65749 | 34.6294 | 6.30064 | 1.54372 | 4.08146 |  |
| 99 | 0.0398566 | 0.66819 | 0.776794 | 0.308285 | 2.51973 |  |
| 100 | 0.0052822 | 0.156786 | 0.416499 | 0.119068 | 3.49799 |  |
| 101 | 3.02046 | 20.3975 | 5.01692 | 1.08951 | 4.60474 |  |
| 102 | 0.0115248 | 0.331424 | 0.695063 | 0.138635 | 5.01362 |  |
| 103 | 8.95285 | 56.7571 | 14.6547 | 1.71797 | 8.53026 |  |
| 104 | 1.45405 | 10.1802 | 3.50935 | 0.602627 | 5.82342 |  |
| 105 | 0.0019208 | 0.0728226 | 0.279906 | 0.069318 | 4.03802 |  |
| 106 | 0.0004802 | 0.019606 | 0.059534 | 0.059534 | 1 |  |
| 107 | 0.088837 | 1.37995 | 1.30895 | 0.366987 | 3.56674 |  |
| 108 | 0.0158466 | 0.341877 | 0.546791 | 0.208785 | 2.61892 |  |
| 109 | 0.0523418 | 0.881414 | 0.997188 | 0.237304 | 4.20216 |  |
| 110 | 0.272273 | 4.24451 | 3.6221 | 0.502296 | 7.21109 |  |
| 111 | 0.0038416 | 0.109399 | 0.364011 | 0.128852 | 2.82505 |  |
| 112 | 0.0028812 | 0.0984743 | 0.355335 | 0.109284 | 3.25149 |  |
| 113 | 0.377917 | 3.40849 | 1.80015 | 0.645089 | 2.79054 |  |
| 114 | 0.0225694 | 0.536278 | 0.900189 | 0.217737 | 4.1343 |  |
| 115 | 0.0009604 | 0.032799 | 0.109284 | 0.143766 | 0.760151 |  |
| 116 | 0.0307328 | 0.615983 | 0.910008 | 0.188385 | 4.83057 |  |
| 117 | 0.0115248 | 0.28641 | 0.630974 | 0.148418 | 4.25133 |  |
| 118 | 0.0019208 | 0.0603798 | 0.228965 | 0.140504 | 1.6296 |  |
| 119 | 0.0086436 | 0.215665 | 0.449157 | 0.168818 | 2.6606 |  |
| 120 | 0.0019208 | 0.0603798 | 0.228973 | 0.140505 | 1.62964 |  |
| 121 | 0.004802 | 0.132076 | 0.365525 | 0.205169 | 1.78158 |  |
| 122 | 0.0230496 | 0.475035 | 0.724943 | 0.188385 | 3.84819 |  |
| 123 | 0.009604 | 0.235584 | 0.521535 | 0.119068 | 4.38014 |  |
| 124 | 0.0557032 | 1.04011 | 1.50765 | 0.359867 | 4.18946 |  |
| 125 | 0.0701092 | 1.0601 | 1.01862 | 0.258535 | 3.93996 |  |
| 126 | 0.0009604 | 0.0381041 | 0.213139 | 0.059534 | 3.58013 |  |
| 127 | 0.0110446 | 0.263774 | 0.576743 | 0.208499 | 2.76616 |  |
| 128 | 0.0398566 | 0.7966 | 1.13404 | 0.238135 | 4.76218 |  |
| 129 | 0.0139258 | 0.31866 | 0.580782 | 0.178602 | 3.25182 |  |
| 130 | 0.0254506 | 0.547525 | 0.868251 | 0.238135 | 3.64604 |  |
| 131 | 0.0028812 | 0.0901946 | 0.286624 | 0.119068 | 2.40724 |  |
| 132 | 0.0009604 | 0.0381041 | 0.213138 | 0.059534 | 3.58011 |  |
| 133 | 0.0004802 | 0.019606 | 0.059534 | 0.059534 | 1 |  |
| 134 | 0.0355348 | 0.648468 | 0.841618 | 0.218568 | 3.8506 |  |
| 135 | 0.0340942 | 0.708314 | 0.966231 | 0.257703 | 3.7494 |  |
| 136 | 0.0028812 | 0.101477 | 0.361977 | 0.138219 | 2.61887 |  |
| 137 | 55.1697 | 251.042 | 13.8327 | 5.81698 | 2.37799 |  |
| 138 | 0.0014406 | 0.0560173 | 0.279902 | 0.069318 | 4.03796 |  |
| 139 | 0.0028812 | 0.0984465 | 0.30472 | 0.119068 | 2.55921 |  |
| 140 | 0.0326536 | 0.606806 | 0.802384 | 0.238136 | 3.36944 |  |
| 141 | 0.111887 | 1.6029 | 1.40508 | 0.453859 | 3.09586 |  |
| 142 | 0.198803 | 2.17875 | 1.51936 | 0.642169 | 2.36598 |  |
| 143 | 0.19304 | 2.14671 | 1.21059 | 0.725022 | 1.66973 |  |
| 144 | 0.0115248 | 0.279308 | 0.574078 | 0.168818 | 3.40057 |  |
| 145 | 0.0019208 | 0.0603798 | 0.219564 | 0.127091 | 1.72761 |  |
| 146 | 0.0067228 | 0.196609 | 0.508643 | 0.119068 | 4.27188 |  |
| 147 | 0.004802 | 0.128691 | 0.366904 | 0.119068 | 3.08146 |  |
| 148 | 0.0297724 | 0.63209 | 0.845653 | 0.167986 | 5.03407 |  |
| 149 | 0.004802 | 0.134344 | 0.360258 | 0.159034 | 2.26529 |  |
| 150 | 0.0244902 | 0.528284 | 0.869423 | 0.168818 | 5.15006 |  |
| 151 | 0.0321734 | 0.777342 | 1.67124 | 0.324311 | 5.15319 |  |
| 152 | 0.002401 | 0.0758411 | 0.28506 | 0.119068 | 2.3941 |  |
| 153 | 0.0153664 | 0.354703 | 0.576924 | 0.148419 | 3.88714 |  |
| 154 | 0.014406 | 0.317506 | 0.635238 | 0.188385 | 3.37201 |  |
| 155 | 0.004802 | 0.128691 | 0.359157 | 0.119068 | 3.01641 |  |
| 156 | 0.0105644 | 0.262241 | 0.63163 | 0.138635 | 4.55605 |  |
| 157 | 0.403368 | 3.32566 | 1.67437 | 0.535805 | 3.12496 |  |
| 158 | 1.08333 | 8.79258 | 3.08483 | 1.15238 | 2.67693 |  |
| 159 | 0.186318 | 3.5336 | 2.84833 | 0.226689 | 12.565 |  |
| 160 | 0.0753914 | 0.974398 | 0.86609 | 0.467319 | 1.85332 |  |
| 161 | 0.0038416 | 0.118883 | 0.415575 | 0.138219 | 3.00665 |  |
| 162 | 0.0009604 | 0.0381041 | 0.213139 | 0.059534 | 3.58013 |  |
| 163 | 0.007203 | 0.179445 | 0.426322 | 0.168818 | 2.52533 |  |
| 164 | 0.0004802 | 0.019606 | 0.059534 | 0.059534 | 1 |  |
| 165 | 0.0019208 | 0.0603798 | 0.219566 | 0.127091 | 1.72763 |  |
| 166 | 0.0614656 | 0.92587 | 0.940352 | 0.287886 | 3.26641 |  |
| 167 | 0.0153664 | 0.360846 | 0.740036 | 0.128851 | 5.74332 |  |
| 168 | 0.0206486 | 0.385712 | 0.566551 | 0.218568 | 2.59211 |  |
| 169 | 0.0028812 | 0.0848895 | 0.288523 | 0.119068 | 2.42318 |  |
| 170 | 0.26363 | 3.32073 | 2.42508 | 0.584815 | 4.14675 |  |
| 171 | 0.031213 | 0.525884 | 0.657541 | 0.228352 | 2.87951 |  |
| 172 | 0.0220892 | 0.415711 | 0.595947 | 0.218569 | 2.72659 |  |
| 173 | 0.0398566 | 0.607567 | 0.719961 | 0.336895 | 2.13705 |  |
| 174 | 2.04901 | 13.6082 | 4.43919 | 0.873441 | 5.08241 |  |
| 175 | 0.230976 | 2.37307 | 1.53915 | 0.468151 | 3.28772 |  |
| 176 | 0.318373 | 2.57751 | 1.5451 | 0.565988 | 2.72991 |  |
| 177 | 178.615 | 598.19 | 24.2341 | 6.96879 | 3.47752 |  |
| 178 | 0.0110446 | 0.247598 | 0.498912 | 0.178602 | 2.79343 |  |
| 179 | 0.0009604 | 0.032799 | 0.109284 | 0.143766 | 0.760153 |  |
| 180 | 0.0374556 | 0.656783 | 0.811223 | 0.188385 | 4.30619 |  |
| 181 | 0.0753914 | 0.957998 | 0.974051 | 0.493143 | 1.97519 |  |
| 182 | 0.0043218 | 0.119642 | 0.359159 | 0.119068 | 3.01643 |  |
| 183 | 0.179595 | 2.35863 | 1.63989 | 0.349083 | 4.6977 |  |
| 184 | 1.00602 | 9.25491 | 2.85422 | 1.08472 | 2.63129 |  |
| 185 | 0.0422576 | 0.617045 | 0.73477 | 0.327852 | 2.24116 |  |
| 186 | 0.155105 | 2.06883 | 2.40407 | 0.407786 | 5.89541 |  |
| 187 | 0.0100842 | 0.272843 | 0.76328 | 0.148419 | 5.14274 |  |
| 188 | 0.043218 | 0.895403 | 1.20123 | 0.148419 | 8.09352 |  |
| 189 | 0.0091238 | 0.219652 | 0.445899 | 0.159034 | 2.80379 |  |
| 190 | 0.031213 | 0.529524 | 0.633458 | 0.258535 | 2.45018 |  |
| 191 | 0.0801934 | 1.16651 | 1.16041 | 0.332197 | 3.49314 |  |
| 192 | 11.8201 | 70.4769 | 11.3228 | 2.75424 | 4.11104 |  |
| 193 | 0.0657874 | 1.04838 | 1.04357 | 0.31491 | 3.31386 |  |
| 194 | 1.23844 | 13.8973 | 5.26526 | 1.38885 | 3.7911 |  |
| 195 | 0.50469 | 3.93322 | 1.84 | 0.545589 | 3.3725 |  |
| 196 | 0.0921984 | 1.18541 | 1.25097 | 0.415998 | 3.00714 |  |
| 197 | 50.3413 | 139.546 | 11.5188 | 3.64288 | 3.16201 |  |
| 198 | 0.076832 | 1.07344 | 0.965513 | 0.348251 | 2.77246 |  |
| 199 | 0.0004802 | 0.019606 | 0.059534 | 0.059534 | 1 |  |
| 200 | 0.237219 | 3.62347 | 2.68532 | 0.354708 | 7.57052 |  |
| 201 | 0.0124852 | 0.28209 | 0.574078 | 0.246593 | 2.32804 |  |
| 202 | 0.269872 | 3.09675 | 1.88018 | 0.541424 | 3.47266 |  |
| 203 | 0.363031 | 3.49899 | 2.06219 | 0.44692 | 4.61423 |  |
| 204 | 0.019208 | 0.422656 | 0.657539 | 0.138635 | 4.74295 |  |
| 205 | 0.0009604 | 0.0381041 | 0.208249 | 0.092504 | 2.25125 |  |
| 206 | 2.00628 | 15.833 | 4.85138 | 1.32775 | 3.65385 |  |
| 207 | 0.921024 | 11.8407 | 5.79591 | 0.797417 | 7.26836 |  |
| 208 | 0.0393764 | 0.813055 | 0.997823 | 0.218568 | 4.56528 |  |
| 209 | 0.0105644 | 0.242076 | 0.495942 | 0.168818 | 2.93773 |  |
| 210 | 0.007203 | 0.179811 | 0.403654 | 0.168818 | 2.39106 |  |
| 211 | 0.0009604 | 0.032799 | 0.109284 | 0.143766 | 0.760153 |  |
| 212 | 0.0019208 | 0.0603798 | 0.219567 | 0.127085 | 1.72772 |  |
| 213 | 0.0763518 | 1.66411 | 2.71711 | 0.373443 | 7.27584 |  |
| 214 | 0.0062426 | 0.185109 | 0.480116 | 0.182203 | 2.63506 |  |
| 215 | 0.0086436 | 0.271525 | 0.728189 | 0.158203 | 4.60289 |  |
| 216 | 0.0653072 | 1.34635 | 1.83867 | 0.247087 | 7.44137 |  |
| 217 | 0.0182476 | 0.404193 | 0.729709 | 0.179434 | 4.06674 |  |
| 218 | 0.0028812 | 0.0901946 | 0.293123 | 0.109284 | 2.68222 |  |
| 219 | 0.0057624 | 0.1532 | 0.423217 | 0.128851 | 3.28453 |  |
| 220 | 0.0028812 | 0.0901078 | 0.361977 | 0.198654 | 1.82215 |  |
| 221 | 0.0105644 | 0.255276 | 0.567358 | 0.168818 | 3.36077 |  |
| 222 | 0.0019208 | 0.0603798 | 0.219566 | 0.127085 | 1.72771 |  |
| 223 | 0.0734706 | 1.12063 | 1.0992 | 0.317236 | 3.46492 |  |
| 224 | 0.0196882 | 0.362093 | 0.531266 | 0.218568 | 2.43067 |  |
| 225 | 0.0052822 | 0.166583 | 0.520028 | 0.149251 | 3.48426 |  |
| 226 | 0.0086436 | 0.240794 | 0.707997 | 0.243444 | 2.90825 |  |
| 227 | 0.0148862 | 0.364563 | 0.838462 | 0.245201 | 3.41949 |  |
| 228 | 0.0009604 | 0.032799 | 0.109284 | 0.143766 | 0.760152 |  |
| 229 | 0.0086436 | 0.213222 | 0.495942 | 0.128851 | 3.84895 |  |
| 230 | 0.0533022 | 1.24919 | 1.67777 | 0.265823 | 6.31161 |  |
| 231 | 0.0249704 | 0.613008 | 1.46298 | 0.238828 | 6.12567 |  |
| 232 | 0.0100842 | 0.252869 | 0.512966 | 0.188385 | 2.72296 |  |
| 233 | 0.0038416 | 0.115541 | 0.409153 | 0.152027 | 2.69133 |  |
| 234 | 0.0038416 | 0.122461 | 0.361977 | 0.139299 | 2.59857 |  |
| 235 | 0.0009604 | 0.032799 | 0.109284 | 0.143766 | 0.760151 |  |
| 236 | 0.0043218 | 0.130166 | 0.36401 | 0.128851 | 2.82504 |  |
| 237 | 0.0038416 | 0.14226 | 0.426036 | 0.088885 | 4.79314 |  |
| 238 | 0.0076832 | 0.225471 | 0.794415 | 0.163833 | 4.84893 |  |
| 239 | 0.0115248 | 0.254875 | 0.435763 | 0.257884 | 1.68977 |  |
| 240 | 0.452829 | 4.02767 | 1.9132 | 0.425689 | 4.49435 |  |
| 241 | 0.0206486 | 0.408774 | 0.694594 | 0.278458 | 2.49443 |  |
| 242 | 0.0038416 | 0.104181 | 0.293123 | 0.119068 | 2.46182 |  |
| 243 | 0.016807 | 0.431872 | 0.995938 | 0.236343 | 4.21396 |  |
| 244 | 0.004802 | 0.128691 | 0.366904 | 0.119068 | 3.08147 |  |
| 245 | 0.0028812 | 0.0848895 | 0.288523 | 0.119068 | 2.42318 |  |
| 246 | 0.016807 | 0.371314 | 0.751282 | 0.228996 | 3.28076 |  |
| 247 | 0.0009604 | 0.032799 | 0.109284 | 0.143766 | 0.760153 |  |
| 248 | 0.0177674 | 0.43057 | 0.847151 | 0.279679 | 3.02901 |  |
| 249 | 0.0633864 | 1.07069 | 1.30183 | 0.267487 | 4.86689 |  |
| 250 | 0.0009604 | 0.032799 | 0.109284 | 0.143766 | 0.760153 |  |
| 251 | 0.0004802 | 0.019606 | 0.059534 | 0.059534 | 1 |  |
| 252 | 0.004802 | 0.127381 | 0.366905 | 0.183283 | 2.00185 |  |
| 253 | 0.0076832 | 0.223639 | 0.602093 | 0.182203 | 3.30452 |  |
| 254 | 0.0028812 | 0.0901078 | 0.361977 | 0.198654 | 1.82215 |  |
| 255 | 0.007203 | 0.182273 | 0.423218 | 0.128852 | 3.28454 |  |
| 256 | 0.0153664 | 0.34735 | 0.662407 | 0.233211 | 2.84038 |  |
| 257 | 0.0321734 | 0.60464 | 0.811708 | 0.188385 | 4.30877 |  |
| 258 | 0.0393764 | 0.736647 | 0.946067 | 0.22752 | 4.15817 |  |
| 259 | 0.0067228 | 0.193825 | 0.609438 | 0.187821 | 3.24478 |  |
| 260 | 0.0158466 | 0.344929 | 0.623735 | 0.178602 | 3.49232 |  |
| 261 | 0.0249704 | 0.556241 | 0.980017 | 0.187553 | 5.22527 |  |
| 262 | 0.205045 | 2.85566 | 1.90888 | 0.455872 | 4.18732 |  |
| 263 | 26.2362 | 112.944 | 7.70566 | 4.50687 | 1.70976 |  |
| 264 | 0.0019208 | 0.0603798 | 0.228969 | 0.140504 | 1.62962 |  |
| 265 | 0.0019208 | 0.0603798 | 0.228969 | 0.140504 | 1.62962 |  |
| 266 | 0.0028812 | 0.0879606 | 0.312681 | 0.153397 | 2.03838 |  |
| 267 | 0.0470596 | 0.965155 | 1.12496 | 0.22752 | 4.94443 |  |
| 268 | 0.0009604 | 0.032799 | 0.109284 | 0.143766 | 0.760153 |  |
| 269 | 0.0028812 | 0.0901946 | 0.293121 | 0.109284 | 2.68219 |  |
| 270 | 0.0057624 | 0.147982 | 0.359161 | 0.119067 | 3.01646 |  |
| 271 | 0.0009604 | 0.0381041 | 0.208251 | 0.092506 | 2.25121 |  |
| 272 | 0.0052822 | 0.174552 | 0.601485 | 0.143513 | 4.19117 |  |
| 273 | 0.0100842 | 0.250584 | 0.576926 | 0.148419 | 3.88715 |  |
| 274 | 0.0019208 | 0.0603798 | 0.228971 | 0.140504 | 1.62964 |  |
| 275 | 0.0134456 | 0.322263 | 0.706133 | 0.128851 | 5.48022 |  |
| 276 | 0.0009604 | 0.032799 | 0.109285 | 0.143766 | 0.760158 |  |
| 277 | 0.0009604 | 0.032799 | 0.109284 | 0.143766 | 0.760151 |  |
| 278 | 0.0038416 | 0.104181 | 0.293123 | 0.119068 | 2.46181 |  |
| 279 | 0.0067228 | 0.17771 | 0.498648 | 0.128851 | 3.86995 |  |
| 280 | 0.0019208 | 0.0603798 | 0.228969 | 0.140504 | 1.62962 |  |
| 281 | 0.0028812 | 0.0848895 | 0.288523 | 0.119068 | 2.42318 |  |
| 282 | 0.0081634 | 0.223946 | 0.582106 | 0.159035 | 3.66024 |  |
| 283 | 0.0970004 | 1.8025 | 1.38061 | 0.226688 | 6.09033 |  |
| 284 | 0.050421 | 0.844 | 0.987563 | 0.208784 | 4.73006 |  |
| 285 | 0.105164 | 1.13268 | 0.985848 | 0.407785 | 2.41756 |  |
| 286 | 0.0038416 | 0.115541 | 0.409153 | 0.152027 | 2.69132 |  |
| 287 | 0.004802 | 0.13698 | 0.423218 | 0.128851 | 3.28455 |  |
| 288 | 0.0067228 | 0.185649 | 0.429405 | 0.128852 | 3.33255 |  |
| 289 | 0.439383 | 3.2727 | 1.80263 | 0.702258 | 2.5669 |  |
| 290 | 0.0158466 | 0.321215 | 0.536663 | 0.255606 | 2.09957 |  |
| 291 | 1.21683 | 8.1383 | 2.66624 | 1.12078 | 2.37892 |  |
| 292 | 0.0100842 | 0.277239 | 0.697873 | 0.159035 | 4.38818 |  |
| 293 | 0.0710696 | 0.849842 | 0.801501 | 0.387386 | 2.069 |  |
| 294 | 0.0326536 | 0.705619 | 0.947426 | 0.158203 | 5.98868 |  |
| 295 | 0.0340942 | 0.528946 | 0.616791 | 0.379763 | 1.62415 |  |
| 296 | 0.0230496 | 0.494142 | 0.975201 | 0.168818 | 5.77664 |  |
| 297 | 0.062426 | 0.83661 | 0.851337 | 0.297669 | 2.86001 |  |
| 298 | 0.0057624 | 0.1532 | 0.435763 | 0.138635 | 3.14323 |  |
| 299 | 0.0292922 | 0.587122 | 0.789505 | 0.178602 | 4.42047 |  |
| 300 | 0.235298 | 3.042 | 2.43347 | 0.365324 | 6.66114 |  |
| 301 | 0.0028812 | 0.0984743 | 0.356215 | 0.119068 | 2.9917 |  |
| 302 | 0.0028812 | 0.0848895 | 0.288523 | 0.119068 | 2.42318 |  |
| 303 | 0.0326536 | 0.564782 | 0.864284 | 0.379132 | 2.27964 |  |
| 304 | 0.0653072 | 1.11086 | 1.21153 | 0.356371 | 3.39964 |  |
| 305 | 0.130614 | 1.88837 | 1.79909 | 0.47923 | 3.75412 |  |
| 306 | 0.0038416 | 0.120009 | 0.361977 | 0.162357 | 2.22952 |  |
| 307 | 0.002401 | 0.0758411 | 0.288523 | 0.137894 | 2.09235 |  |
| 308 | 0.0014406 | 0.0497959 | 0.228969 | 0.130774 | 1.75088 |  |
| 309 | 0.171912 | 2.42574 | 1.54794 | 0.335972 | 4.60734 |  |
| 310 | 0.0172872 | 0.346649 | 0.555168 | 0.263305 | 2.10846 |  |
| 311 | 0.0033614 | 0.0977417 | 0.293123 | 0.119068 | 2.46181 |  |
| 312 | 0.0100842 | 0.253759 | 0.497697 | 0.159034 | 3.12949 |  |
| 313 | 0.0043218 | 0.135688 | 0.42891 | 0.119068 | 3.60223 |  |
| 314 | 0.0801934 | 1.04502 | 0.943792 | 0.358035 | 2.63603 |  |
| 315 | 0.0494606 | 0.803202 | 0.856196 | 0.327682 | 2.61288 |  |
| 316 | 0.351987 | 3.93198 | 2.20895 | 0.604082 | 3.6567 |  |
| 317 | 0.0004802 | 0.019606 | 0.059534 | 0.059534 | 1 |  |
| 318 | 0.0019208 | 0.0728226 | 0.279906 | 0.069318 | 4.03802 |  |
| 319 | 0.0028812 | 0.0913024 | 0.304722 | 0.119068 | 2.55923 |  |
| 320 | 0.0038416 | 0.106982 | 0.311093 | 0.203127 | 1.53152 |  |
| 321 | 0.202164 | 2.53084 | 2.141 | 0.493398 | 4.33931 |  |
| 322 | 0.158466 | 2.51039 | 1.60317 | 0.266655 | 6.01215 |  |
| 323 | 0.11957 | 1.69118 | 1.38609 | 0.366155 | 3.78553 |  |
| 324 | 0.0028812 | 0.0913088 | 0.308397 | 0.20322 | 1.51755 |  |
| 325 | 0.0019208 | 0.0603798 | 0.228971 | 0.140506 | 1.62962 |  |
| 326 | 0.0105644 | 0.248734 | 0.497696 | 0.138635 | 3.58998 |  |
| 327 | 0.0004802 | 0.019606 | 0.059534 | 0.059534 | 1 |  |
| 328 | 0.0081634 | 0.199653 | 0.426278 | 0.159034 | 2.68041 |  |
| 329 | 0.0307328 | 0.482694 | 0.613631 | 0.268318 | 2.28695 |  |
| 330 | 0.0436982 | 0.654073 | 0.752811 | 0.382741 | 1.96689 |  |
| 331 | 0.02401 | 0.523407 | 0.832462 | 0.167986 | 4.95554 |  |
| 332 | 0.0801934 | 0.994766 | 1.02588 | 0.377602 | 2.71682 |  |
| 333 | 0.098441 | 1.41288 | 1.14629 | 0.450755 | 2.54304 |  |
| 334 | 0.0412972 | 0.666547 | 0.914161 | 0.307453 | 2.97333 |  |
| 335 | 0.0465794 | 0.697639 | 0.856196 | 0.412205 | 2.07711 |  |
| 336 | 5.14102 | 26.1057 | 5.07017 | 1.90029 | 2.6681 |  |
| 337 | 0.0110446 | 0.259413 | 0.58624 | 0.199001 | 2.94592 |  |
| 338 | 0.019208 | 0.401852 | 0.73477 | 0.228351 | 3.21772 |  |
| 339 | 0.0163268 | 0.365705 | 0.630982 | 0.148419 | 4.25136 |  |
| 340 | 0.0019208 | 0.0603798 | 0.228969 | 0.140506 | 1.6296 |  |
| 341 | 0.0129654 | 0.3151 | 0.639967 | 0.168818 | 3.79087 |  |
| 342 | 0.0115248 | 0.292325 | 0.717386 | 0.188385 | 3.80809 |  |
| 343 | 0.0062426 | 0.172666 | 0.453041 | 0.195862 | 2.31306 |  |
| 344 | 0.531581 | 5.67039 | 2.538 | 1.22853 | 2.06589 |  |
| 345 | 0.014406 | 0.285543 | 0.461462 | 0.299643 | 1.54004 |  |
| 346 | 0.0019208 | 0.0603798 | 0.228969 | 0.140506 | 1.6296 |  |
| 347 | 0.172872 | 2.09635 | 1.95697 | 0.466488 | 4.19513 |  |
| 348 | 0.0086436 | 0.218117 | 0.461458 | 0.168818 | 2.73346 |  |
| 349 | 0.0235298 | 0.444922 | 0.626522 | 0.259952 | 2.41014 |  |
| 350 | 0.0009604 | 0.032799 | 0.109284 | 0.143766 | 0.760153 |  |
| 351 | 0.0590646 | 1.30438 | 1.89297 | 0.365323 | 5.18162 |  |
| 352 | 0.0009604 | 0.0381041 | 0.208249 | 0.092506 | 2.25119 |  |
| 353 | 0.0014406 | 0.0497959 | 0.237959 | 0.119736 | 1.98736 |  |
| 354 | 0.0019208 | 0.0603798 | 0.219567 | 0.127087 | 1.72769 |  |
| 355 | 0.002401 | 0.0826196 | 0.304722 | 0.119068 | 2.55923 |  |
| 356 | 0.0043218 | 0.115114 | 0.311091 | 0.203127 | 1.5315 |  |
| 357 | 0.0374556 | 0.846028 | 1.42841 | 0.226688 | 6.30119 |  |
| 358 | 0.0019208 | 0.0603798 | 0.228971 | 0.140504 | 1.62964 |  |
| 359 | 0.0038416 | 0.109399 | 0.318127 | 0.119068 | 2.67182 |  |
| 360 | 0.0557032 | 0.871488 | 1.28043 | 0.308285 | 4.15341 |  |
| 361 | 0.0028812 | 0.0879606 | 0.318127 | 0.134435 | 2.36641 |  |
| 362 | 0.0187278 | 0.414834 | 0.899404 | 0.25295 | 3.55566 |  |
| 363 | 0.04802 | 0.696092 | 0.817385 | 0.374786 | 2.18094 |  |
| 364 | 0.0043218 | 0.126071 | 0.403647 | 0.209025 | 1.93109 |  |
| 365 | 0.544067 | 7.51548 | 3.03731 | 0.537469 | 5.65114 |  |
| 366 | 0.227135 | 2.23052 | 1.31258 | 0.635305 | 2.06606 |  |
| 367 | 0.0038416 | 0.121117 | 0.356027 | 0.182203 | 1.95401 |  |
| 368 | 0.002401 | 0.0820625 | 0.288523 | 0.137895 | 2.09234 |  |
| 369 | 0.0009604 | 0.0381041 | 0.208249 | 0.092506 | 2.2512 |  |
| 370 | 0.0019208 | 0.0728226 | 0.279904 | 0.069318 | 4.03799 |  |
| 371 | 0.0019208 | 0.0603798 | 0.219567 | 0.127085 | 1.72771 |  |
| 372 | 0.0028812 | 0.0879606 | 0.312679 | 0.153397 | 2.03837 |  |
| 373 | 0.0004802 | 0.019606 | 0.059534 | 0.059534 | 1 |  |
| 374 | 0.0249704 | 0.459719 | 0.673735 | 0.24792 | 2.71755 |  |
| 375 | 0.0124852 | 0.307575 | 0.697532 | 0.179434 | 3.88741 |  |
| 376 | 0.0105644 | 0.257171 | 0.495942 | 0.168818 | 2.93773 |  |
| 377 | 0.0009604 | 0.0381041 | 0.208249 | 0.092506 | 2.25119 |  |
| 378 | 0.014406 | 0.328144 | 0.630978 | 0.178602 | 3.53288 |  |
| 379 | 0.0028812 | 0.0901946 | 0.293123 | 0.109284 | 2.68221 |  |
| 380 | 0.0566636 | 0.86395 | 1.00568 | 0.298501 | 3.36909 |  |
| 381 | 0.0028812 | 0.0999756 | 0.364572 | 0.109284 | 3.33599 |  |
| 382 | 0.0537824 | 0.837901 | 0.87807 | 0.432251 | 2.03139 |  |
| 383 | 0.0797132 | 1.15467 | 1.12021 | 0.278102 | 4.02807 |  |
| 384 | 0.016807 | 0.31967 | 0.511139 | 0.218569 | 2.33857 |  |
| 385 | 0.0014406 | 0.0497959 | 0.237959 | 0.119735 | 1.98738 |  |
| 386 | 0.0033614 | 0.106606 | 0.356028 | 0.119068 | 2.99014 |  |
| 387 | 0.0019208 | 0.0603798 | 0.228965 | 0.140504 | 1.6296 |  |
| 388 | 0.016807 | 0.371682 | 0.79282 | 0.238135 | 3.32928 |  |
| 389 | 0.0292922 | 0.629742 | 1.21442 | 0.218568 | 5.55626 |  |
| 390 | 0.0134456 | 0.28948 | 0.555171 | 0.199001 | 2.7898 |  |
| 391 | 0.0009604 | 0.032799 | 0.109284 | 0.143766 | 0.760153 |  |
| 392 | 0.0019208 | 0.0603798 | 0.228965 | 0.140506 | 1.62958 |  |
| 393 | 0.0014406 | 0.0560173 | 0.275414 | 0.069318 | 3.97323 |  |
| 394 | 0.0105644 | 0.244134 | 0.435763 | 0.168818 | 2.58126 |  |
| 395 | 0.0052822 | 0.141543 | 0.359159 | 0.119068 | 3.01643 |  |
| 396 | 0.111406 | 1.84149 | 2.29028 | 0.269151 | 8.50928 |  |
| 397 | 0.129654 | 1.73103 | 1.41902 | 0.459397 | 3.08887 |  |
| 398 | 0.0014406 | 0.0497959 | 0.228969 | 0.130774 | 1.75088 |  |
| 399 | 0.0067228 | 0.162904 | 0.337332 | 0.178602 | 1.88874 |  |
| 400 | 0.653072 | 5.57411 | 2.92439 | 0.971321 | 3.01073 |  |
| 401 | 0.0412972 | 0.836534 | 1.04502 | 0.178602 | 5.85114 |  |
| 402 | 0.0009604 | 0.0381041 | 0.208251 | 0.092506 | 2.25122 |  |
| 403 | 0.0086436 | 0.216293 | 0.529751 | 0.148419 | 3.5693 |  |
| 404 | 0.0019208 | 0.0603798 | 0.219568 | 0.127085 | 1.72772 |  |
| 405 | 0.0004802 | 0.019606 | 0.059534 | 0.059534 | 1 |  |
| 406 | 0.0009604 | 0.032799 | 0.109284 | 0.143766 | 0.760151 |  |
| 407 | 0.0629062 | 1.09895 | 1.26943 | 0.267487 | 4.74578 |  |
| 408 | 0.0052822 | 0.1291 | 0.312575 | 0.168818 | 1.85155 |  |
| 409 | 0.0931588 | 1.327 | 1.14376 | 0.267487 | 4.27597 |  |
| 410 | 0.163268 | 1.67247 | 1.27804 | 0.522898 | 2.44415 |  |
| 411 | 0.0148862 | 0.317384 | 0.566551 | 0.178602 | 3.17215 |  |
| 412 | 5.53479 | 31.8266 | 6.67559 | 1.58868 | 4.20197 |  |
| 413 | 0.129654 | 1.80896 | 1.20284 | 0.387386 | 3.10501 |  |
| 414 | 2.51673 | 15.0098 | 3.19144 | 1.42069 | 2.24639 |  |
| 415 | 0.0019208 | 0.0603798 | 0.228965 | 0.140506 | 1.62958 |  |
| 416 | 0.007203 | 0.180946 | 0.423218 | 0.178602 | 2.36962 |  |
| 417 | 0.002401 | 0.0773766 | 0.318127 | 0.127087 | 2.50323 |  |
| 418 | 0.0067228 | 0.169421 | 0.361977 | 0.128852 | 2.80925 |  |
| 419 | 0.0115248 | 0.307324 | 0.902566 | 0.119068 | 7.58025 |  |
| 420 | 0.639146 | 5.86469 | 2.43261 | 0.891027 | 2.73012 |  |
| 421 | 0.0033614 | 0.108108 | 0.359161 | 0.119068 | 3.01644 |  |
| 422 | 0.0009604 | 0.0381041 | 0.213139 | 0.059534 | 3.58013 |  |
| 423 | 0.105164 | 1.9603 | 2.12517 | 0.276439 | 7.68768 |  |
| 424 | 0.570478 | 5.60279 | 2.61313 | 0.768226 | 3.40152 |  |
| 425 | 0.0009604 | 0.032799 | 0.109284 | 0.143766 | 0.760155 |  |
| 426 | 0.0393764 | 0.703818 | 1.03334 | 0.297292 | 3.47585 |  |
| 427 | 0.0115248 | 0.293704 | 0.695063 | 0.159034 | 4.37052 |  |
| 428 | 5.63659 | 31.2535 | 5.33623 | 1.66382 | 3.20721 |  |
| 429 | 1.08333 | 9.24892 | 2.96847 | 0.73231 | 4.05357 |  |
| 430 | 0.150783 | 1.70184 | 1.31155 | 0.455872 | 2.87701 |  |
| 431 | 2.56043 | 16.2643 | 3.80665 | 1.17028 | 3.25277 |  |
| 432 | 0.549349 | 5.28834 | 3.29533 | 0.900426 | 3.65975 |  |
| 433 | 0.0081634 | 0.193431 | 0.413385 | 0.168818 | 2.4487 |  |
| 434 | 0.424977 | 3.83835 | 1.8242 | 0.754373 | 2.41817 |  |
| 435 | 0.837949 | 7.15459 | 3.74556 | 0.535805 | 6.99052 |  |
| 436 | 0.0350546 | 0.528211 | 0.683388 | 0.308285 | 2.21674 |  |
| 437 | 0.036015 | 0.579291 | 0.704352 | 0.228352 | 3.0845 |  |
| 438 | 0.0297724 | 0.547112 | 0.707171 | 0.198169 | 3.56852 |  |
| 439 | 0.002401 | 0.0820625 | 0.28506 | 0.153508 | 1.85697 |  |
| 440 | 2.13497 | 19.7464 | 6.1901 | 1.27058 | 4.87188 |  |
| 441 | 0.267471 | 2.42075 | 1.40679 | 0.535805 | 2.62557 |  |
| 442 | 0.0081634 | 0.198309 | 0.439976 | 0.178602 | 2.46345 |  |
| 443 | 1.28502 | 8.49972 | 2.72322 | 0.85221 | 3.19547 |  |
| 444 | 0.852355 | 8.38855 | 2.71086 | 0.822859 | 3.29444 |  |
| 445 | 1.00794 | 7.85287 | 2.89137 | 0.863144 | 3.34982 |  |
| 446 | 0.133015 | 1.71035 | 1.34002 | 0.336804 | 3.97863 |  |
| 447 | 0.38368 | 3.89881 | 1.65252 | 0.737105 | 2.2419 |  |
| 448 | 0.52726 | 4.86203 | 2.01779 | 0.761446 | 2.64994 |  |
| 449 | 0.0134456 | 0.322263 | 0.71243 | 0.138635 | 5.13888 |  |
| 450 | 0.0062426 | 0.183748 | 0.498648 | 0.128851 | 3.86995 |  |
| 451 | 0.0028812 | 0.092253 | 0.318123 | 0.109284 | 2.91097 |  |
| 452 | 0.0134456 | 0.310902 | 0.63163 | 0.138635 | 4.55606 |  |
| 453 | 0.088837 | 1.13843 | 0.870981 | 0.584542 | 1.49002 |  |
| 454 | 0.0019208 | 0.0603798 | 0.228969 | 0.140504 | 1.62963 |  |
| 455 | 0.0115248 | 0.293591 | 0.710671 | 0.138635 | 5.1262 |  |
| 456 | 0.640107 | 4.25782 | 1.76169 | 0.704623 | 2.50019 |  |
| 457 | 0.062426 | 0.985264 | 1.02403 | 0.327852 | 3.12344 |  |
| 458 | 0.0057624 | 0.191035 | 0.785273 | 0.228563 | 3.43569 |  |
| 459 | 0.0057624 | 0.178854 | 0.586555 | 0.182203 | 3.21924 |  |
| 460 | 0.266991 | 3.21983 | 1.80955 | 0.511306 | 3.53908 |  |
| 461 | 0.0009604 | 0.032799 | 0.109284 | 0.143766 | 0.760151 |  |
| 462 | 0.14358 | 1.60559 | 1.21368 | 0.557036 | 2.17883 |  |
| 463 | 0.0989212 | 1.28644 | 1.0986 | 0.480038 | 2.28856 |  |
| 464 | 0.0364952 | 0.781861 | 1.04741 | 0.187553 | 5.5846 |  |
| 465 | 0.212248 | 3.23637 | 2.30836 | 0.584723 | 3.94778 |  |
| 466 | 0.201204 | 2.48482 | 1.59697 | 0.677185 | 2.35825 |  |
| 467 | 0.0110446 | 0.260023 | 0.534875 | 0.178602 | 2.99479 |  |
| 468 | 0.0201684 | 0.394734 | 0.62759 | 0.188385 | 3.33142 |  |
| 469 | 0.19256 | 2.08908 | 1.5791 | 0.357203 | 4.42073 |  |
| 470 | 0.0052822 | 0.142616 | 0.364014 | 0.128852 | 2.82506 |  |
| 471 | 0.0917182 | 1.2263 | 1.11326 | 0.497654 | 2.23701 |  |
| 472 | 0.009604 | 0.249092 | 0.653298 | 0.128851 | 5.07016 |  |
| 473 | 0.002401 | 0.0769832 | 0.304721 | 0.143666 | 2.12103 |  |
| 474 | 0.235778 | 2.76651 | 1.70268 | 0.816783 | 2.08461 |  |
| 475 | 0.0019208 | 0.0603798 | 0.228973 | 0.140505 | 1.62965 |  |
| 476 | 0.0019208 | 0.0603798 | 0.22897 | 0.140504 | 1.62963 |  |
| 477 | 0.009604 | 0.246622 | 0.532646 | 0.178602 | 2.98231 |  |
| 478 | 0.0038416 | 0.119834 | 0.40915 | 0.138635 | 2.95127 |  |
| 479 | 0.0321734 | 0.64619 | 0.919649 | 0.22752 | 4.04205 |  |
| 480 | 0.0009604 | 0.032799 | 0.109284 | 0.143766 | 0.760153 |  |
| 481 | 0.0019208 | 0.0603798 | 0.228973 | 0.140504 | 1.62965 |  |
| 482 | 0.0134456 | 0.328405 | 0.746355 | 0.167986 | 4.44296 |  |
| 483 | 0.0225694 | 0.478086 | 0.981145 | 0.168818 | 5.81185 |  |
| 484 | 0.0153664 | 0.332654 | 0.531272 | 0.168818 | 3.14701 |  |
| 485 | 0.0220892 | 0.41344 | 0.563762 | 0.355384 | 1.58635 |  |
| 486 | 0.0091238 | 0.22076 | 0.436006 | 0.168818 | 2.5827 |  |
| 487 | 0.014406 | 0.299976 | 0.521537 | 0.208784 | 2.49797 |  |
| 488 | 0.0970004 | 1.17514 | 1.01843 | 0.419283 | 2.42899 |  |
| 489 | 0.0009604 | 0.032799 | 0.109284 | 0.143766 | 0.760154 |  |
| 490 | 0.0172872 | 0.433841 | 1.17296 | 0.230724 | 5.08382 |  |
| 491 | 0.0004802 | 0.019606 | 0.059534 | 0.059534 | 1 |  |
| 492 | 0.004802 | 0.140749 | 0.361977 | 0.128852 | 2.80925 |  |
| 493 | 0.0345744 | 0.756784 | 1.22177 | 0.207121 | 5.89881 |  |
| 494 | 0.162308 | 3.22235 | 2.9615 | 0.39301 | 7.53542 |  |
| 495 | 0.0019208 | 0.0603798 | 0.219567 | 0.127083 | 1.72774 |  |
| 496 | 0.0028812 | 0.0901078 | 0.361977 | 0.198655 | 1.82214 |  |
| 497 | 0.0105644 | 0.271454 | 0.717724 | 0.185013 | 3.87932 |  |
| 498 | 0.012005 | 0.261131 | 0.491929 | 0.178601 | 2.75434 |  |
| 499 | 0.0249704 | 0.470407 | 0.705919 | 0.207952 | 3.39462 |  |
| 500 | 0.002401 | 0.0758411 | 0.288523 | 0.109284 | 2.64012 |  |
| 501 | 0.0139258 | 0.294749 | 0.511166 | 0.188385 | 2.71341 |  |
| 502 | 0.0009604 | 0.0381041 | 0.208247 | 0.092506 | 2.25117 |  |
| 503 | 0.0052822 | 0.150728 | 0.403654 | 0.159034 | 2.53816 |  |
| 504 | 0.0019208 | 0.0662077 | 0.279906 | 0.145524 | 1.92343 |  |
| 505 | 0.0062426 | 0.181182 | 0.529755 | 0.138635 | 3.82122 |  |
| 506 | 0.0643468 | 1.14098 | 1.55126 | 0.218568 | 7.09739 |  |
| 507 | 0.007203 | 0.182448 | 0.446096 | 0.168818 | 2.64247 |  |
| 508 | 0.0038416 | 0.114345 | 0.355335 | 0.191371 | 1.85679 |  |
| 509 | 0.0076832 | 0.218798 | 0.696398 | 0.148419 | 4.69212 |  |
| 510 | 0.963281 | 8.77299 | 2.94466 | 1.18205 | 2.49115 |  |
| 511 | 0.074431 | 0.979356 | 1.01987 | 0.29767 | 3.42618 |  |
| 512 | 0.19208 | 3.36098 | 2.49115 | 0.266655 | 9.34221 |  |
| 513 | 0.0139258 | 0.309593 | 0.520068 | 0.178602 | 2.91189 |  |
| 514 | 0.0009604 | 0.032799 | 0.109284 | 0.143766 | 0.760151 |  |
| 515 | 0.0009604 | 0.0381041 | 0.208247 | 0.092506 | 2.25118 |  |
| 516 | 0.0758716 | 1.28565 | 1.0595 | 0.52704 | 2.01028 |  |
| 517 | 0.0148862 | 0.321764 | 0.603897 | 0.178602 | 3.38125 |  |
| 518 | 0.0278516 | 0.540222 | 0.794427 | 0.207953 | 3.82023 |  |
| 519 | 0.338061 | 4.31031 | 2.6898 | 0.346588 | 7.7608 |  |
| 520 | 0.0009604 | 0.032799 | 0.109284 | 0.143766 | 0.760153 |  |
| 521 | 0.0043218 | 0.138873 | 0.426044 | 0.1393 | 3.05847 |  |
| 522 | 0.0076832 | 0.188302 | 0.409153 | 0.178602 | 2.29087 |  |
| 523 | 0.0004802 | 0.019606 | 0.059534 | 0.059534 | 1 |  |
| 524 | 0.0494606 | 1.00332 | 1.17656 | 0.197337 | 5.96218 |  |
| 525 | 0.002401 | 0.0758411 | 0.28506 | 0.119068 | 2.39409 |  |
| 526 | 0.0004802 | 0.019606 | 0.059534 | 0.059534 | 1 |  |
| 527 | 0.002401 | 0.0758411 | 0.288521 | 0.109284 | 2.6401 |  |
| 528 | 0.0004802 | 0.019606 | 0.059534 | 0.059534 | 1 |  |
| 529 | 0.0019208 | 0.0728226 | 0.279906 | 0.069318 | 4.03802 |  |
| 530 | 0.021609 | 0.426677 | 0.718349 | 0.218568 | 3.28661 |  |
| 531 | 0.0115248 | 0.265485 | 0.520028 | 0.178602 | 2.91166 |  |
| 532 | 0.610814 | 6.36265 | 2.7569 | 0.712743 | 3.86801 |  |
| 533 | 0.127733 | 1.42646 | 1.30997 | 0.337636 | 3.87982 |  |
| 534 | 0.0153664 | 0.372364 | 0.740036 | 0.168818 | 4.38364 |  |
| 535 | 8.34011 | 44.9033 | 6.93547 | 1.24043 | 5.59119 |  |
| 536 | 0.0950796 | 1.53507 | 1.34954 | 0.266655 | 5.06099 |  |
| 537 | 0.268912 | 2.93342 | 1.55121 | 0.745421 | 2.08098 |  |
| 538 | 0.0153664 | 0.303934 | 0.537554 | 0.270171 | 1.98968 |  |
| 539 | 0.0009604 | 0.032799 | 0.109284 | 0.143766 | 0.760154 |  |
| 540 | 0.210808 | 2.37026 | 1.49452 | 0.550204 | 2.71629 |  |
| 541 | 13.0014 | 70.5391 | 7.52603 | 2.02997 | 3.70745 |  |
| 542 | 0.0177674 | 0.360364 | 0.576742 | 0.265867 | 2.16928 |  |
| 543 | 0.50469 | 4.39953 | 2.06398 | 0.636137 | 3.24456 |  |
| 544 | 0.423536 | 4.1137 | 2.18479 | 0.536671 | 4.07101 |  |
| 545 | 0.325095 | 3.07665 | 1.94786 | 0.602694 | 3.23193 |  |
| 546 | 0.133496 | 1.64222 | 1.38117 | 0.426396 | 3.23918 |  |
| 547 | 3.62839 | 21.6197 | 4.32865 | 1.54237 | 2.80649 |  |
| 548 | 0.079233 | 1.03678 | 1.09241 | 0.36581 | 2.98626 |  |
| 549 | 0.0091238 | 0.236395 | 0.511807 | 0.159034 | 3.21821 |  |
| 550 | 0.0057624 | 0.170703 | 0.602091 | 0.158759 | 3.79248 |  |
| 551 | 0.004802 | 0.134317 | 0.352348 | 0.159034 | 2.21555 |  |
| 552 | 0.266031 | 3.09206 | 1.5625 | 0.615738 | 2.53761 |  |
| 553 | 0.0038416 | 0.115541 | 0.413385 | 0.141783 | 2.91561 |  |
| 554 | 0.0638666 | 0.801426 | 0.731514 | 0.367819 | 1.98879 |  |
| 555 | 4.28338 | 29.1211 | 5.95369 | 0.870276 | 6.84114 |  |
| 556 | 1.06508 | 7.13302 | 3.38511 | 0.835191 | 4.0531 |  |
| 557 | 0.172872 | 1.79928 | 1.11624 | 0.456704 | 2.44412 |  |
| 558 | 0.0062426 | 0.191155 | 0.603897 | 0.18629 | 3.2417 |  |
| 559 | 0.630022 | 4.52961 | 1.99768 | 0.644258 | 3.10075 |  |
| 560 | 0.170471 | 1.8828 | 1.24028 | 0.631994 | 1.96248 |  |
| 561 | 0.609854 | 4.69255 | 1.92463 | 0.84409 | 2.28012 |  |
| 562 | 0.067228 | 1.32056 | 1.0975 | 0.197338 | 5.56151 |  |
| 563 | 0.187758 | 2.36674 | 1.85648 | 0.460409 | 4.03223 |  |
| 564 | 0.081634 | 1.60531 | 1.29981 | 0.216905 | 5.99253 |  |
| 565 | 0.0201684 | 0.404679 | 0.601485 | 0.188385 | 3.19284 |  |
| 566 | 0.103243 | 1.4021 | 1.40387 | 0.366155 | 3.83409 |  |
| 567 | 0.0316932 | 0.541222 | 0.674682 | 0.321638 | 2.09764 |  |
| 568 | 0.0710696 | 0.949725 | 1.07275 | 0.325825 | 3.29242 |  |
| 569 | 0.0043218 | 0.12772 | 0.352348 | 0.182203 | 1.93382 |  |
| 570 | 0.713097 | 8.19877 | 3.20868 | 0.89384 | 3.58977 |  |
| 571 | 0.329417 | 4.25213 | 2.66139 | 0.832086 | 3.19845 |  |
| 572 | 0.014406 | 0.29753 | 0.51297 | 0.228352 | 2.2464 |  |
| 573 | 0.009604 | 0.216266 | 0.426044 | 0.257792 | 1.65267 |  |
| 574 | 0.426898 | 4.57084 | 2.92758 | 0.677914 | 4.31851 |  |
| 575 | 0.0321734 | 0.50774 | 0.669143 | 0.298501 | 2.24168 |  |
| 576 | 127.808 | 262.946 | 14.62 | 3.92361 | 3.72616 |  |
| 577 | 0.0201684 | 0.365687 | 0.574078 | 0.228352 | 2.514 |  |
| 578 | 0.0009604 | 0.032799 | 0.109284 | 0.143766 | 0.760155 |  |
| 579 | 0.0091238 | 0.224486 | 0.561586 | 0.228672 | 2.45586 |  |
| 580 | 0.0062426 | 0.167725 | 0.401188 | 0.183283 | 2.1889 |  |
| 581 | 0.0009604 | 0.0381041 | 0.213139 | 0.059534 | 3.58013 |  |
| 582 | 0.0038416 | 0.124311 | 0.436008 | 0.140057 | 3.11309 |  |
| 583 | 0.0379358 | 0.73153 | 1.04648 | 0.414243 | 2.52624 |  |
| 584 | 0.016807 | 0.367833 | 0.713883 | 0.330419 | 2.16054 |  |
| 585 | 0.002401 | 0.0758411 | 0.286624 | 0.136812 | 2.09502 |  |
| 586 | 0.014406 | 0.304347 | 0.567294 | 0.178602 | 3.17631 |  |
| 587 | 1.50495 | 12.3624 | 4.73122 | 1.05454 | 4.48654 |  |
| 588 | 0.0028812 | 0.0929952 | 0.33734 | 0.176573 | 1.91048 |  |
| 589 | 0.154624 | 1.60412 | 1.1333 | 0.426521 | 2.65707 |  |
| 590 | 0.0062426 | 0.157222 | 0.374717 | 0.159034 | 2.3562 |  |
| 591 | 0.0028812 | 0.0901946 | 0.293121 | 0.109284 | 2.68219 |  |
| 592 | 0.0633864 | 1.14407 | 1.59893 | 0.237303 | 6.73793 |  |
| 593 | 0.112367 | 1.56035 | 1.23602 | 0.450754 | 2.74211 |  |
| 594 | 0.0153664 | 0.347879 | 0.760915 | 0.260866 | 2.91688 |  |
| 595 | 0.173352 | 1.98552 | 1.57891 | 0.536645 | 2.94219 |  |
| 596 | 0.0009604 | 0.032799 | 0.109284 | 0.143765 | 0.760158 |  |
| 597 | 0.0038416 | 0.114862 | 0.318127 | 0.119068 | 2.67182 |  |
| 598 | 0.009604 | 0.230575 | 0.495707 | 0.168818 | 2.93634 |  |
| 599 | 0.0057624 | 0.164561 | 0.508643 | 0.119068 | 4.27188 |  |
| 600 | 0.0052822 | 0.140756 | 0.36401 | 0.183283 | 1.98606 |  |
| 601 | 0.002401 | 0.0758411 | 0.293119 | 0.119068 | 2.46178 |  |
| 602 | 0.0014406 | 0.0560173 | 0.279906 | 0.069318 | 4.03802 |  |
| 603 | 0.0019208 | 0.0603798 | 0.22897 | 0.140506 | 1.62961 |  |
| 604 | 0.0422576 | 0.668264 | 0.724944 | 0.257703 | 2.8131 |  |
| 605 | 0.002401 | 0.0820625 | 0.286624 | 0.137894 | 2.07858 |  |
| 606 | 0.014406 | 0.330194 | 0.576739 | 0.138635 | 4.16012 |  |
| 607 | 0.0019208 | 0.0603798 | 0.228973 | 0.140502 | 1.62968 |  |
| 608 | 0.0009604 | 0.032799 | 0.109284 | 0.143766 | 0.760153 |  |
| 609 | 0.0019208 | 0.0747069 | 0.409149 | 0.146834 | 2.78647 |  |
| 610 | 0.0134456 | 0.330375 | 0.81219 | 0.234724 | 3.4602 |  |
| 611 | 0.0427378 | 0.69492 | 0.786101 | 0.247919 | 3.1708 |  |
| 612 | 0.0052822 | 0.144466 | 0.359153 | 0.168818 | 2.12746 |  |
| 613 | 0.0388962 | 0.659905 | 0.922731 | 0.30373 | 3.038 |  |
| 614 | 0.232417 | 3.16538 | 3.28737 | 0.585555 | 5.61411 |  |
| 615 | 0.0374556 | 0.571989 | 0.653295 | 0.268318 | 2.43477 |  |
| 616 | 0.009604 | 0.229442 | 0.497696 | 0.138635 | 3.58997 |  |
| 617 | 0.0038416 | 0.11624 | 0.360254 | 0.119068 | 3.02562 |  |
| 618 | 0.0028812 | 0.0848895 | 0.288523 | 0.119068 | 2.42319 |  |
| 619 | 0.0326536 | 0.693795 | 0.906208 | 0.187553 | 4.83173 |  |
| 620 | 0.0033614 | 0.0977417 | 0.293123 | 0.119068 | 2.46182 |  |
| 621 | 0.497967 | 5.55817 | 2.64674 | 0.853042 | 3.10271 |  |
| 622 | 0.0014406 | 0.0497959 | 0.233953 | 0.135086 | 1.73187 |  |
| 623 | 0.076832 | 1.1119 | 1.00098 | 0.317237 | 3.15532 |  |
| 624 | 0.139738 | 1.64777 | 1.29125 | 0.493441 | 2.61683 |  |
| 625 | 0.0057624 | 0.16149 | 0.498648 | 0.128852 | 3.86994 |  |
| 626 | 0.07203 | 1.21368 | 1.27028 | 0.387386 | 3.2791 |  |
| 627 | 0.0355348 | 0.744445 | 1.2707 | 0.208784 | 6.0862 |  |
| 628 | 0.0533022 | 1.05413 | 1.45235 | 0.313832 | 4.62778 |  |
| 629 | 0.0110446 | 0.251709 | 0.499396 | 0.188385 | 2.65092 |  |
| 630 | 0.324135 | 3.42048 | 2.3441 | 0.436304 | 5.37262 |  |
| 631 | 0.0043218 | 0.123944 | 0.361977 | 0.17878 | 2.02471 |  |
| 632 | 0.0009604 | 0.032799 | 0.109284 | 0.143766 | 0.760153 |  |
| 633 | 0.260749 | 3.24918 | 2.21533 | 0.436304 | 5.07748 |  |
| 634 | 0.326056 | 4.11403 | 2.2 | 0.463992 | 4.74146 |  |
| 635 | 0.0009604 | 0.0381041 | 0.208247 | 0.092508 | 2.25113 |  |
| 636 | 0.002401 | 0.0826196 | 0.308396 | 0.119068 | 2.59009 |  |
| 637 | 0.0019208 | 0.0662077 | 0.275415 | 0.161204 | 1.70849 |  |
| 638 | 0.002401 | 0.0820625 | 0.288523 | 0.137895 | 2.09234 |  |
| 639 | 0.0797132 | 1.61217 | 2.04097 | 0.28539 | 7.15151 |  |
| 640 | 0.0691488 | 1.45799 | 2.0154 | 0.247088 | 8.1566 |  |
| 641 | 0.0105644 | 0.248734 | 0.498648 | 0.128852 | 3.86994 |  |
| 642 | 0.0009604 | 0.032799 | 0.109284 | 0.143766 | 0.760153 |  |
| 643 | 0.225214 | 3.2306 | 1.93816 | 0.557036 | 3.47941 |  |
| 644 | 0.0907578 | 1.31689 | 1.17034 | 0.267487 | 4.37534 |  |
| 645 | 0.247303 | 2.65453 | 1.59928 | 0.396338 | 4.03514 |  |
| 646 | 0.0446586 | 0.722605 | 0.811133 | 0.323987 | 2.5036 |  |
| 647 | 0.0009604 | 0.032799 | 0.109284 | 0.143766 | 0.760153 |  |
| 648 | 0.0009604 | 0.032799 | 0.109284 | 0.143766 | 0.760155 |  |
| 649 | 0.0115248 | 0.274899 | 0.576743 | 0.178602 | 3.22921 |  |
| 650 | 0.0921984 | 1.21556 | 1.21096 | 0.308285 | 3.92806 |  |
| 651 | 1.06844 | 7.75418 | 3.1241 | 0.634473 | 4.92393 |  |
| 652 | 0.0662676 | 1.00232 | 0.938082 | 0.247919 | 3.78382 |  |
| 653 | 0.0009604 | 0.032799 | 0.109284 | 0.143764 | 0.760163 |  |
| 654 | 0.0427378 | 0.712993 | 0.782243 | 0.218568 | 3.57894 |  |
| 655 | 0.0009604 | 0.0381041 | 0.213139 | 0.059534 | 3.58013 |  |
| 656 | 0.0124852 | 0.280973 | 0.511803 | 0.178602 | 2.86561 |  |
| 657 | 7.34946 | 51.5202 | 5.81768 | 3.11627 | 1.86687 |  |
| 658 | 0.0038416 | 0.109399 | 0.359161 | 0.119068 | 3.01643 |  |
| 659 | 0.0921984 | 1.49836 | 1.54469 | 0.349083 | 4.42499 |  |
| 660 | 0.113807 | 1.30434 | 1.07944 | 0.54303 | 1.98782 |  |
| 661 | 0.060025 | 0.90733 | 0.995957 | 0.238136 | 4.18231 |  |
| 662 | 0.0657874 | 1.09769 | 1.18481 | 0.287054 | 4.12747 |  |
| 663 | 0.0273714 | 0.528101 | 0.760915 | 0.275789 | 2.75905 |  |
| 664 | 0.0033614 | 0.106213 | 0.355335 | 0.203219 | 1.74853 |  |
| 665 | 0.330858 | 3.88527 | 1.98545 | 0.306621 | 6.47524 |  |
| 666 | 0.038416 | 0.813178 | 1.04973 | 0.218568 | 4.80276 |  |
| 667 | 0.002401 | 0.0758411 | 0.285059 | 0.119068 | 2.3941 |  |
| 668 | 0.0158466 | 0.359737 | 0.724944 | 0.168818 | 4.29423 |  |
| 669 | 3.3494 | 25.4197 | 4.57647 | 1.48668 | 3.07831 |  |
| 670 | 0.012005 | 0.275476 | 0.574078 | 0.178602 | 3.21429 |  |
| 671 | 0.435061 | 4.0628 | 2.15637 | 0.575772 | 3.74518 |  |
| 672 | 0.0004802 | 0.019606 | 0.059534 | 0.059534 | 1 |  |
| 673 | 0.0105644 | 0.242695 | 0.520028 | 0.178602 | 2.91166 |  |
| 674 | 0.0019208 | 0.0603798 | 0.228971 | 0.140504 | 1.62964 |  |
| 675 | 0.0081634 | 0.206957 | 0.497775 | 0.168818 | 2.94859 |  |
| 676 | 0.0595448 | 1.02058 | 1.0289 | 0.22752 | 4.52223 |  |
| 677 | 0.0172872 | 0.392692 | 0.664102 | 0.168818 | 3.93383 |  |
| 678 | 0.127253 | 2.11299 | 1.63327 | 0.237303 | 6.88263 |  |
| 679 | 0.0725102 | 1.15471 | 1.15547 | 0.299333 | 3.86015 |  |
| 680 | 0.0009604 | 0.032799 | 0.109284 | 0.143766 | 0.760154 |  |
| 681 | 0.0797132 | 1.18489 | 1.22927 | 0.377603 | 3.25546 |  |
| 682 | 0.0019208 | 0.0603798 | 0.219568 | 0.127083 | 1.72775 |  |
| 683 | 0.0028812 | 0.0901946 | 0.286624 | 0.119068 | 2.40724 |  |
| 684 | 0.301566 | 3.35177 | 2.03949 | 0.426521 | 4.78169 |  |
| 685 | 0.012005 | 0.310387 | 0.787075 | 0.186742 | 4.21477 |  |
| 686 | 0.0004802 | 0.019606 | 0.059534 | 0.059534 | 1 |  |
| 687 | 0.0076832 | 0.193736 | 0.413385 | 0.168818 | 2.4487 |  |
| 688 | 0.0134456 | 0.364633 | 1.00972 | 0.148419 | 6.80316 |  |
| 689 | 0.183917 | 2.22618 | 1.84976 | 0.406954 | 4.54538 |  |
| 690 | 0.0028812 | 0.0879606 | 0.312679 | 0.153397 | 2.03837 |  |
| 691 | 0.0182476 | 0.356842 | 0.574078 | 0.208785 | 2.74962 |  |
| 692 | 0.0110446 | 0.281158 | 0.649508 | 0.168818 | 3.84739 |  |
| 693 | 0.0028812 | 0.0879606 | 0.318127 | 0.134435 | 2.36641 |  |
| 694 | 0.472997 | 4.25003 | 2.53223 | 0.466487 | 5.42829 |  |
| 695 | 0.002401 | 0.0907357 | 0.352346 | 0.079101 | 4.45437 |  |
| 696 | 0.153664 | 1.77387 | 1.23496 | 0.574087 | 2.15117 |  |
| 697 | 0.0019208 | 0.0603798 | 0.219567 | 0.127083 | 1.72774 |  |
| 698 | 0.0038416 | 0.104181 | 0.293119 | 0.119068 | 2.46178 |  |
| 699 | 0.0177674 | 0.348106 | 0.529751 | 0.188385 | 2.81206 |  |
| 700 | 0.0643468 | 0.957582 | 1.08364 | 0.268318 | 4.03863 |  |
| 701 | 0.636265 | 5.96059 | 2.23892 | 0.902792 | 2.48 |  |
| 702 | 0.0067228 | 0.183852 | 0.532645 | 0.119068 | 4.47347 |  |
| 703 | 0.133496 | 1.70559 | 1.60483 | 0.396338 | 4.04914 |  |
| 704 | 0.0537824 | 0.798549 | 0.846437 | 0.33453 | 2.53022 |  |
| 705 | 0.0057624 | 0.147982 | 0.359161 | 0.119068 | 3.01644 |  |
| 706 | 0.0523418 | 0.748233 | 0.860703 | 0.349558 | 2.46226 |  |
| 707 | 0.0182476 | 0.373758 | 0.573572 | 0.188385 | 3.04468 |  |
| 708 | 0.0057624 | 0.160894 | 0.497665 | 0.179509 | 2.77237 |  |
| 709 | 0.0427378 | 0.669293 | 0.821431 | 0.268318 | 3.0614 |  |
| 710 | 0.0806736 | 1.112 | 0.98002 | 0.27727 | 3.53453 |  |
| 711 | 0.0249704 | 0.449135 | 0.669147 | 0.248751 | 2.69003 |  |
| 712 | 0.0043218 | 0.131308 | 0.426278 | 0.184364 | 2.31215 |  |
| 713 | 0.0115248 | 0.273243 | 0.578457 | 0.148419 | 3.89746 |  |
| 714 | 0.002401 | 0.0907357 | 0.352344 | 0.079101 | 4.45434 |  |
| 715 | 0.0086436 | 0.228464 | 0.573249 | 0.200507 | 2.859 |  |
| 716 | 0.185837 | 2.13156 | 1.50306 | 0.317237 | 4.73796 |  |
| 717 | 0.0105644 | 0.261367 | 0.583341 | 0.224627 | 2.59694 |  |
| 718 | 0.0067228 | 0.169421 | 0.415141 | 0.128852 | 3.22186 |  |
| 719 | 0.007203 | 0.190204 | 0.449157 | 0.184689 | 2.43196 |  |
| 720 | 0.0388962 | 0.673267 | 0.79282 | 0.325628 | 2.43474 |  |
| 721 | 0.0028812 | 0.0901946 | 0.293123 | 0.109284 | 2.68221 |  |
| 722 | 0.0038416 | 0.134851 | 0.491903 | 0.119068 | 4.13128 |  |
| 723 | 0.0398566 | 0.723017 | 1.02044 | 0.228352 | 4.46873 |  |
| 724 | 0.0009604 | 0.032799 | 0.109284 | 0.143766 | 0.760153 |  |
| 725 | 0.0297724 | 0.62733 | 1.27547 | 0.298265 | 4.27629 |  |
| 726 | 0.0038416 | 0.114154 | 0.361978 | 0.128852 | 2.80927 |  |
| 727 | 0.004802 | 0.134902 | 0.356027 | 0.183283 | 1.9425 |  |
| 728 | 0.0028812 | 0.0913024 | 0.364014 | 0.109284 | 3.33089 |  |
| 729 | 0.0009604 | 0.032799 | 0.109284 | 0.143766 | 0.760155 |  |
| 730 | 0.0038416 | 0.104181 | 0.293119 | 0.119068 | 2.46178 |  |
| 731 | 0.002401 | 0.0820625 | 0.288519 | 0.137894 | 2.09232 |  |
| 732 | 0.0657874 | 1.1482 | 1.08437 | 0.22752 | 4.76605 |  |
| 733 | 0.0124852 | 0.309113 | 0.74004 | 0.128852 | 5.74335 |  |
| 734 | 0.0009604 | 0.0381041 | 0.208255 | 0.092506 | 2.25126 |  |
| 735 | 0.0019208 | 0.0603798 | 0.219564 | 0.127091 | 1.72761 |  |
| 736 | 0.0009604 | 0.032799 | 0.109284 | 0.143766 | 0.760153 |  |
| 737 | 0.0422576 | 0.798739 | 1.24647 | 0.248751 | 5.01094 |  |
| 738 | 0.0585844 | 1.00499 | 1.17132 | 0.267487 | 4.37897 |  |
| 739 | 0.0763518 | 1.07812 | 0.997188 | 0.257703 | 3.86953 |  |
| 740 | 0.0033614 | 0.110717 | 0.382363 | 0.169993 | 2.24929 |  |
| 741 | 0.0340942 | 0.71327 | 0.997826 | 0.188385 | 5.29673 |  |
| 742 | 0.0028812 | 0.0848895 | 0.293121 | 0.119068 | 2.4618 |  |
| 743 | 0.0206486 | 0.475523 | 0.92588 | 0.17777 | 5.2083 |  |
| 744 | 0.028812 | 0.550244 | 0.879942 | 0.178602 | 4.92684 |  |
| 745 | 0.0148862 | 0.312287 | 0.521535 | 0.178602 | 2.9201 |  |
| 746 | 0.0057624 | 0.195222 | 0.628519 | 0.159034 | 3.95209 |  |
| 747 | 0.0124852 | 0.286435 | 0.511804 | 0.168818 | 3.03169 |  |
| 748 | 0.007203 | 0.185299 | 0.449157 | 0.178602 | 2.51485 |  |
| 749 | 0.0585844 | 0.917078 | 1.20418 | 0.330787 | 3.64036 |  |
| 750 | 0.194001 | 2.86373 | 1.73884 | 0.296006 | 5.87436 |  |
| 751 | 0.019208 | 0.45611 | 0.861531 | 0.178602 | 4.82375 |  |
| 752 | 0.0100842 | 0.243988 | 0.573573 | 0.178602 | 3.21147 |  |
| 753 | 0.0182476 | 0.369465 | 0.628519 | 0.188385 | 3.33635 |  |
| 754 | 0.0220892 | 0.486665 | 0.94743 | 0.218568 | 4.33471 |  |
| 755 | 0.0465794 | 0.74653 | 0.9566 | 0.311623 | 3.06974 |  |
| 756 | 0.0533022 | 0.850692 | 1.10618 | 0.257703 | 4.29246 |  |
| 757 | 0.134456 | 2.02091 | 1.66129 | 0.345756 | 4.80482 |  |
| 758 | 0.0355348 | 0.626975 | 0.802388 | 0.257703 | 3.11361 |  |
| 759 | 0.0067228 | 0.187307 | 0.497697 | 0.119068 | 4.17994 |  |
| 760 | 0.002401 | 0.0758411 | 0.288523 | 0.137894 | 2.09235 |  |
| 761 | 0.0038416 | 0.104181 | 0.293115 | 0.119068 | 2.46174 |  |
| 762 | 134.81 | 281.169 | 12.0365 | 6.9844 | 1.72334 |  |
| 763 | 0.0134456 | 0.324086 | 0.71243 | 0.178602 | 3.98893 |  |
| 764 | 0.0009604 | 0.032799 | 0.109284 | 0.143766 | 0.760151 |  |
| 765 | 0.009604 | 0.240803 | 0.529747 | 0.148419 | 3.56927 |  |
| 766 | 0.0033614 | 0.116781 | 0.361977 | 0.1393 | 2.59855 |  |
| 767 | 0.909499 | 15.7551 | 6.30934 | 0.374276 | 16.8575 |  |
| 768 | 0.0028812 | 0.0901946 | 0.286624 | 0.119068 | 2.40724 |  |
| 769 | 0.0057624 | 0.147982 | 0.359161 | 0.119068 | 3.01644 |  |
| 770 | 0.0009604 | 0.032799 | 0.109284 | 0.143766 | 0.760151 |  |
| 771 | 0.511413 | 5.77078 | 2.95397 | 0.453376 | 6.51549 |  |
| 772 | 1.65525 | 12.0104 | 3.28547 | 0.94359 | 3.48188 |  |
| 773 | 0.0004802 | 0.019606 | 0.059534 | 0.059534 | 1 |  |
| 774 | 0.0019208 | 0.0603798 | 0.219567 | 0.127083 | 1.72774 |  |
| 775 | 0.0009604 | 0.032799 | 0.109284 | 0.143766 | 0.760155 |  |
| 776 | 0.183917 | 2.12157 | 1.42806 | 0.466488 | 3.0613 |  |
| 777 | 0.0374556 | 0.72786 | 1.00687 | 0.307344 | 3.27604 |  |
| 778 | 0.0249704 | 0.633882 | 1.11887 | 0.187554 | 5.96557 |  |
| 779 | 0.004802 | 0.139127 | 0.498648 | 0.128851 | 3.86995 |  |
| 780 | 0.009604 | 0.257381 | 0.616793 | 0.128851 | 4.78686 |  |
| 781 | 0.0038416 | 0.109399 | 0.312677 | 0.128851 | 2.42665 |  |
| 782 | 0.0038416 | 0.104181 | 0.293123 | 0.119068 | 2.46182 |  |
| 783 | 0.195922 | 2.7748 | 1.70207 | 0.396338 | 4.29449 |  |
| 784 | 5.72879 | 56.9584 | 7.83453 | 1.07474 | 7.28971 |  |
| 785 | 0.055223 | 0.958837 | 1.36937 | 0.403694 | 3.39209 |  |
| 786 | 0.0211288 | 0.391592 | 0.605007 | 0.274061 | 2.20757 |  |
| 787 | 0.019208 | 0.36272 | 0.566551 | 0.258535 | 2.19139 |  |
| 788 | 1.4963 | 9.60042 | 2.96949 | 0.856369 | 3.46754 |  |
| 789 | 0.209847 | 2.85394 | 1.67852 | 0.345756 | 4.85464 |  |
| 790 | 0.0028812 | 0.0879606 | 0.318131 | 0.134435 | 2.36644 |  |
| 791 | 0.009604 | 0.257381 | 0.705913 | 0.189225 | 3.73054 |  |
| 792 | 0.0004802 | 0.019606 | 0.059534 | 0.059534 | 1 |  |
| 793 | 0.110446 | 1.52611 | 1.24756 | 0.437137 | 2.85393 |  |
| 794 | 0.0182476 | 0.363079 | 0.63524 | 0.188385 | 3.37202 |  |
| 795 | 0.471556 | 4.50495 | 2.11219 | 0.704623 | 2.99762 |  |
| 796 | 0.0369754 | 0.708821 | 1.03334 | 0.218568 | 4.72777 |  |
| 797 | 0.016807 | 0.367241 | 0.636255 | 0.23162 | 2.74697 |  |
| 798 | 0.0955598 | 1.13226 | 0.971911 | 0.455183 | 2.13521 |  |
| 799 | 0.0235298 | 0.515529 | 0.676424 | 0.218568 | 3.0948 |  |
| 800 | 57.5236 | 228.584 | 16.6233 | 3.36669 | 4.93759 |  |
| 801 | 160.542 | 469.357 | 17.1506 | 6.27807 | 2.73184 |  |
| 802 | 0.181035 | 2.25114 | 1.61992 | 0.296838 | 5.45726 |  |
| 803 | 0.106604 | 1.47557 | 1.33029 | 0.257703 | 5.1621 |  |
| 804 | 0.161347 | 2.33378 | 1.5179 | 0.316405 | 4.79733 |  |
| 805 | 0.188719 | 2.31242 | 2.00054 | 0.387386 | 5.16419 |  |
| 806 | 0.0158466 | 0.350392 | 0.699154 | 0.168818 | 4.14147 |  |
| 807 | 0.448987 | 4.99767 | 2.15568 | 0.426521 | 5.05411 |  |
| 808 | 0.038416 | 0.70718 | 1.01645 | 0.283155 | 3.58974 |  |
| 809 | 0.0105644 | 0.258593 | 0.517087 | 0.178602 | 2.8952 |  |
| 810 | 0.707815 | 7.33626 | 2.87021 | 0.705455 | 4.06859 |  |
| 811 | 0.088837 | 1.61313 | 1.89754 | 0.237304 | 7.99624 |  |
| 812 | 0.166629 | 1.96953 | 1.41309 | 0.367819 | 3.84182 |  |
| 813 | 0.0105644 | 0.249405 | 0.435763 | 0.168818 | 2.58126 |  |
| 814 | 0.530141 | 6.87423 | 2.89234 | 0.684224 | 4.22719 |  |
| 815 | 0.0019208 | 0.0603798 | 0.228973 | 0.140504 | 1.62965 |  |
| 816 | 0.0009604 | 0.0381041 | 0.213139 | 0.059534 | 3.58013 |  |
| 817 | 1.34264 | 9.11778 | 3.86209 | 0.813907 | 4.74512 |  |
| 818 | 0.0009604 | 0.0381041 | 0.208249 | 0.092506 | 2.25121 |  |
| 819 | 0.0052822 | 0.148304 | 0.361977 | 0.183283 | 1.97496 |  |
| 820 | 0.0489804 | 0.830156 | 0.929599 | 0.22752 | 4.08578 |  |
| 821 | 0.0163268 | 0.342243 | 0.57693 | 0.257883 | 2.23718 |  |
| 822 | 0.0028812 | 0.0848895 | 0.288523 | 0.119068 | 2.42318 |  |
| 823 | 0.0230496 | 0.546487 | 1.17132 | 0.148419 | 7.89196 |  |
| 824 | 0.091238 | 1.25614 | 1.0383 | 0.27727 | 3.74474 |  |
| 825 | 0.07203 | 1.22925 | 1.1286 | 0.327852 | 3.4424 |  |
| 826 | 0.028812 | 0.560986 | 0.721821 | 0.346927 | 2.08062 |  |
| 827 | 0.0076832 | 0.193213 | 0.416547 | 0.168818 | 2.46743 |  |
| 828 | 0.269392 | 3.26729 | 2.16741 | 0.630183 | 3.43934 |  |
| 829 | 0.0067228 | 0.18346 | 0.435763 | 0.207248 | 2.10261 |  |
| 830 | 0.0038416 | 0.106982 | 0.309435 | 0.182203 | 1.6983 |  |
| 831 | 0.0158466 | 0.311538 | 0.511166 | 0.218568 | 2.3387 |  |
| 832 | 0.0196882 | 0.452662 | 0.724944 | 0.138635 | 5.22915 |  |
| 833 | 0.002401 | 0.0758411 | 0.286624 | 0.136814 | 2.09499 |  |
| 834 | 0.0067228 | 0.16045 | 0.374716 | 0.168818 | 2.21964 |  |
| 835 | 0.004802 | 0.143122 | 0.508639 | 0.149138 | 3.41053 |  |
| 836 | 0.004802 | 0.13698 | 0.445903 | 0.119068 | 3.74495 |  |
| 837 | 0.0225694 | 0.509499 | 0.915968 | 0.138635 | 6.60704 |  |
| 838 | 0.0014406 | 0.0497959 | 0.233949 | 0.135086 | 1.73186 |  |
| 839 | 0.0009604 | 0.032799 | 0.109284 | 0.143766 | 0.760153 |  |
| 840 | 0.0009604 | 0.032799 | 0.109284 | 0.143766 | 0.760153 |  |
| 841 | 0.0028812 | 0.0879606 | 0.318127 | 0.134435 | 2.36641 |  |
| 842 | 0.0067228 | 0.172492 | 0.445903 | 0.119068 | 3.74495 |  |
| 843 | 1.31623 | 9.23129 | 2.97747 | 0.642593 | 4.63352 |  |
| 844 | 0.346704 | 3.93307 | 2.31925 | 0.707159 | 3.27967 |  |
| 845 | 0.0163268 | 0.387502 | 0.796001 | 0.167987 | 4.73848 |  |
| 846 | 0.0139258 | 0.30379 | 0.511166 | 0.178601 | 2.86205 |  |
| 847 | 0.009604 | 0.237731 | 0.576743 | 0.128852 | 4.47602 |  |
| 848 | 13.8903 | 50.2791 | 6.15095 | 1.66612 | 3.69179 |  |
| 849 | 0.0172872 | 0.343673 | 0.597669 | 0.299644 | 1.99459 |  |
| 850 | 0.0009604 | 0.0381041 | 0.213139 | 0.059534 | 3.58011 |  |
| 851 | 0.0057624 | 0.148331 | 0.366904 | 0.159034 | 2.30707 |  |
| 852 | 0.0019208 | 0.0662077 | 0.275416 | 0.161203 | 1.70851 |  |
| 853 | 0.0009604 | 0.032799 | 0.109284 | 0.143766 | 0.760153 |  |
| 854 | 0.0657874 | 0.99901 | 0.997296 | 0.307453 | 3.24373 |  |
| 855 | 0.113807 | 1.67475 | 1.43465 | 0.407233 | 3.52292 |  |
| 856 | 0.0172872 | 0.381926 | 0.635242 | 0.138635 | 4.58211 |  |
| 857 | 0.0249704 | 0.521348 | 0.901035 | 0.335747 | 2.68367 |  |
| 858 | 0.268912 | 3.66226 | 2.21695 | 0.700431 | 3.16513 |  |
| 859 | 0.0038416 | 0.116397 | 0.361977 | 0.138219 | 2.61887 |  |
| 860 | 0.0019208 | 0.0603798 | 0.219564 | 0.127086 | 1.72768 |  |
| 861 | 0.0235298 | 0.446665 | 0.723954 | 0.271576 | 2.66575 |  |
| 862 | 0.0004802 | 0.019606 | 0.059534 | 0.059534 | 1 |  |
| 863 | 0.0182476 | 0.35231 | 0.567294 | 0.218568 | 2.5955 |  |
| 864 | 0.0758716 | 1.57308 | 1.98303 | 0.265823 | 7.45996 |  |
| 865 | 0.0086436 | 0.218544 | 0.498648 | 0.168818 | 2.95376 |  |
| 866 | 0.0869162 | 1.15203 | 1.00268 | 0.297669 | 3.36842 |  |
| 867 | 53.7838 | 121.364 | 8.62234 | 3.43673 | 2.50888 |  |
| 868 | 0.0019208 | 0.0603798 | 0.228971 | 0.140506 | 1.62962 |  |
| 869 | 0.0043218 | 0.12772 | 0.356027 | 0.183281 | 1.94252 |  |
| 870 | 0.0004802 | 0.019606 | 0.059534 | 0.059534 | 1 |  |
| 871 | 0.0100842 | 0.229258 | 0.445903 | 0.178602 | 2.49663 |  |
| 872 | 0.0235298 | 0.460925 | 0.740036 | 0.208785 | 3.54449 |  |
| 873 | 0.0605052 | 0.828328 | 0.821434 | 0.278102 | 2.95372 |  |
| 874 | 0.057624 | 1.00233 | 1.33691 | 0.45565 | 2.93408 |  |
| 875 | 0.0076832 | 0.19733 | 0.449157 | 0.204052 | 2.20118 |  |
| 876 | 0.0115248 | 0.285948 | 0.631381 | 0.148419 | 4.25405 |  |
| 877 | 0.0052822 | 0.142477 | 0.361977 | 0.182203 | 1.98667 |  |
| 878 | 0.104684 | 1.59131 | 1.44053 | 0.406693 | 3.54205 |  |
| 879 | 0.0004802 | 0.019606 | 0.059534 | 0.059534 | 1 |  |
| 880 | 0.0086436 | 0.197384 | 0.42321 | 0.227592 | 1.85952 |  |
| 881 | 0.0028812 | 0.0901946 | 0.293123 | 0.109284 | 2.68221 |  |
| 882 | 0.0172872 | 0.365084 | 0.637739 | 0.178602 | 3.57074 |  |
| 883 | 0.0124852 | 0.28134 | 0.498912 | 0.168818 | 2.95532 |  |
| 884 | 0.021609 | 0.424174 | 0.751229 | 0.208785 | 3.59811 |  |
| 885 | 0.0009604 | 0.032799 | 0.109284 | 0.143766 | 0.760153 |  |
| 886 | 0.0019208 | 0.0603798 | 0.22897 | 0.140505 | 1.62963 |  |
| 887 | 0.0009604 | 0.032799 | 0.109284 | 0.143766 | 0.760154 |  |
| 888 | 0.0009604 | 0.0381041 | 0.20825 | 0.092506 | 2.2512 |  |
| 889 | 0.0019208 | 0.0603798 | 0.22897 | 0.140504 | 1.62963 |  |
| 890 | 0.004802 | 0.128691 | 0.364012 | 0.128851 | 2.82505 |  |
| 891 | 0.0019208 | 0.0603798 | 0.228973 | 0.140504 | 1.62965 |  |
| 892 | 0.0081634 | 0.200264 | 0.445481 | 0.159034 | 2.80116 |  |
| 893 | 0.0009604 | 0.032799 | 0.109284 | 0.143766 | 0.760151 |  |
| 894 | 0.0009604 | 0.032799 | 0.109284 | 0.143766 | 0.760151 |  |
| 895 | 0.0211288 | 0.494396 | 1.01072 | 0.138635 | 7.29048 |  |
| 896 | 0.0014406 | 0.0560173 | 0.275415 | 0.069318 | 3.97323 |  |
| 897 | 0.0014406 | 0.0497959 | 0.233949 | 0.13509 | 1.7318 |  |
| 898 | 0.0158466 | 0.368376 | 0.713466 | 0.168818 | 4.22624 |  |
| 899 | 0.0019208 | 0.0728226 | 0.279905 | 0.069318 | 4.038 |  |
| 900 | 0.0019208 | 0.0603798 | 0.219567 | 0.127085 | 1.72771 |  |
| 901 | 0.0211288 | 0.438695 | 0.757349 | 0.303312 | 2.49692 |  |
| 902 | 0.0110446 | 0.265286 | 0.559812 | 0.168818 | 3.31607 |  |
| 903 | 0.450908 | 4.81181 | 2.3257 | 0.970854 | 2.39552 |  |
| 904 | 0.057624 | 1.00791 | 1.40467 | 0.248751 | 5.64687 |  |
| 905 | 0.0009604 | 0.032799 | 0.109284 | 0.143766 | 0.76015 |  |
| 906 | 0.069629 | 1.41077 | 2.07144 | 0.238136 | 8.69857 |  |
| 907 | 0.0028812 | 0.0848895 | 0.293119 | 0.119068 | 2.46179 |  |
| 908 | 0.0249704 | 0.547917 | 0.894156 | 0.167986 | 5.32279 |  |
| 909 | 0.0086436 | 0.215848 | 0.452414 | 0.209409 | 2.16043 |  |
| 910 | 0.0725102 | 1.17274 | 1.12496 | 0.237304 | 4.74058 |  |
| 911 | 0.0307328 | 0.563221 | 0.901706 | 0.228352 | 3.94876 |  |
| 912 | 0.0009604 | 0.032799 | 0.109284 | 0.143766 | 0.760152 |  |
| 913 | 0.0081634 | 0.204348 | 0.491929 | 0.159034 | 3.09322 |  |
| 914 | 0.204565 | 2.3672 | 1.52675 | 0.646753 | 2.36065 |  |
| 915 | 0.0115248 | 0.273243 | 0.521537 | 0.128852 | 4.04758 |  |
| 916 | 0.0009604 | 0.032799 | 0.109284 | 0.143766 | 0.760153 |  |
| 917 | 0.0028812 | 0.0913024 | 0.318127 | 0.137894 | 2.30703 |  |
| 918 | 0.0038416 | 0.104181 | 0.293123 | 0.119068 | 2.46182 |  |
| 919 | 0.0825944 | 1.59627 | 1.9423 | 0.250415 | 7.75632 |  |
| 920 | 0.0038416 | 0.106982 | 0.312579 | 0.180797 | 1.7289 |  |
| 921 | 0.0609854 | 1.22788 | 1.68593 | 0.315573 | 5.34244 |  |
| 922 | 11.605 | 78.0783 | 8.29047 | 3.07473 | 2.69633 |  |
| 923 | 0.0105644 | 0.238891 | 0.511168 | 0.230077 | 2.22173 |  |
| 924 | 0.0081634 | 0.195815 | 0.413385 | 0.178602 | 2.31456 |  |
| 925 | 0.0182476 | 0.353384 | 0.523831 | 0.218568 | 2.39665 |  |
| 926 | 0.0340942 | 0.608564 | 0.723954 | 0.311409 | 2.32477 |  |
| 927 | 0.0355348 | 0.642255 | 0.847265 | 0.198169 | 4.27547 |  |
| 928 | 0.033614 | 0.712215 | 1.18766 | 0.247088 | 4.80664 |  |
| 929 | 0.012005 | 0.268853 | 0.491903 | 0.178602 | 2.75419 |  |
| 930 | 0.0268912 | 0.5066 | 0.661866 | 0.198169 | 3.33991 |  |
| 931 | 0.009604 | 0.265417 | 0.724937 | 0.178601 | 4.05897 |  |
| 932 | 0.142619 | 1.91417 | 1.5703 | 0.238136 | 6.59415 |  |
| 933 | 0.0465794 | 0.729908 | 0.943951 | 0.32226 | 2.92916 |  |
| 934 | 0.0902776 | 1.4308 | 1.26097 | 0.217737 | 5.79129 |  |
| 935 | 0.831706 | 6.306 | 2.50916 | 0.67444 | 3.72037 |  |
| 936 | 0.40769 | 3.89078 | 1.83008 | 0.705455 | 2.59419 |  |
| 937 | 0.103723 | 1.4685 | 1.46329 | 0.375939 | 3.89237 |  |
| 938 | 0.130134 | 1.73817 | 1.72619 | 0.50387 | 3.42586 |  |
| 939 | 0.004802 | 0.128691 | 0.361977 | 0.128852 | 2.80926 |  |
| 940 | 0.0681884 | 1.09666 | 1.066 | 0.307453 | 3.46721 |  |
| 941 | 0.0019208 | 0.0603798 | 0.228973 | 0.140502 | 1.62968 |  |
| 942 | 0.0105644 | 0.253952 | 0.576741 | 0.128851 | 4.47602 |  |
| 943 | 0.446106 | 4.65648 | 2.25434 | 0.753541 | 2.99165 |  |
| 944 | 0.2886 | 2.8275 | 1.61909 | 0.545589 | 2.96761 |  |
| 945 | 0.0009604 | 0.032799 | 0.109284 | 0.143766 | 0.760153 |  |
| 946 | 0.422576 | 4.03868 | 1.61069 | 0.835138 | 1.92865 |  |
| 947 | 0.0004802 | 0.019606 | 0.059534 | 0.059534 | 1 |  |
| 948 | 0.0009604 | 0.0381041 | 0.208247 | 0.092506 | 2.25117 |  |
| 949 | 0.0014406 | 0.0560173 | 0.279906 | 0.069318 | 4.03802 |  |
| 950 | 0.0057624 | 0.164131 | 0.439976 | 0.184687 | 2.38227 |  |
| 951 | 0.019208 | 0.386699 | 0.737247 | 0.208785 | 3.53114 |  |
| 952 | 0.203605 | 2.64579 | 2.28681 | 0.645089 | 3.54495 |  |
| 953 | 0.0086436 | 0.220708 | 0.521533 | 0.128852 | 4.04755 |  |
| 954 | 0.0158466 | 0.379667 | 0.657539 | 0.138635 | 4.74295 |  |
| 955 | 0.148862 | 1.95443 | 1.5809 | 0.316405 | 4.99643 |  |
| 956 | 0.0518616 | 1.04446 | 1.11949 | 0.17777 | 6.29742 |  |
| 957 | 0.002401 | 0.0758411 | 0.288523 | 0.137894 | 2.09235 |  |
| 958 | 0.893652 | 8.27886 | 3.09075 | 0.793508 | 3.89505 |  |
| 959 | 0.002401 | 0.0826196 | 0.304721 | 0.137894 | 2.20981 |  |
| 960 | 0.373596 | 3.79323 | 2.10347 | 0.690539 | 3.04612 |  |
| 961 | 0.0019208 | 0.0728226 | 0.279906 | 0.069318 | 4.03802 |  |
| 962 | 0.033614 | 0.611053 | 0.737911 | 0.198169 | 3.72365 |  |
| 963 | 0.004802 | 0.134317 | 0.356027 | 0.199354 | 1.7859 |  |
| 964 | 0.007203 | 0.184157 | 0.428908 | 0.168818 | 2.54065 |  |
| 965 | 0.0019208 | 0.0603798 | 0.228971 | 0.140504 | 1.62964 |  |
| 966 | 0.0009604 | 0.032799 | 0.109284 | 0.143765 | 0.760158 |  |
| 967 | 0.0235298 | 0.507797 | 0.924879 | 0.259954 | 3.55786 |  |
| 968 | 0.0110446 | 0.28127 | 0.664102 | 0.188385 | 3.52523 |  |
| 969 | 0.0681884 | 0.851725 | 0.672211 | 0.57185 | 1.1755 |  |
| 970 | 0.0158466 | 0.353175 | 0.833824 | 0.228352 | 3.65148 |  |
| 971 | 0.016807 | 0.345663 | 0.605007 | 0.270819 | 2.23399 |  |
| 972 | 0.0427378 | 0.81692 | 0.965628 | 0.533146 | 1.81119 |  |
| 973 | 0.0019208 | 0.0728226 | 0.279906 | 0.069318 | 4.03802 |  |
| 974 | 0.0014406 | 0.0497959 | 0.237959 | 0.119735 | 1.98738 |  |
| 975 | 0.0571438 | 0.886668 | 1.06097 | 0.352237 | 3.01209 |  |
| 976 | 0.0753914 | 0.982284 | 1.08274 | 0.434808 | 2.49015 |  |
| 977 | 0.107085 | 1.22993 | 1.06305 | 0.48242 | 2.20357 |  |
| 978 | 0.0537824 | 0.897238 | 1.3699 | 0.278102 | 4.92587 |  |
| 979 | 0.0086436 | 0.225637 | 0.508643 | 0.168818 | 3.01297 |  |
| 980 | 0.012005 | 0.278487 | 0.578457 | 0.231806 | 2.49544 |  |
| 981 | 0.02401 | 0.508673 | 0.797268 | 0.25014 | 3.18729 |  |
| 982 | 0.0485002 | 0.728878 | 0.802388 | 0.257703 | 3.11361 |  |
| 983 | 0.0537824 | 0.908578 | 1.19812 | 0.267487 | 4.47917 |  |
| 984 | 0.0081634 | 0.195735 | 0.458782 | 0.229324 | 2.00058 |  |
| 985 | 0.237219 | 2.43434 | 1.70188 | 0.546421 | 3.1146 |  |
| 986 | 0.0998816 | 1.57835 | 1.52045 | 0.297669 | 5.10786 |  |
| 987 | 0.0148862 | 0.313197 | 0.579781 | 0.208785 | 2.77694 |  |
| 988 | 0.0062426 | 0.159839 | 0.361977 | 0.168818 | 2.14419 |  |
| 989 | 0.0081634 | 0.209392 | 0.55083 | 0.138635 | 3.97323 |  |
| 990 | 0.0019208 | 0.0662077 | 0.275415 | 0.161203 | 1.7085 |  |
| 991 | 0.0187278 | 0.385118 | 0.716469 | 0.188385 | 3.80321 |  |
| 992 | 0.0009604 | 0.032799 | 0.109284 | 0.143766 | 0.760153 |  |
| 993 | 0.0019208 | 0.0728226 | 0.279906 | 0.069318 | 4.03802 |  |
| 994 | 0.142619 | 2.03899 | 1.73594 | 0.316405 | 5.48645 |  |
| 995 | 0.009604 | 0.244893 | 0.567298 | 0.159034 | 3.56714 |  |
| 996 | 0.0417774 | 0.743756 | 1.10156 | 0.31323 | 3.51679 |  |
| 997 | 0.0374556 | 0.618731 | 0.791873 | 0.247919 | 3.19408 |  |
| 998 | 0.0028812 | 0.0879606 | 0.312677 | 0.153397 | 2.03835 |  |
| 999 | 0.121971 | 1.53405 | 1.31989 | 0.419581 | 3.14574 |  |
| 1000 | 0.0019208 | 0.0603798 | 0.219567 | 0.127083 | 1.72774 |  |
| 1001 | 0.0715498 | 0.985107 | 1.01125 | 0.362167 | 2.79221 |  |
| 1002 | 0.012005 | 0.273583 | 0.55083 | 0.178602 | 3.08413 |  |
| 1003 | 0.0004802 | 0.019606 | 0.059534 | 0.059534 | 1 |  |
| 1004 | 0.009604 | 0.222818 | 0.435763 | 0.178602 | 2.43986 |  |
| 1005 | 0.0148862 | 0.314347 | 0.546791 | 0.188385 | 2.90252 |  |
| 1006 | 0.441784 | 4.56703 | 2.55102 | 0.734806 | 3.47169 |  |
| 1007 | 0.0254506 | 0.530422 | 0.935274 | 0.274709 | 3.4046 |  |
| 1008 | 0.0081634 | 0.196991 | 0.428908 | 0.178602 | 2.40148 |  |
| 1009 | 0.0004802 | 0.019606 | 0.059534 | 0.059534 | 1 |  |
| 1010 | 18.056 | 73.4746 | 8.32748 | 2.9458 | 2.8269 |  |
| 1011 | 0.0057624 | 0.168669 | 0.428906 | 0.159034 | 2.69694 |  |
| 1012 | 0.0062426 | 0.16134 | 0.359161 | 0.159034 | 2.25838 |  |
| 1013 | 0.0019208 | 0.0603798 | 0.228969 | 0.140504 | 1.62962 |  |
| 1014 | 0.004802 | 0.13698 | 0.413384 | 0.119068 | 3.47184 |  |
| 1015 | 0.0609854 | 1.19906 | 1.7033 | 0.217737 | 7.82278 |  |
| 1016 | 0.009604 | 0.229442 | 0.495942 | 0.128852 | 3.84894 |  |
| 1017 | 0.673721 | 6.69508 | 2.2478 | 0.955869 | 2.35158 |  |
| 1018 | 0.892212 | 8.72108 | 2.49376 | 0.913408 | 2.73017 |  |
| 1019 | 0.009604 | 0.252163 | 0.653294 | 0.128851 | 5.07014 |  |
| 1020 | 0.0038416 | 0.109399 | 0.318127 | 0.119068 | 2.67181 |  |
| 1021 | 0.0547428 | 1.09005 | 1.03418 | 0.167986 | 6.15631 |  |
| 1022 | 12.3546 | 60.1098 | 7.39042 | 2.49673 | 2.96004 |  |
| 1023 | 0.0614656 | 0.874059 | 0.941035 | 0.34742 | 2.70864 |  |
| 1024 | 0.0110446 | 0.264527 | 0.529751 | 0.168818 | 3.138 |  |
| 1025 | 0.0201684 | 0.450237 | 0.740036 | 0.138635 | 5.33801 |  |
| 1026 | 0.0259308 | 0.538109 | 0.860684 | 0.188385 | 4.56874 |  |
| 1027 | 0.538304 | 5.67776 | 3.35282 | 0.406122 | 8.25569 |  |
| 1028 | 0.0004802 | 0.019606 | 0.059534 | 0.059534 | 1 |  |
| 1029 | 0.0187278 | 0.399219 | 0.764178 | 0.188385 | 4.05646 |  |
| 1030 | 0.0412972 | 0.690697 | 0.817037 | 0.228352 | 3.57798 |  |
| 1031 | 0.0403368 | 0.630721 | 0.755598 | 0.268319 | 2.81605 |  |
| 1032 | 0.0302526 | 0.620752 | 1.18888 | 0.27784 | 4.27901 |  |
| 1033 | 0.0182476 | 0.424916 | 0.984931 | 0.168818 | 5.83427 |  |
| 1034 | 0.0009604 | 0.032799 | 0.109284 | 0.143766 | 0.760153 |  |
| 1035 | 0.557992 | 4.97373 | 2.11864 | 0.844117 | 2.50989 |  |
| 1036 | 0.0629062 | 0.969665 | 1.10143 | 0.297669 | 3.70016 |  |
| 1037 | 0.0009604 | 0.032799 | 0.109284 | 0.143764 | 0.760163 |  |
| 1038 | 0.0009604 | 0.032799 | 0.109284 | 0.143766 | 0.760153 |  |
| 1039 | 0.0076832 | 0.195997 | 0.429407 | 0.168818 | 2.54361 |  |
| 1040 | 0.0614656 | 1.06247 | 1.38846 | 0.188386 | 7.37032 |  |
| 1041 | 0.012005 | 0.264718 | 0.491925 | 0.168818 | 2.91394 |  |
| 1042 | 0.0009604 | 0.032799 | 0.109284 | 0.143766 | 0.760151 |  |
| 1043 | 0.0009604 | 0.032799 | 0.109284 | 0.143766 | 0.760153 |  |
| 1044 | 0.0019208 | 0.0603798 | 0.22897 | 0.140504 | 1.62963 |  |
| 1045 | 0.0043218 | 0.143061 | 0.60345 | 0.15709 | 3.84142 |  |
| 1046 | 0.0004802 | 0.019606 | 0.059534 | 0.059534 | 1 |  |
| 1047 | 0.007203 | 0.190187 | 0.445903 | 0.128852 | 3.4606 |  |
| 1048 | 0.031213 | 0.653144 | 0.947426 | 0.208785 | 4.53782 |  |
| 1049 | 0.0004802 | 0.019606 | 0.059534 | 0.059534 | 1 |  |
| 1050 | 0.0033614 | 0.105656 | 0.360254 | 0.119068 | 3.02562 |  |
| 1051 | 0.0019208 | 0.0603798 | 0.219567 | 0.127085 | 1.72771 |  |
| 1052 | 0.0028812 | 0.0879606 | 0.312679 | 0.153397 | 2.03836 |  |
| 1053 | 0.007203 | 0.182273 | 0.426322 | 0.128852 | 3.30863 |  |
| 1054 | 0.028812 | 0.655169 | 1.25781 | 0.138635 | 9.07281 |  |
| 1055 | 0.0379358 | 0.738974 | 0.973669 | 0.348166 | 2.79657 |  |
| 1056 | 0.0124852 | 0.294682 | 0.630978 | 0.148419 | 4.25134 |  |
| 1057 | 0.0244902 | 0.45554 | 0.699162 | 0.218568 | 3.19883 |  |
| 1058 | 0.0115248 | 0.273243 | 0.55083 | 0.138635 | 3.97323 |  |
| 1059 | 0.940232 | 9.95462 | 3.77305 | 1.35138 | 2.79201 |  |
| 1060 | 0.675641 | 4.23587 | 1.80153 | 0.774772 | 2.32524 |  |
| 1061 | 0.0038416 | 0.114617 | 0.423218 | 0.128851 | 3.28454 |  |
| 1062 | 0.0806736 | 1.17493 | 1.17489 | 0.27727 | 4.23735 |  |
| 1063 | 0.250664 | 2.49265 | 1.47925 | 0.63274 | 2.33785 |  |
| 1064 | 0.519096 | 4.43059 | 2.47715 | 0.746253 | 3.31945 |  |
| 1065 | 4.42024 | 21.3233 | 3.71231 | 1.9169 | 1.93662 |  |
| 1066 | 87.6096 | 240.683 | 14.3682 | 4.08223 | 3.5197 |  |
| 1067 | 0.07203 | 1.08387 | 1.12363 | 0.35664 | 3.1506 |  |
| 1068 | 0.0230496 | 0.472478 | 0.71243 | 0.259956 | 2.74058 |  |
| 1069 | 0.0297724 | 0.534723 | 0.811133 | 0.303729 | 2.67058 |  |
| 1070 | 0.352947 | 2.85317 | 1.60694 | 0.639004 | 2.51476 |  |
| 1071 | 0.0235298 | 0.421547 | 0.635242 | 0.238136 | 2.66756 |  |
| 1072 | 0.0316932 | 0.525124 | 0.657543 | 0.238136 | 2.76121 |  |
| 1073 | 0.582963 | 4.51774 | 1.85054 | 0.915071 | 2.02229 |  |
| 1074 | 0.1431 | 1.92191 | 1.95547 | 0.486887 | 4.01627 |  |
| 1075 | 0.0009604 | 0.032799 | 0.109284 | 0.143766 | 0.760153 |  |
| 1076 | 0.0340942 | 0.620162 | 0.817039 | 0.188385 | 4.33706 |  |
| 1077 | 0.0259308 | 0.499647 | 0.840546 | 0.188385 | 4.46184 |  |
| 1078 | 0.002401 | 0.0758411 | 0.285057 | 0.119068 | 2.39408 |  |
| 1079 | 0.0734706 | 1.02065 | 0.827628 | 0.600124 | 1.37909 |  |
| 1080 | 0.0725102 | 0.935651 | 0.855488 | 0.390492 | 2.1908 |  |
| 1081 | 0.947915 | 8.78445 | 3.49515 | 0.755595 | 4.6257 |  |
| 1082 | 0.0052822 | 0.149029 | 0.364011 | 0.128852 | 2.82504 |  |
| 1083 | 0.130614 | 1.55117 | 1.20419 | 0.507286 | 2.37378 |  |
| 1084 | 0.0028812 | 0.092253 | 0.312677 | 0.166099 | 1.88248 |  |
| 1085 | 0.0004802 | 0.019606 | 0.059534 | 0.059534 | 1 |  |
| 1086 | 1.27973 | 9.60215 | 3.25246 | 1.26525 | 2.5706 |  |
| 1087 | 0.0014406 | 0.0497959 | 0.228971 | 0.130774 | 1.7509 |  |
| 1088 | 0.0062426 | 0.158915 | 0.37424 | 0.178602 | 2.09539 |  |
| 1089 | 0.157025 | 1.96143 | 1.62552 | 0.47087 | 3.45217 |  |
| 1090 | 0.0009604 | 0.0381041 | 0.213139 | 0.059534 | 3.58013 |  |
| 1091 | 0.221372 | 2.15344 | 1.52656 | 0.376771 | 4.05169 |  |
| 1092 | 0.0643468 | 0.976725 | 0.939152 | 0.486814 | 1.92918 |  |
| 1093 | 0.0033614 | 0.105656 | 0.352346 | 0.119068 | 2.95921 |  |
| 1094 | 0.0931588 | 1.45135 | 1.32575 | 0.22752 | 5.82696 |  |
| 1095 | 0.062426 | 1.24346 | 1.30701 | 0.158203 | 8.26158 |  |
| 1096 | 0.0249704 | 0.524304 | 0.983807 | 0.277663 | 3.54317 |  |
| 1097 | 0.0729904 | 1.16924 | 1.3371 | 0.375312 | 3.56263 |  |
| 1098 | 0.0220892 | 0.386136 | 0.554869 | 0.314999 | 1.76149 |  |
| 1099 | 0.0033614 | 0.105656 | 0.352344 | 0.119068 | 2.95919 |  |
| 1100 | 0.0225694 | 0.420928 | 0.635668 | 0.274173 | 2.31849 |  |
| 1101 | 0.0595448 | 0.784275 | 0.747634 | 0.337636 | 2.21432 |  |
| 1102 | 0.115248 | 1.39572 | 1.04394 | 0.63547 | 1.64278 |  |
| 1103 | 0.0028812 | 0.0879606 | 0.318127 | 0.134435 | 2.36641 |  |
| 1104 | 0.314531 | 2.87648 | 1.67184 | 0.681694 | 2.45248 |  |
| 1105 | 0.0038416 | 0.109399 | 0.318127 | 0.119068 | 2.67181 |  |
| 1106 | 0.0729904 | 0.877152 | 0.749579 | 0.417569 | 1.7951 |  |
| 1107 | 0.217531 | 2.36926 | 1.56382 | 0.568683 | 2.7499 |  |
| 1108 | 0.0057624 | 0.149927 | 0.361977 | 0.168818 | 2.14419 |  |
| 1109 | 0.126293 | 1.5912 | 1.15956 | 0.308285 | 3.76134 |  |
| 1110 | 0.0033614 | 0.105656 | 0.359159 | 0.119068 | 3.01642 |  |
| 1111 | 0.516695 | 3.94805 | 1.74539 | 0.573132 | 3.04536 |  |
| 1112 | 0.0254506 | 0.437607 | 0.626524 | 0.278102 | 2.25286 |  |
| 1113 | 0.0297724 | 0.566197 | 0.721833 | 0.245737 | 2.93742 |  |
| 1114 | 0.0019208 | 0.0603798 | 0.219568 | 0.127083 | 1.72775 |  |
| 1115 | 0.0019208 | 0.062527 | 0.288523 | 0.164296 | 1.75611 |  |
| 1116 | 0.150783 | 1.87794 | 1.51992 | 0.484444 | 3.13745 |  |
| 1117 | 0.0485002 | 0.736685 | 0.91538 | 0.349576 | 2.61854 |  |
| 1118 | 0.0412972 | 0.662578 | 0.720508 | 0.378033 | 1.90594 |  |
| 1119 | 0.0009604 | 0.032799 | 0.109284 | 0.143766 | 0.760153 |  |
| 1120 | 0.0052822 | 0.141543 | 0.361977 | 0.128852 | 2.80926 |  |
| 1121 | 0.043218 | 0.791848 | 1.00755 | 0.268318 | 3.75507 |  |
| 1122 | 0.0057624 | 0.16039 | 0.450859 | 0.204052 | 2.20952 |  |
| 1123 | 0.0004802 | 0.019606 | 0.059534 | 0.059534 | 1 |  |
| 1124 | 0.170951 | 2.05183 | 1.49393 | 0.588265 | 2.53956 |  |
| 1125 | 0.125332 | 1.72464 | 1.43752 | 0.545959 | 2.63303 |  |
| 1126 | 0.291481 | 3.22479 | 1.70749 | 0.72924 | 2.34146 |  |
| 1127 | 0.002401 | 0.0758411 | 0.286624 | 0.119068 | 2.40724 |  |
| 1128 | 0.0004802 | 0.019606 | 0.059534 | 0.059534 | 1 |  |
| 1129 | 0.0038416 | 0.113203 | 0.361977 | 0.128852 | 2.80926 |  |
| 1130 | 0.0153664 | 0.326265 | 0.619387 | 0.257884 | 2.40181 |  |
| 1131 | 0.0076832 | 0.185797 | 0.429937 | 0.222718 | 1.93041 |  |
| 1132 | 0.0278516 | 0.48845 | 0.625169 | 0.295476 | 2.11581 |  |
| 1133 | 0.0057624 | 0.178617 | 0.699158 | 0.155873 | 4.48544 |  |
| 1134 | 0.021609 | 0.44582 | 0.705911 | 0.277193 | 2.54664 |  |
| 1135 | 0.0571438 | 0.827598 | 0.985493 | 0.358465 | 2.7492 |  |
| 1136 | 0.0004802 | 0.019606 | 0.059534 | 0.059534 | 1 |  |
| 1137 | 0.004802 | 0.148277 | 0.445903 | 0.191371 | 2.33005 |  |
| 1138 | 0.0062426 | 0.162257 | 0.426322 | 0.168818 | 2.52533 |  |
| 1139 | 0.0485002 | 0.821873 | 1.1173 | 0.429892 | 2.59904 |  |
| 1140 | 0.150783 | 1.61243 | 1.2262 | 0.427353 | 2.8693 |  |
| 1141 | 0.0139258 | 0.277247 | 0.491903 | 0.228352 | 2.15414 |  |
| 1142 | 0.254506 | 2.78979 | 2.60431 | 0.367819 | 7.08042 |  |
| 1143 | 0.169511 | 1.94355 | 1.33194 | 0.39717 | 3.35358 |  |
| 1144 | 0.0436982 | 0.870309 | 0.913524 | 0.168818 | 5.41129 |  |
| 1145 | 0.0009604 | 0.032799 | 0.109284 | 0.143766 | 0.760155 |  |
| 1146 | 1.07181 | 7.07935 | 2.10466 | 1.02269 | 2.05796 |  |
| 1147 | 0.0302526 | 0.51735 | 0.657542 | 0.278102 | 2.36439 |  |
| 1148 | 0.0081634 | 0.187367 | 0.379706 | 0.237217 | 1.60067 |  |
| 1149 | 0.0158466 | 0.315063 | 0.521537 | 0.218568 | 2.38615 |  |
| 1150 | 0.0067228 | 0.165275 | 0.358132 | 0.211945 | 1.68974 |  |
| 1151 | 0.0187278 | 0.372247 | 0.634631 | 0.228352 | 2.77918 |  |
| 1152 | 0.004802 | 0.130024 | 0.359161 | 0.159034 | 2.25838 |  |
| 1153 | 0.0441784 | 0.72265 | 0.907044 | 0.257703 | 3.51973 |  |
| 1154 | 0.0033614 | 0.105656 | 0.356027 | 0.119068 | 2.99011 |  |
| 1155 | 0.004802 | 0.129074 | 0.356028 | 0.182203 | 1.95402 |  |
| 1156 | 0.0129654 | 0.279228 | 0.494509 | 0.178602 | 2.76878 |  |
| 1157 | 0.0009604 | 0.032799 | 0.109284 | 0.143766 | 0.760153 |  |
| 1158 | 0.0417774 | 0.708069 | 0.831895 | 0.238136 | 3.49337 |  |
| 1159 | 0.0177674 | 0.40921 | 0.689683 | 0.159034 | 4.33669 |  |
| 1160 | 0.0148862 | 0.376804 | 0.803342 | 0.208785 | 3.8477 |  |
| 1161 | 0.0139258 | 0.289443 | 0.511803 | 0.178602 | 2.86561 |  |
| 1162 | 0.0182476 | 0.368664 | 0.572703 | 0.178602 | 3.20659 |  |
| 1163 | 0.662676 | 5.77612 | 2.97534 | 0.725022 | 4.10379 |  |
| 1164 | 0.0009604 | 0.032799 | 0.109284 | 0.143766 | 0.760151 |  |
| 1165 | 0.0100842 | 0.239965 | 0.52449 | 0.199001 | 2.63562 |  |
| 1166 | 0.0153664 | 0.332872 | 0.649507 | 0.218568 | 2.97164 |  |
| 1167 | 0.0019208 | 0.0603798 | 0.22897 | 0.140504 | 1.62963 |  |
| 1168 | 0.016807 | 0.400336 | 0.724481 | 0.159034 | 4.5555 |  |
| 1169 | 0.0033614 | 0.110289 | 0.415612 | 0.119068 | 3.49055 |  |
| 1170 | 0.0038416 | 0.121483 | 0.352344 | 0.119068 | 2.95919 |  |
| 1171 | 0.0019208 | 0.0728226 | 0.279906 | 0.069318 | 4.03802 |  |
| 1172 | 0.0009604 | 0.032799 | 0.109284 | 0.143766 | 0.760153 |  |
| 1173 | 0.0158466 | 0.422668 | 0.931872 | 0.178602 | 5.2176 |  |
| 1174 | 0.147421 | 2.21038 | 1.7925 | 0.526853 | 3.40228 |  |
| 1175 | 0.653552 | 4.87208 | 2.14462 | 0.641437 | 3.34346 |  |
| 1176 | 0.0331338 | 0.55987 | 0.712056 | 0.228352 | 3.11824 |  |
| 1177 | 3.97462 | 23.4408 | 5.27145 | 1.88597 | 2.79508 |  |
| 1178 | 0.0494606 | 0.95156 | 1.30654 | 0.378435 | 3.4525 |  |
| 1179 | 0.016807 | 0.343254 | 0.633456 | 0.208785 | 3.03401 |  |
| 1180 | 0.108525 | 1.53285 | 1.31701 | 0.503388 | 2.61629 |  |
| 1181 | 0.019208 | 0.404508 | 0.668854 | 0.199001 | 3.36106 |  |
| 1182 | 0.0350546 | 0.663304 | 0.844002 | 0.257703 | 3.2751 |  |
| 1183 | 0.0854756 | 1.28698 | 1.36805 | 0.247919 | 5.51814 |  |
| 1184 | 0.0662676 | 0.933421 | 0.97428 | 0.298501 | 3.26391 |  |
| 1185 | 0.0100842 | 0.24355 | 0.491925 | 0.168818 | 2.91394 |  |
| 1186 | 0.110446 | 2.14136 | 1.53023 | 0.216905 | 7.05484 |  |
| 1187 | 0.189679 | 2.04894 | 1.35454 | 0.424995 | 3.18718 |  |
| 1188 | 0.0134456 | 0.302637 | 0.576923 | 0.224627 | 2.56836 |  |
| 1189 | 0.0220892 | 0.497407 | 0.876247 | 0.178602 | 4.90615 |  |
| 1190 | 0.273234 | 2.99536 | 2.40769 | 0.475439 | 5.06414 |  |
| 1191 | 1.93232 | 14.2087 | 3.95724 | 0.850546 | 4.65258 |  |
| 1192 | 0.0052822 | 0.156027 | 0.416495 | 0.183284 | 2.2724 |  |
| 1193 | 0.0369754 | 0.536842 | 0.589547 | 0.361921 | 1.62894 |  |
| 1194 | 0.0033614 | 0.106213 | 0.364011 | 0.119068 | 3.05717 |  |
| 1195 | 0.0609854 | 0.925531 | 0.987359 | 0.301694 | 3.27271 |  |
| 1196 | 0.21705 | 2.86884 | 1.9771 | 0.39717 | 4.97797 |  |
| 1197 | 0.145501 | 1.96092 | 1.36688 | 0.366355 | 3.73102 |  |
| 1198 | 0.036015 | 0.719627 | 1.08593 | 0.262585 | 4.13554 |  |
| 1199 | 0.0014406 | 0.0497959 | 0.219564 | 0.127085 | 1.72769 |  |
| 1200 | 0.014406 | 0.335412 | 0.71243 | 0.138635 | 5.13888 |  |
| 1201 | 0.0019208 | 0.0728226 | 0.279906 | 0.069318 | 4.03802 |  |
| 1202 | 0.214169 | 2.68198 | 1.72491 | 0.525171 | 3.28446 |  |
| 1203 | 0.0057624 | 0.155469 | 0.359161 | 0.119068 | 3.01644 |  |
| 1204 | 0.004802 | 0.141123 | 0.356028 | 0.159034 | 2.23869 |  |
| 1205 | 0.0043218 | 0.131701 | 0.411102 | 0.119068 | 3.45267 |  |
| 1206 | 0.0076832 | 0.198728 | 0.523831 | 0.168818 | 3.10293 |  |
| 1207 | 10.4175 | 54.9455 | 7.12539 | 2.67095 | 2.66773 |  |
| 1208 | 0.0038416 | 0.114704 | 0.362054 | 0.119068 | 3.04074 |  |
| 1209 | 0.0182476 | 0.41168 | 0.817615 | 0.299646 | 2.7286 |  |
| 1210 | 0.060025 | 0.786384 | 0.891809 | 0.327852 | 2.72016 |  |
| 1211 | 0.0998816 | 1.19159 | 0.982606 | 0.433194 | 2.26828 |  |
| 1212 | 0.0019208 | 0.0667585 | 0.285061 | 0.161203 | 1.76834 |  |
| 1213 | 0.0067228 | 0.188669 | 0.440451 | 0.186765 | 2.35831 |  |
| 1214 | 0.0235298 | 0.464679 | 0.698847 | 0.318691 | 2.19287 |  |
| 1215 | 0.0004802 | 0.019606 | 0.059534 | 0.059534 | 1 |  |
| 1216 | 0.0014406 | 0.0497959 | 0.237961 | 0.119739 | 1.98733 |  |
| 1217 | 0.060025 | 1.24751 | 1.4825 | 0.32702 | 4.53336 |  |
| 1218 | 0.0355348 | 0.613042 | 0.848992 | 0.248751 | 3.41302 |  |
| 1219 | 1.30758 | 11.1997 | 4.2452 | 0.964822 | 4.39999 |  |
| 1220 | 0.0701092 | 1.09075 | 1.18705 | 0.257703 | 4.60627 |  |
| 1221 | 0.043218 | 0.739464 | 0.927383 | 0.238135 | 3.89435 |  |
| 1222 | 0.0009604 | 0.032799 | 0.109284 | 0.143762 | 0.760172 |  |
| 1223 | 0.136857 | 2.09888 | 2.10238 | 0.551793 | 3.8101 |  |
| 1224 | 0.0134456 | 0.323415 | 0.644338 | 0.199001 | 3.23786 |  |
| 1225 | 0.0134456 | 0.306382 | 0.566551 | 0.245111 | 2.31141 |  |
| 1226 | 0.009604 | 0.249346 | 0.631629 | 0.178602 | 3.53652 |  |
| 1227 | 0.0105644 | 0.254726 | 0.513575 | 0.224627 | 2.28635 |  |
| 1228 | 0.012005 | 0.270879 | 0.477203 | 0.245292 | 1.94544 |  |
| 1229 | 0.004802 | 0.13698 | 0.359159 | 0.119068 | 3.01643 |  |
| 1230 | 0.0153664 | 0.372204 | 0.915417 | 0.226511 | 4.04138 |  |
| 1231 | 0.0057624 | 0.167719 | 0.445484 | 0.149251 | 2.98481 |  |
| 1232 | 0.0009604 | 0.0381041 | 0.208249 | 0.092506 | 2.25121 |  |
| 1233 | 0.0028812 | 0.0984743 | 0.352344 | 0.119068 | 2.95919 |  |
| 1234 | 0.007203 | 0.181531 | 0.415612 | 0.168818 | 2.46189 |  |
| 1235 | 0.036015 | 0.58436 | 0.766074 | 0.30373 | 2.52222 |  |
| 1236 | 0.0009604 | 0.032799 | 0.109284 | 0.143766 | 0.760155 |  |
| 1237 | 0.062426 | 0.983751 | 1.09052 | 0.357852 | 3.0474 |  |
| 1238 | 0.021609 | 0.59329 | 1.46973 | 0.239152 | 6.14557 |  |
| 1239 | 0.0067228 | 0.180929 | 0.497696 | 0.128852 | 3.86255 |  |
| 1240 | 0.0009604 | 0.0381041 | 0.213139 | 0.059534 | 3.58012 |  |
| 1241 | 0.0014406 | 0.0497959 | 0.233949 | 0.135086 | 1.73185 |  |
| 1242 | 0.0014406 | 0.0560173 | 0.275416 | 0.069318 | 3.97325 |  |
| 1243 | 0.0009604 | 0.0381041 | 0.20825 | 0.092508 | 2.25116 |  |
| 1244 | 0.0057624 | 0.163915 | 0.425645 | 0.199447 | 2.13413 |  |
| 1245 | 0.0009604 | 0.0381041 | 0.208249 | 0.092508 | 2.25115 |  |
| 1246 | 0.0019208 | 0.0728226 | 0.279898 | 0.069318 | 4.0379 |  |
| 1247 | 0.0009604 | 0.0381041 | 0.208249 | 0.092504 | 2.25125 |  |
| 1248 | 0.0019208 | 0.0603798 | 0.219568 | 0.127085 | 1.72772 |  |
| 1249 | 0.004802 | 0.128691 | 0.361977 | 0.128852 | 2.80926 |  |
| 1250 | 0.0139258 | 0.3166 | 0.609442 | 0.178602 | 3.4123 |  |
| 1251 | 0.0009604 | 0.032799 | 0.109284 | 0.143766 | 0.760153 |  |
| 1252 | 0.0038416 | 0.104181 | 0.293123 | 0.119068 | 2.46182 |  |
| 1253 | 0.0067228 | 0.172492 | 0.445903 | 0.119068 | 3.74494 |  |
| 1254 | 0.0201684 | 0.449538 | 0.977385 | 0.178602 | 5.47243 |  |
| 1255 | 0.0605052 | 1.05107 | 1.27869 | 0.237304 | 5.38841 |  |
| 1256 | 0.026411 | 0.552209 | 0.999684 | 0.22752 | 4.39383 |  |
| 1257 | 0.0033614 | 0.0994345 | 0.312581 | 0.137894 | 2.26682 |  |
| 1258 | 0.009604 | 0.232634 | 0.511807 | 0.178602 | 2.86563 |  |
| 1259 | 0.0100842 | 0.23807 | 0.572703 | 0.178602 | 3.20659 |  |
| 1260 | 0.0134456 | 0.309061 | 0.512971 | 0.188385 | 2.72298 |  |
| 1261 | 0.0201684 | 0.465891 | 0.983855 | 0.158202 | 6.21896 |  |
| 1262 | 0.498928 | 4.56621 | 2.26659 | 0.596171 | 3.80192 |  |
| 1263 | 0.0211288 | 0.458168 | 0.696402 | 0.148419 | 4.69214 |  |
| 1264 | 0.0105644 | 0.257023 | 0.55083 | 0.138635 | 3.97324 |  |
| 1265 | 0.0148862 | 0.386569 | 0.960227 | 0.17777 | 5.40152 |  |
| 1266 | 0.0009604 | 0.032799 | 0.109284 | 0.143764 | 0.760163 |  |
| 1267 | 0.0086436 | 0.213222 | 0.445903 | 0.128852 | 3.4606 |  |
| 1268 | 0.454269 | 4.40108 | 2.39436 | 0.616865 | 3.88149 |  |
| 1269 | 0.0057624 | 0.156271 | 0.435763 | 0.138635 | 3.14324 |  |
| 1270 | 0.0009604 | 0.032799 | 0.109284 | 0.143766 | 0.760152 |  |
| 1271 | 0.0446586 | 0.749682 | 0.912297 | 0.228352 | 3.99513 |  |
| 1272 | 0.16759 | 2.18753 | 1.72451 | 0.481448 | 3.58193 |  |
| 1273 | 0.0038416 | 0.122782 | 0.365524 | 0.205168 | 1.78159 |  |
| 1274 | 0.74431 | 5.52725 | 2.52971 | 0.576603 | 4.38726 |  |
| 1275 | 0.147421 | 1.75012 | 1.42865 | 0.456265 | 3.13119 |  |
| 1276 | 0.141179 | 1.45582 | 1.04785 | 0.460154 | 2.27717 |  |
| 1277 | 0.0355348 | 0.529555 | 0.605007 | 0.318069 | 1.90213 |  |
| 1278 | 0.0460992 | 0.90577 | 1.2603 | 0.324203 | 3.88737 |  |
| 1279 | 0.0038416 | 0.104181 | 0.293121 | 0.119068 | 2.46179 |  |
| 1280 | 0.104203 | 1.58328 | 1.25375 | 0.34742 | 3.60875 |  |
| 1281 | 0.014406 | 0.375176 | 0.992192 | 0.195053 | 5.08677 |  |
| 1282 | 0.209367 | 2.7467 | 2.30333 | 0.52723 | 4.36874 |  |
| 1283 | 0.0124852 | 0.306042 | 0.657541 | 0.138635 | 4.74296 |  |
| 1284 | 0.0019208 | 0.0603798 | 0.228973 | 0.140506 | 1.62963 |  |
| 1285 | 0.0100842 | 0.23993 | 0.511166 | 0.168818 | 3.02791 |  |
| 1286 | 0.0014406 | 0.0497959 | 0.237959 | 0.119736 | 1.98736 |  |
| 1287 | 0.0076832 | 0.189044 | 0.429382 | 0.168818 | 2.54346 |  |
| 1288 | 2.87304 | 25.3026 | 5.28806 | 1.51687 | 3.48618 |  |
| 1289 | 0.0004802 | 0.019606 | 0.059534 | 0.059534 | 1 |  |
| 1290 | 0.0009604 | 0.032799 | 0.109284 | 0.143766 | 0.760152 |  |
| 1291 | 0.033614 | 0.590007 | 0.770548 | 0.30373 | 2.53695 |  |
| 1292 | 0.0806736 | 1.23307 | 1.48619 | 0.459605 | 3.23362 |  |
| 1293 | 8.61623 | 53.869 | 8.88138 | 2.19674 | 4.04299 |  |
| 1294 | 0.174793 | 2.08939 | 1.31989 | 0.346588 | 3.80825 |  |
| 1295 | 0.114288 | 1.49289 | 1.24859 | 0.576728 | 2.16496 |  |
| 1296 | 0.036015 | 0.674347 | 0.924879 | 0.248751 | 3.71809 |  |
| 1297 | 0.0662676 | 1.00087 | 1.04903 | 0.348252 | 3.01228 |  |
| 1298 | 0.12005 | 1.8308 | 1.68105 | 0.338468 | 4.96665 |  |
| 1299 | 0.0124852 | 0.285275 | 0.532649 | 0.199001 | 2.67662 |  |
| 1300 | 0.131575 | 1.40719 | 1.04639 | 0.467319 | 2.23914 |  |
| 1301 | 0.033614 | 0.638819 | 0.844078 | 0.257703 | 3.27539 |  |
| 1302 | 0.16807 | 1.86863 | 1.27706 | 0.307453 | 4.15366 |  |
| 1303 | 5.68125 | 35.0618 | 4.64519 | 1.99576 | 2.32754 |  |
| 1304 | 0.0129654 | 0.303362 | 0.631381 | 0.168818 | 3.74001 |  |
| 1305 | 0.0009604 | 0.032799 | 0.109284 | 0.143766 | 0.760155 |  |
| 1306 | 0.319813 | 3.72155 | 2.37336 | 0.414242 | 5.7294 |  |
| 1307 | 0.002401 | 0.0820625 | 0.293123 | 0.109284 | 2.68222 |  |
| 1308 | 0.0004802 | 0.019606 | 0.059534 | 0.059534 | 1 |  |
| 1309 | 0.0076832 | 0.20222 | 0.576743 | 0.128852 | 4.47602 |  |
| 1310 | 0.009604 | 0.232843 | 0.426044 | 0.168818 | 2.52369 |  |
| 1311 | 0.0038416 | 0.104181 | 0.293123 | 0.119068 | 2.46181 |  |
| 1312 | 0.0057624 | 0.166829 | 0.505013 | 0.138635 | 3.64274 |  |
| 1313 | 0.0605052 | 0.962084 | 1.05831 | 0.287885 | 3.67617 |  |
| 1314 | 0.0081634 | 0.228273 | 0.657542 | 0.178602 | 3.68161 |  |
| 1315 | 0.0412972 | 0.939651 | 1.63731 | 0.237304 | 6.89963 |  |
| 1316 | 0.009604 | 0.243393 | 0.51297 | 0.219929 | 2.33243 |  |
| 1317 | 0.002401 | 0.0773766 | 0.320168 | 0.134435 | 2.38159 |  |
| 1318 | 0.578161 | 7.9658 | 3.62109 | 0.714407 | 5.06866 |  |
| 1319 | 0.0057624 | 0.163181 | 0.414598 | 0.159034 | 2.60697 |  |
| 1320 | 0.0782726 | 1.09422 | 1.00682 | 0.27727 | 3.63119 |  |
| 1321 | 0.0134456 | 0.304358 | 0.53947 | 0.199001 | 2.71089 |  |
| 1322 | 0.0067228 | 0.172492 | 0.435763 | 0.138635 | 3.14323 |  |
| 1323 | 0.0038416 | 0.11624 | 0.36401 | 0.1393 | 2.61315 |  |
| 1324 | 0.0019208 | 0.0603798 | 0.228973 | 0.140504 | 1.62965 |  |
| 1325 | 0.014406 | 0.330194 | 0.630974 | 0.148419 | 4.25131 |  |
| 1326 | 0.0100842 | 0.229651 | 0.494509 | 0.178602 | 2.76878 |  |
| 1327 | 0.0436982 | 0.669876 | 0.888682 | 0.316411 | 2.80863 |  |
| 1328 | 0.0825944 | 1.22987 | 1.36997 | 0.392968 | 3.48622 |  |
| 1329 | 0.0105644 | 0.257554 | 0.51297 | 0.188385 | 2.72298 |  |
| 1330 | 0.0523418 | 0.915081 | 1.39769 | 0.319016 | 4.38126 |  |
| 1331 | 0.012005 | 0.251168 | 0.435763 | 0.208785 | 2.08714 |  |
| 1332 | 0.0028812 | 0.0879606 | 0.318123 | 0.134435 | 2.36638 |  |
| 1333 | 0.004802 | 0.128691 | 0.366906 | 0.119068 | 3.08148 |  |
| 1334 | 0.0009604 | 0.032799 | 0.109284 | 0.143766 | 0.760156 |  |
| 1335 | 0.0019208 | 0.0603798 | 0.22897 | 0.140504 | 1.62963 |  |
| 1336 | 0.0009604 | 0.032799 | 0.109284 | 0.143765 | 0.760158 |  |
| 1337 | 0.0259308 | 0.488802 | 0.73868 | 0.298317 | 2.47616 |  |
| 1338 | 0.0926786 | 1.22957 | 1.02181 | 0.472458 | 2.16275 |  |
| 1339 | 0.0086436 | 0.209775 | 0.429406 | 0.178602 | 2.40426 |  |
| 1340 | 0.0062426 | 0.17318 | 0.491929 | 0.204052 | 2.4108 |  |
| 1341 | 0.0350546 | 0.547486 | 0.655427 | 0.319017 | 2.05452 |  |
| 1342 | 0.007203 | 0.189226 | 0.426036 | 0.168818 | 2.52364 |  |
| 1343 | 0.0907578 | 1.3825 | 1.5089 | 0.427269 | 3.5315 |  |
| 1344 | 0.0028812 | 0.0984743 | 0.356215 | 0.119068 | 2.9917 |  |
| 1345 | 0.125332 | 1.67014 | 1.35307 | 0.257703 | 5.2505 |  |
| 1346 | 0.0211288 | 0.476482 | 0.695071 | 0.148419 | 4.68317 |  |
| 1347 | 0.0134456 | 0.289471 | 0.511803 | 0.178602 | 2.86561 |  |
| 1348 | 0.0009604 | 0.032799 | 0.109284 | 0.143765 | 0.760158 |  |
| 1349 | 0.0595448 | 0.940468 | 1.16914 | 0.337636 | 3.46272 |  |
| 1350 | 0.642988 | 5.01939 | 2.31823 | 0.834964 | 2.77644 |  |
| 1351 | 0.004802 | 0.136796 | 0.359161 | 0.168818 | 2.1275 |  |
| 1352 | 0.036015 | 0.695707 | 1.19222 | 0.218568 | 5.45466 |  |
| 1353 | 20.0459 | 106.916 | 9.29484 | 3.10159 | 2.9968 |  |
| 1354 | 0.0369754 | 0.5962 | 0.829928 | 0.306097 | 2.71133 |  |
| 1355 | 0.0038416 | 0.104181 | 0.293123 | 0.119068 | 2.46181 |  |
| 1356 | 0.016807 | 0.347949 | 0.639966 | 0.218568 | 2.92799 |  |
| 1357 | 0.107565 | 1.41959 | 1.27678 | 0.308285 | 4.14156 |  |
| 1358 | 0.0033614 | 0.108108 | 0.360911 | 0.128851 | 2.80099 |  |
| 1359 | 0.0052822 | 0.163574 | 0.505622 | 0.17878 | 2.82818 |  |
| 1360 | 0.0019208 | 0.0667927 | 0.320172 | 0.146679 | 2.18281 |  |
| 1361 | 0.002401 | 0.0826196 | 0.304724 | 0.137894 | 2.20983 |  |
| 1362 | 0.0019208 | 0.0603798 | 0.219564 | 0.127087 | 1.72767 |  |
| 1363 | 0.0201684 | 0.455455 | 0.781526 | 0.138635 | 5.63727 |  |
| 1364 | 0.0388962 | 0.759095 | 1.22803 | 0.207121 | 5.92906 |  |
| 1365 | 0.142619 | 2.36575 | 2.36123 | 0.424857 | 5.55771 |  |
| 1366 | 0.0086436 | 0.211564 | 0.450285 | 0.214032 | 2.10382 |  |
| 1367 | 0.0086436 | 0.200804 | 0.427591 | 0.237218 | 1.80252 |  |
| 1368 | 0.0417774 | 0.683195 | 0.797577 | 0.268318 | 2.9725 |  |
| 1369 | 0.038416 | 0.630926 | 0.840542 | 0.247919 | 3.39039 |  |
| 1370 | 0.009604 | 0.257381 | 0.740036 | 0.128851 | 5.74333 |  |
| 1371 | 0.0638666 | 1.1739 | 1.12044 | 0.238136 | 4.70506 |  |
| 1372 | 0.458111 | 6.27712 | 2.6299 | 0.344924 | 7.62456 |  |
| 1373 | 0.0081634 | 0.204348 | 0.489747 | 0.183607 | 2.66736 |  |
| 1374 | 0.0172872 | 0.350043 | 0.580781 | 0.178602 | 3.25181 |  |
| 1375 | 0.0533022 | 0.812623 | 0.972488 | 0.29767 | 3.267 |  |
| 1376 | 0.0485002 | 1.05767 | 1.77445 | 0.286222 | 6.19956 |  |
| 1377 | 0.0105644 | 0.252897 | 0.491903 | 0.159034 | 3.09306 |  |
| 1378 | 0.0067228 | 0.169421 | 0.361977 | 0.128852 | 2.80926 |  |
| 1379 | 0.0398566 | 0.78931 | 1.25922 | 0.247088 | 5.09625 |  |
| 1380 | 0.0038416 | 0.123055 | 0.494509 | 0.204825 | 2.4143 |  |
| 1381 | 0.0004802 | 0.019606 | 0.059534 | 0.059534 | 1 |  |
| 1382 | 0.0004802 | 0.019606 | 0.059534 | 0.059534 | 1 |  |
| 1383 | 0.0004802 | 0.019606 | 0.059534 | 0.059534 | 1 |  |
| 1384 | 0.0019208 | 0.0603798 | 0.228969 | 0.140505 | 1.62962 |  |
| 1385 | 0.0283318 | 0.624903 | 1.18104 | 0.168818 | 6.99595 |  |
| 1386 | 0.0019208 | 0.0728226 | 0.279906 | 0.069318 | 4.03802 |  |
| 1387 | 0.02401 | 0.518189 | 0.727485 | 0.138635 | 5.24748 |  |
| 1388 | 0.0153664 | 0.343935 | 0.63524 | 0.188385 | 3.37202 |  |
| 1389 | 0.447066 | 4.29584 | 2.02983 | 0.557097 | 3.64359 |  |
| 1390 | 0.0009604 | 0.032799 | 0.109284 | 0.143766 | 0.760153 |  |
| 1391 | 0.004802 | 0.132478 | 0.356028 | 0.159034 | 2.23869 |  |
| 1392 | 0.0038416 | 0.115541 | 0.413389 | 0.141786 | 2.91558 |  |
| 1393 | 0.0009604 | 0.032799 | 0.109284 | 0.143766 | 0.760153 |  |
| 1394 | 0.007203 | 0.181374 | 0.42604 | 0.168818 | 2.52367 |  |
| 1395 | 0.0115248 | 0.292893 | 0.705915 | 0.167986 | 4.20222 |  |
| 1396 | 0.0662676 | 1.31048 | 1.28075 | 0.207121 | 6.18357 |  |
| 1397 | 0.0091238 | 0.276804 | 0.765066 | 0.158203 | 4.83598 |  |
| 1398 | 0.0057624 | 0.170703 | 0.602093 | 0.158759 | 3.79251 |  |
| 1399 | 0.0019208 | 0.0603798 | 0.22897 | 0.140504 | 1.62963 |  |
| 1400 | 0.0374556 | 0.786721 | 1.00682 | 0.187553 | 5.36818 |  |
| 1401 | 0.0196882 | 0.419288 | 0.689679 | 0.178602 | 3.86155 |  |
| 1402 | 0.014406 | 0.369493 | 0.94743 | 0.138635 | 6.83399 |  |
| 1403 | 0.0038416 | 0.104181 | 0.293119 | 0.119068 | 2.46178 |  |
| 1404 | 0.0019208 | 0.0662077 | 0.279906 | 0.161793 | 1.73002 |  |
| 1405 | 0.0067228 | 0.172492 | 0.445903 | 0.119068 | 3.74494 |  |
| 1406 | 0.145501 | 2.12045 | 1.62631 | 0.464945 | 3.49784 |  |
| 1407 | 0.0148862 | 0.348264 | 0.609442 | 0.158202 | 3.85229 |  |
| 1408 | 0.267471 | 4.50561 | 2.42791 | 0.394674 | 6.15169 |  |
| 1409 | 0.0100842 | 0.229668 | 0.491903 | 0.236023 | 2.08413 |  |
| 1410 | 0.154624 | 1.60889 | 1.15122 | 0.357203 | 3.22287 |  |
| 1411 | 0.0057624 | 0.161129 | 0.403654 | 0.159034 | 2.53816 |  |
| 1412 | 0.535903 | 4.46388 | 1.94646 | 0.577435 | 3.37088 |  |
| 1413 | 0.351026 | 2.79957 | 1.44673 | 0.516238 | 2.80245 |  |
| 1414 | 0.0187278 | 0.396096 | 0.756606 | 0.313832 | 2.41086 |  |
| 1415 | 1.16112 | 7.0828 | 2.61044 | 0.68256 | 3.82448 |  |
| 1416 | 0.0009604 | 0.032799 | 0.109284 | 0.143765 | 0.760156 |  |
| 1417 | 0.0609854 | 0.810409 | 0.856196 | 0.398382 | 2.14918 |  |
| 1418 | 0.123411 | 1.60565 | 1.23541 | 0.41262 | 2.99407 |  |
| 1419 | 0.076832 | 1.13136 | 1.32575 | 0.257703 | 5.1445 |  |
| 1420 | 0.0513814 | 0.879086 | 1.01064 | 0.287054 | 3.52072 |  |
| 1421 | 0.108045 | 1.3456 | 1.24575 | 0.467377 | 2.6654 |  |
| 1422 | 0.0259308 | 0.490468 | 0.791118 | 0.207953 | 3.80432 |  |
| 1423 | 0.157986 | 2.07568 | 2.08054 | 0.465404 | 4.47039 |  |
| 1424 | 0.0177674 | 0.397151 | 0.847261 | 0.198169 | 4.27544 |  |
| 1425 | 0.0292922 | 0.522523 | 0.786901 | 0.299643 | 2.62613 |  |
| 1426 | 0.667958 | 4.94257 | 1.71865 | 1.012 | 1.69826 |  |
| 1427 | 0.507571 | 5.09338 | 2.5743 | 0.644258 | 3.99576 |  |
| 1428 | 0.0235298 | 0.555264 | 1.04186 | 0.239515 | 4.34986 |  |
| 1429 | 0.0633864 | 0.921858 | 0.965972 | 0.344245 | 2.80606 |  |
| 1430 | 0.079233 | 1.27259 | 1.28005 | 0.405284 | 3.1584 |  |
| 1431 | 0.0033614 | 0.100942 | 0.318127 | 0.203218 | 1.56545 |  |
| 1432 | 0.0009604 | 0.0381041 | 0.213139 | 0.059534 | 3.58012 |  |
| 1433 | 0.228095 | 3.35498 | 4.35787 | 0.457535 | 9.52467 |  |
| 1434 | 0.933989 | 6.45981 | 3.10268 | 0.635305 | 4.88376 |  |
| 1435 | 0.0004802 | 0.019606 | 0.059534 | 0.059534 | 1 |  |
| 1436 | 0.0316932 | 0.66146 | 0.832462 | 0.167986 | 4.95554 |  |
| 1437 | 0.161347 | 2.51115 | 1.73497 | 0.228352 | 7.59777 |  |
| 1438 | 0.007203 | 0.176862 | 0.435763 | 0.168818 | 2.58126 |  |
| 1439 | 0.0009604 | 0.032799 | 0.109284 | 0.143766 | 0.760153 |  |
| 1440 | 0.489324 | 4.31088 | 2.52884 | 0.416738 | 6.06818 |  |
| 1441 | 0.0009604 | 0.0381041 | 0.213139 | 0.059534 | 3.58012 |  |
| 1442 | 0.0393764 | 0.651241 | 0.751239 | 0.257703 | 2.91514 |  |
| 1443 | 0.0081634 | 0.208858 | 0.499376 | 0.224627 | 2.22314 |  |
| 1444 | 0.0009604 | 0.0381041 | 0.213139 | 0.059534 | 3.58013 |  |
| 1445 | 0.439383 | 4.38809 | 3.3761 | 0.417569 | 8.08514 |  |
| 1446 | 0.0028812 | 0.092253 | 0.320172 | 0.119068 | 2.68899 |  |
| 1447 | 0.248744 | 3.08387 | 2.89665 | 0.308285 | 9.39601 |  |
| 1448 | 0.086436 | 1.35828 | 1.18161 | 0.237304 | 4.97931 |  |
| 1449 | 1.12559 | 7.27614 | 2.48989 | 0.843258 | 2.9527 |  |
| 1450 | 3.21638 | 17.434 | 3.71422 | 0.931311 | 3.98816 |  |
| 1451 | 0.038416 | 0.560663 | 0.623739 | 0.35747 | 1.74487 |  |
| 1452 | 0.0523418 | 0.945656 | 1.32884 | 0.278102 | 4.77825 |  |
| 1453 | 0.0019208 | 0.062527 | 0.293123 | 0.169676 | 1.72754 |  |
| 1454 | 0.0028812 | 0.0879606 | 0.318127 | 0.134435 | 2.36641 |  |
| 1455 | 0.0163268 | 0.334328 | 0.512974 | 0.228352 | 2.24642 |  |
| 1456 | 0.0158466 | 0.357033 | 0.667289 | 0.228028 | 2.92634 |  |
| 1457 | 0.019208 | 0.363025 | 0.587059 | 0.228352 | 2.57085 |  |
| 1458 | 0.0086436 | 0.206249 | 0.444861 | 0.178602 | 2.4908 |  |
| 1459 | 0.0033614 | 0.105634 | 0.304724 | 0.205167 | 1.48525 |  |
| 1460 | 0.0052822 | 0.144152 | 0.449157 | 0.138635 | 3.23985 |  |
| 1461 | 0.004802 | 0.128691 | 0.366906 | 0.119068 | 3.08148 |  |
| 1462 | 0.019208 | 0.386793 | 0.601481 | 0.188385 | 3.19282 |  |
| 1463 | 1.34648 | 12.3739 | 3.56165 | 1.11739 | 3.18747 |  |
| 1464 | 0.109486 | 2.22809 | 2.09216 | 0.256871 | 8.14477 |  |
| 1465 | 0.0297724 | 0.652622 | 1.13404 | 0.207121 | 5.47526 |  |
| 1466 | 0.0115248 | 0.278462 | 0.521535 | 0.128851 | 4.04757 |  |
| 1467 | 0.0057624 | 0.163209 | 0.411103 | 0.184688 | 2.22593 |  |
| 1468 | 0.009604 | 0.235584 | 0.529747 | 0.148419 | 3.56927 |  |
| 1469 | 0.0009604 | 0.032799 | 0.109284 | 0.143766 | 0.760153 |  |
| 1470 | 0.0369754 | 0.586961 | 0.710667 | 0.247919 | 2.86653 |  |
| 1471 | 0.0076832 | 0.191783 | 0.435765 | 0.138635 | 3.14325 |  |
| 1472 | 0.0081634 | 0.213613 | 0.498911 | 0.168818 | 2.95532 |  |
| 1473 | 0.0009604 | 0.032799 | 0.109284 | 0.143766 | 0.760151 |  |
| 1474 | 0.0038416 | 0.104181 | 0.293119 | 0.119068 | 2.46179 |  |
| 1475 | 0.542146 | 5.31028 | 3.30714 | 0.766855 | 4.31261 |  |
| 1476 | 0.0729904 | 1.19118 | 1.60047 | 0.358867 | 4.45979 |  |
| 1477 | 0.19304 | 2.14061 | 1.59222 | 0.336804 | 4.72743 |  |
| 1478 | 0.09604 | 1.5223 | 1.54387 | 0.432241 | 3.57177 |  |
| 1479 | 0.004802 | 0.129074 | 0.364012 | 0.20233 | 1.7991 |  |
| 1480 | 0.0052822 | 0.140784 | 0.364012 | 0.159034 | 2.28889 |  |
| 1481 | 0.0052822 | 0.154675 | 0.491903 | 0.119068 | 4.13129 |  |
| 1482 | 0.321734 | 3.00791 | 1.52707 | 0.545589 | 2.79894 |  |
| 1483 | 0.35967 | 3.52081 | 2.20963 | 0.740212 | 2.98514 |  |
| 1484 | 0.0148862 | 0.317174 | 0.579781 | 0.199001 | 2.91345 |  |
| 1485 | 0.0734706 | 0.995399 | 0.894603 | 0.384163 | 2.32871 |  |
| 1486 | 0.126773 | 1.79043 | 1.68864 | 0.317237 | 5.32295 |  |
| 1487 | 0.0638666 | 0.891665 | 1.13565 | 0.46713 | 2.43112 |  |
| 1488 | 0.998336 | 7.32094 | 2.94942 | 0.978555 | 3.01406 |  |
| 1489 | 0.315491 | 4.00939 | 2.26008 | 0.366155 | 6.17246 |  |
| 1490 | 0.886449 | 6.97554 | 3.03723 | 0.804124 | 3.77707 |  |
| 1491 | 0.424017 | 3.93681 | 2.0895 | 0.565156 | 3.69721 |  |
| 1492 | 8.15572 | 39.5819 | 6.03524 | 2.25417 | 2.67737 |  |
| 1493 | 0.0129654 | 0.269997 | 0.499981 | 0.253185 | 1.97476 |  |
| 1494 | 0.0009604 | 0.032799 | 0.109284 | 0.143766 | 0.760155 |  |
| 1495 | 0.0052822 | 0.145636 | 0.374242 | 0.199355 | 1.87726 |  |
| 1496 | 0.0115248 | 0.242589 | 0.477203 | 0.255225 | 1.86973 |  |
| 1497 | 0.0124852 | 0.274561 | 0.500064 | 0.276504 | 1.80852 |  |
| 1498 | 0.038416 | 0.637963 | 0.844079 | 0.301694 | 2.79779 |  |
| 1499 | 0.848994 | 8.2352 | 3.90562 | 1.088 | 3.58972 |  |
| 1500 | 0.0091238 | 0.226074 | 0.497697 | 0.138635 | 3.58997 |  |
| 1501 | 0.057624 | 0.838424 | 0.938426 | 0.375261 | 2.50073 |  |
| 1502 | 0.304447 | 3.06477 | 1.89207 | 0.495839 | 3.81591 |  |
| 1503 | 0.0110446 | 0.25094 | 0.435763 | 0.168818 | 2.58126 |  |
| 1504 | 0.0081634 | 0.199225 | 0.416547 | 0.168818 | 2.46743 |  |
| 1505 | 0.031213 | 0.511122 | 0.644108 | 0.278102 | 2.31609 |  |
| 1506 | 0.0139258 | 0.291016 | 0.499396 | 0.254644 | 1.96116 |  |
| 1507 | 0.436982 | 4.65295 | 2.07508 | 1.03331 | 2.00819 |  |
| 1508 | 0.028812 | 0.60758 | 0.822206 | 0.158203 | 5.19717 |  |
| 1509 | 0.0105644 | 0.240775 | 0.511135 | 0.208785 | 2.44814 |  |
| 1510 | 0.0893172 | 1.73293 | 1.44896 | 0.187553 | 7.72557 |  |
| 1511 | 0.0158466 | 0.341937 | 0.602091 | 0.198169 | 3.03827 |  |
| 1512 | 0.031213 | 0.649026 | 1.06461 | 0.19817 | 5.37219 |  |
| 1513 | 0.208887 | 2.30365 | 1.48294 | 0.326189 | 4.54626 |  |
| 1514 | 0.0225694 | 0.458273 | 0.767073 | 0.310234 | 2.47256 |  |
| 1515 | 0.0148862 | 0.306247 | 0.499392 | 0.249331 | 2.00293 |  |
| 1516 | 0.0004802 | 0.019606 | 0.059534 | 0.059534 | 1 |  |
| 1517 | 0.0004802 | 0.019606 | 0.059534 | 0.059534 | 1 |  |
| 1518 | 0.0124852 | 0.294158 | 0.512974 | 0.188385 | 2.723 |  |
| 1519 | 0.137337 | 1.90942 | 1.49294 | 0.326189 | 4.57692 |  |
| 1520 | 0.0076832 | 0.194164 | 0.449157 | 0.183609 | 2.44627 |  |
| 1521 | 0.11957 | 1.63912 | 1.39652 | 0.326189 | 4.28132 |  |
| 1522 | 0.0038416 | 0.115541 | 0.40915 | 0.152028 | 2.69129 |  |
| 1523 | 0.0038416 | 0.113394 | 0.352344 | 0.205167 | 1.71735 |  |
| 1524 | 0.0004802 | 0.019606 | 0.059534 | 0.059534 | 1 |  |
| 1525 | 0.121971 | 1.62597 | 1.31993 | 0.336804 | 3.91898 |  |
| 1526 | 0.0052822 | 0.144928 | 0.361977 | 0.183605 | 1.9715 |  |
| 1527 | 0.194481 | 2.93231 | 2.26791 | 0.336804 | 6.7336 |  |
| 1528 | 1.33592 | 10.496 | 4.6165 | 0.884057 | 5.22195 |  |
| 1529 | 0.019208 | 0.395699 | 0.698847 | 0.232238 | 3.00918 |  |
| 1530 | 0.026411 | 0.482853 | 0.712056 | 0.238136 | 2.99013 |  |
| 1531 | 0.0004802 | 0.019606 | 0.059534 | 0.059534 | 1 |  |
| 1532 | 0.919103 | 8.52231 | 4.28232 | 0.983557 | 4.35391 |  |
| 1533 | 0.0009604 | 0.032799 | 0.109284 | 0.143766 | 0.760153 |  |
| 1534 | 0.231937 | 3.03097 | 2.24028 | 0.44692 | 5.0127 |  |
| 1535 | 1.35128 | 9.28396 | 2.94028 | 1.17485 | 2.50267 |  |
| 1536 | 0.0038416 | 0.109399 | 0.318127 | 0.119068 | 2.67181 |  |
| 1537 | 0.0038416 | 0.109399 | 0.318127 | 0.119068 | 2.67182 |  |
| 1538 | 0.0268912 | 0.614439 | 1.00972 | 0.158202 | 6.38243 |  |
| 1539 | 0.0009604 | 0.032799 | 0.109284 | 0.143766 | 0.760153 |  |
| 1540 | 0.043218 | 0.744525 | 0.953577 | 0.308879 | 3.08722 |  |
| 1541 | 0.130614 | 1.49812 | 1.1587 | 0.520958 | 2.22417 |  |
| 1542 | 0.424497 | 3.55861 | 1.76568 | 0.577435 | 3.0578 |  |
| 1543 | 0.0489804 | 0.797754 | 0.756861 | 0.461585 | 1.6397 |  |
| 1544 | 0.0657874 | 1.28423 | 1.40576 | 0.316405 | 4.44291 |  |
| 1545 | 0.0557032 | 0.763593 | 0.849395 | 0.412294 | 2.06017 |  |
| 1546 | 0.040817 | 0.664581 | 0.724944 | 0.287476 | 2.52176 |  |
| 1547 | 0.0326536 | 0.658875 | 0.898855 | 0.267487 | 3.36038 |  |
| 1548 | 0.0292922 | 0.533285 | 0.784686 | 0.218568 | 3.59012 |  |
| 1549 | 0.0009604 | 0.0381041 | 0.208249 | 0.092504 | 2.25125 |  |
| 1550 | 0.248744 | 2.59631 | 1.50464 | 0.455872 | 3.30057 |  |
| 1551 | 0.0849954 | 1.328 | 1.17544 | 0.207953 | 5.65245 |  |
| 1552 | 0.0009604 | 0.0381041 | 0.213139 | 0.059534 | 3.58013 |  |
| 1553 | 0.0052822 | 0.142449 | 0.364014 | 0.183283 | 1.98608 |  |
| 1554 | 0.0753914 | 1.10567 | 0.890751 | 0.484395 | 1.83889 |  |
| 1555 | 0.0028812 | 0.0848895 | 0.293115 | 0.119068 | 2.46175 |  |
| 1556 | 0.0038416 | 0.109399 | 0.361977 | 0.128851 | 2.80926 |  |
| 1557 | 1.45501 | 11.7229 | 3.62698 | 1.22169 | 2.96882 |  |
| 1558 | 0.21513 | 2.72971 | 1.77775 | 0.318069 | 5.5892 |  |
| 1559 | 0.0129654 | 0.26778 | 0.511168 | 0.178602 | 2.86205 |  |
| 1560 | 0.0605052 | 0.954264 | 1.11911 | 0.338933 | 3.30187 |  |
| 1561 | 0.0009604 | 0.0381041 | 0.213138 | 0.059534 | 3.58011 |  |
| 1562 | 0.0172872 | 0.356099 | 0.545921 | 0.249899 | 2.18457 |  |
| 1563 | 0.0004802 | 0.019606 | 0.059534 | 0.059534 | 1 |  |
| 1564 | 0.132535 | 1.9437 | 1.51279 | 0.298501 | 5.06794 |  |
| 1565 | 0.0004802 | 0.019606 | 0.059534 | 0.059534 | 1 |  |
| 1566 | 0.158466 | 2.06077 | 1.73998 | 0.278102 | 6.25663 |  |
| 1567 | 0.196882 | 2.87209 | 1.65918 | 0.724425 | 2.29034 |  |
| 1568 | 0.0086436 | 0.190465 | 0.37424 | 0.255224 | 1.46632 |  |
| 1569 | 0.0374556 | 0.579477 | 0.670886 | 0.27579 | 2.4326 |  |
| 1570 | 0.0292922 | 0.581641 | 0.841614 | 0.258535 | 3.25532 |  |
| 1571 | 0.115728 | 1.49651 | 1.29518 | 0.471136 | 2.74906 |  |
| 1572 | 0.245862 | 2.49384 | 1.69797 | 0.495838 | 3.42443 |  |
| 1573 | 0.007203 | 0.184349 | 0.426278 | 0.168818 | 2.52507 |  |
| 1574 | 0.0705894 | 1.05288 | 1.17367 | 0.338468 | 3.4676 |  |
| 1575 | 0.0067228 | 0.173214 | 0.403654 | 0.159034 | 2.53816 |  |
| 1576 | 0.0091238 | 0.209478 | 0.426326 | 0.228998 | 1.8617 |  |
| 1577 | 0.021609 | 0.382474 | 0.597669 | 0.258535 | 2.31175 |  |
| 1578 | 0.016807 | 0.329362 | 0.457942 | 0.363151 | 1.26102 |  |
| 1579 | 0.0004802 | 0.019606 | 0.059534 | 0.059534 | 1 |  |
| 1580 | 8.51635 | 48.4529 | 9.9095 | 1.22003 | 8.12235 |  |
| 1581 | 0.0196882 | 0.350774 | 0.528104 | 0.331392 | 1.59359 |  |
| 1582 | 0.0009604 | 0.0381041 | 0.208247 | 0.092508 | 2.25113 |  |
| 1583 | 9.91373 | 38.641 | 5.617 | 1.45004 | 3.87367 |  |
| 1584 | 0.0091238 | 0.213403 | 0.445903 | 0.245202 | 1.81852 |  |
| 1585 | 0.0331338 | 0.565367 | 0.71243 | 0.247919 | 2.87364 |  |
| 1586 | 0.0028812 | 0.0913024 | 0.293123 | 0.166099 | 1.76475 |  |
| 1587 | 0.0009604 | 0.032799 | 0.109284 | 0.143766 | 0.760151 |  |
| 1588 | 0.0153664 | 0.345673 | 0.639707 | 0.259046 | 2.46947 |  |
| 1589 | 0.009604 | 0.230603 | 0.480118 | 0.178602 | 2.6882 |  |
| 1590 | 0.0009604 | 0.0381041 | 0.208247 | 0.092506 | 2.25118 |  |
| 1591 | 0.0307328 | 0.532131 | 0.721829 | 0.299033 | 2.41388 |  |
| 1592 | 0.466754 | 3.71069 | 1.83779 | 0.739343 | 2.4857 |  |
| 1593 | 0.0091238 | 0.218498 | 0.532645 | 0.168818 | 3.15514 |  |
| 1594 | 0.016807 | 0.33624 | 0.573573 | 0.178602 | 3.21147 |  |
| 1595 | 0.0465794 | 0.802209 | 1.06864 | 0.257704 | 4.14679 |  |
| 1596 | 0.064827 | 1.0202 | 1.09577 | 0.349558 | 3.13474 |  |
| 1597 | 0.02401 | 0.46034 | 0.653645 | 0.218568 | 2.99058 |  |
| 1598 | 0.0033614 | 0.0994345 | 0.293121 | 0.191371 | 1.53169 |  |
| 1599 | 0.0028812 | 0.0879606 | 0.318127 | 0.134435 | 2.36641 |  |
| 1600 | 0.0211288 | 0.438703 | 0.635238 | 0.238136 | 2.66754 |  |
| 1601 | 0.004802 | 0.133909 | 0.423218 | 0.128851 | 3.28454 |  |
| 1602 | 3.55012 | 19.3569 | 4.51479 | 1.21108 | 3.72791 |  |
| 1603 | 0.0412972 | 0.688551 | 0.935606 | 0.238136 | 3.92888 |  |
| 1604 | 0.0004802 | 0.019606 | 0.059534 | 0.059534 | 1 |  |
| 1605 | 0.0062426 | 0.161908 | 0.364012 | 0.119068 | 3.05719 |  |
| 1606 | 0.0081634 | 0.198337 | 0.415145 | 0.178602 | 2.32442 |  |
| 1607 | 0.520537 | 5.78995 | 2.69384 | 0.445257 | 6.05008 |  |
| 1608 | 0.242981 | 2.57401 | 1.58272 | 0.395506 | 4.00177 |  |
| 1609 | 0.0100842 | 0.252006 | 0.566551 | 0.159034 | 3.56245 |  |
| 1610 | 0.0014406 | 0.0560173 | 0.279902 | 0.069318 | 4.03796 |  |
| 1611 | 0.0009604 | 0.032799 | 0.109284 | 0.143766 | 0.760152 |  |
| 1612 | 0.139738 | 1.937 | 1.5162 | 0.466487 | 3.25024 |  |
| 1613 | 0.0129654 | 0.316529 | 0.712055 | 0.242247 | 2.93937 |  |
| 1614 | 0.0004802 | 0.019606 | 0.059534 | 0.059534 | 1 |  |
| 1615 | 0.0691488 | 0.949466 | 0.847257 | 0.420695 | 2.01395 |  |
| 1616 | 0.0485002 | 0.741392 | 0.826036 | 0.268319 | 3.07856 |  |
| 1617 | 0.0028812 | 0.092253 | 0.320168 | 0.109284 | 2.92969 |  |
| 1618 | 0.760157 | 7.17539 | 3.50321 | 1.04104 | 3.36509 |  |
| 1619 | 0.0893172 | 1.47538 | 1.96796 | 0.422427 | 4.6587 |  |
| 1620 | 0.397606 | 4.41416 | 2.60291 | 0.605954 | 4.29556 |  |
| 1621 | 0.002401 | 0.0758411 | 0.293119 | 0.119068 | 2.46179 |  |
| 1622 | 0.0038416 | 0.113203 | 0.362054 | 0.119068 | 3.04074 |  |
| 1623 | 0.0019208 | 0.0603798 | 0.219568 | 0.127091 | 1.72764 |  |
| 1624 | 0.002401 | 0.0758411 | 0.285059 | 0.119068 | 2.39409 |  |
| 1625 | 0.0067228 | 0.177797 | 0.449149 | 0.138635 | 3.23979 |  |
| 1626 | 0.0201684 | 0.400875 | 0.702319 | 0.178602 | 3.93232 |  |
| 1627 | 0.0067228 | 0.172492 | 0.423218 | 0.128851 | 3.28455 |  |
| 1628 | 0.243942 | 2.36346 | 1.51294 | 0.546421 | 2.76882 |  |
| 1629 | 0.014406 | 0.268756 | 0.454446 | 0.268318 | 1.69369 |  |
| 1630 | 0.164228 | 1.9605 | 1.52252 | 0.45147 | 3.37235 |  |
| 1631 | 0.0835548 | 1.63653 | 1.51364 | 0.216905 | 6.97836 |  |
| 1632 | 0.004802 | 0.140051 | 0.415142 | 0.128852 | 3.22186 |  |
| 1633 | 0.0081634 | 0.194933 | 0.444861 | 0.178602 | 2.4908 |  |
| 1634 | 0.16807 | 1.8743 | 1.48967 | 0.358035 | 4.16069 |  |
| 1635 | 0.773122 | 6.80659 | 2.85094 | 0.503127 | 5.66645 |  |
| 1636 | 0.0028812 | 0.0848895 | 0.288523 | 0.119068 | 2.42317 |  |
| 1637 | 0.0009604 | 0.032799 | 0.109284 | 0.143766 | 0.760151 |  |
| 1638 | 0.0009604 | 0.032799 | 0.109284 | 0.143766 | 0.760151 |  |
| 1639 | 0.21705 | 3.43598 | 2.09447 | 0.641091 | 3.26705 |  |
| 1640 | 0.064827 | 0.822762 | 0.826245 | 0.412597 | 2.00255 |  |
| 1641 | 0.0825944 | 1.10572 | 1.15487 | 0.404872 | 2.85245 |  |
| 1642 | 0.761117 | 7.55589 | 2.68311 | 0.825042 | 3.25208 |  |
| 1643 | 0.0633864 | 0.978998 | 0.938645 | 0.4452 | 2.10837 |  |
| 1644 | 1.78874 | 14.0383 | 4.70006 | 1.1376 | 4.13156 |  |
| 1645 | 0.009604 | 0.237731 | 0.495942 | 0.138635 | 3.57732 |  |
| 1646 | 0.0139258 | 0.294897 | 0.497665 | 0.178601 | 2.78646 |  |
| 1647 | 0.0633864 | 1.03767 | 1.27813 | 0.268319 | 4.76347 |  |
| 1648 | 0.0009604 | 0.032799 | 0.109284 | 0.143766 | 0.760151 |  |
| 1649 | 0.0182476 | 0.358524 | 0.627591 | 0.208784 | 3.00593 |  |
| 1650 | 0.0033614 | 0.105634 | 0.304721 | 0.205168 | 1.48523 |  |
| 1651 | 0.0081634 | 0.20454 | 0.498648 | 0.168818 | 2.95376 |  |
| 1652 | 0.02401 | 0.424235 | 0.673735 | 0.274709 | 2.45254 |  |
| 1653 | 0.0115248 | 0.253733 | 0.447 | 0.168818 | 2.64782 |  |
| 1654 | 0.725102 | 7.91321 | 3.52168 | 0.954672 | 3.68889 |  |
| 1655 | 0.151263 | 2.08728 | 1.61444 | 0.317237 | 5.08908 |  |
| 1656 | 0.0211288 | 0.378304 | 0.596188 | 0.292788 | 2.03624 |  |
| 1657 | 0.0009604 | 0.0381041 | 0.213139 | 0.059534 | 3.58013 |  |
| 1658 | 0.004802 | 0.133996 | 0.361977 | 0.128852 | 2.80924 |  |
| 1659 | 0.0033614 | 0.103422 | 0.360254 | 0.119068 | 3.02562 |  |
| 1660 | 0.0019208 | 0.0603798 | 0.219568 | 0.127085 | 1.72772 |  |
| 1661 | 1.10158 | 9.93092 | 3.85206 | 0.882392 | 4.36548 |  |
| 1662 | 0.0758716 | 1.08798 | 1.01064 | 0.352955 | 2.86336 |  |
| 1663 | 0.0052822 | 0.141534 | 0.33362 | 0.208443 | 1.60054 |  |
| 1664 | 1.52944 | 18.2934 | 7.10713 | 0.733974 | 9.68309 |  |
| 1665 | 0.012005 | 0.266394 | 0.435763 | 0.178602 | 2.43986 |  |
| 1666 | 0.0019208 | 0.0662077 | 0.275415 | 0.143737 | 1.9161 |  |
| 1667 | 0.0595448 | 1.24017 | 1.63836 | 0.178601 | 9.17328 |  |
| 1668 | 0.0297724 | 0.562301 | 0.766074 | 0.267948 | 2.85904 |  |
| 1669 | 0.0537824 | 0.771925 | 0.844078 | 0.367819 | 2.29482 |  |
| 1670 | 0.206966 | 3.59154 | 2.53484 | 0.366155 | 6.92286 |  |
| 1671 | 0.289561 | 2.71525 | 1.45381 | 0.396338 | 3.6681 |  |
| 1672 | 0.0067228 | 0.205833 | 0.599599 | 0.182925 | 3.27785 |  |
| 1673 | 0.0052822 | 0.142616 | 0.361977 | 0.128851 | 2.80926 |  |
| 1674 | 0.0038416 | 0.113394 | 0.352352 | 0.191371 | 1.8412 |  |
| 1675 | 0.074431 | 0.944028 | 0.879055 | 0.327852 | 2.68125 |  |
| 1676 | 0.0067228 | 0.203398 | 0.642774 | 0.188175 | 3.41583 |  |
| 1677 | 0.0009604 | 0.032799 | 0.109284 | 0.143766 | 0.760153 |  |
| 1678 | 0.549349 | 9.13615 | 4.65494 | 0.540597 | 8.61073 |  |
| 1679 | 0.02401 | 0.470687 | 0.710671 | 0.198169 | 3.58619 |  |
| 1680 | 0.0033614 | 0.105656 | 0.359161 | 0.119068 | 3.01644 |  |
| 1681 | 0.0038416 | 0.113203 | 0.362054 | 0.119068 | 3.04073 |  |
| 1682 | 0.0052822 | 0.153765 | 0.413381 | 0.189882 | 2.17704 |  |
| 1683 | 0.0436982 | 0.706394 | 0.794426 | 0.247919 | 3.20438 |  |
| 1684 | 0.166629 | 2.72558 | 2.27089 | 0.515156 | 4.40816 |  |
| 1685 | 0.0297724 | 0.575096 | 0.916161 | 0.217737 | 4.20766 |  |
| 1686 | 0.009604 | 0.233229 | 0.489625 | 0.168818 | 2.90031 |  |
| 1687 | 0.0057624 | 0.149927 | 0.360911 | 0.168818 | 2.13787 |  |
| 1688 | 0.0019208 | 0.0603798 | 0.219568 | 0.127083 | 1.72775 |  |
| 1689 | 0.0100842 | 0.23567 | 0.491903 | 0.178602 | 2.75419 |  |
| 1690 | 0.31309 | 3.89286 | 2.44743 | 0.630862 | 3.87949 |  |
| 1691 | 0.332779 | 3.72379 | 2.2436 | 0.457535 | 4.90367 |  |
| 1692 | 0.0052822 | 0.151481 | 0.415612 | 0.128852 | 3.22551 |  |
| 1693 | 0.0052822 | 0.160931 | 0.416553 | 0.119068 | 3.49845 |  |
| 1694 | 0.0835548 | 1.49007 | 1.65174 | 0.207953 | 7.94284 |  |
| 1695 | 0.0004802 | 0.019606 | 0.059534 | 0.059534 | 1 |  |
| 1696 | 0.199763 | 2.39847 | 1.8754 | 0.27727 | 6.76381 |  |
| 1697 | 0.0009604 | 0.032799 | 0.109284 | 0.143766 | 0.760153 |  |
| 1698 | 0.118609 | 1.75161 | 1.52271 | 0.401011 | 3.79717 |  |
| 1699 | 0.0028812 | 0.0984743 | 0.352348 | 0.137894 | 2.5552 |  |
| 1700 | 0.004802 | 0.136177 | 0.359161 | 0.128852 | 2.7874 |  |
| 1701 | 1.31287 | 11.2017 | 3.45822 | 0.503959 | 6.86212 |  |
| 1702 | 0.012005 | 0.283784 | 0.511135 | 0.159035 | 3.21398 |  |
| 1703 | 0.002401 | 0.0820625 | 0.286624 | 0.137894 | 2.07858 |  |
| 1704 | 0.0211288 | 0.458168 | 0.71243 | 0.138635 | 5.13888 |  |
| 1705 | 0.0172872 | 0.390215 | 0.704696 | 0.158202 | 4.45439 |  |
| 1706 | 0.0513814 | 0.908404 | 1.0178 | 0.29767 | 3.41921 |  |
| 1707 | 0.0801934 | 1.29784 | 1.58548 | 0.306622 | 5.17082 |  |
| 1708 | 0.508052 | 7.71593 | 3.65899 | 0.621363 | 5.88864 |  |
| 1709 | 0.0595448 | 0.997855 | 1.0625 | 0.34497 | 3.07997 |  |
| 1710 | 0.352947 | 4.348 | 2.60351 | 0.37677 | 6.91007 |  |
| 1711 | 0.116208 | 1.52045 | 1.16728 | 0.307453 | 3.7966 |  |
| 1712 | 0.0076832 | 0.225864 | 0.794412 | 0.109284 | 7.26922 |  |
| 1713 | 0.0388962 | 0.589742 | 0.730835 | 0.327852 | 2.22916 |  |
| 1714 | 0.291481 | 4.12752 | 2.52035 | 0.308285 | 8.17539 |  |
| 1715 | 0.0710696 | 1.40202 | 1.20029 | 0.207121 | 5.79513 |  |
| 1716 | 0.0379358 | 0.723299 | 0.996544 | 0.294335 | 3.38575 |  |
| 1717 | 0.0806736 | 1.17286 | 1.21831 | 0.34742 | 3.50674 |  |
| 1718 | 0.012005 | 0.257623 | 0.450859 | 0.272223 | 1.65621 |  |
| 1719 | 0.176233 | 2.44074 | 1.58646 | 0.366155 | 4.33274 |  |
| 1720 | 0.146461 | 2.4294 | 1.60538 | 0.40529 | 3.96107 |  |
| 1721 | 0.0485002 | 0.845234 | 1.04677 | 0.327021 | 3.20094 |  |
| 1722 | 0.043218 | 0.825737 | 1.02403 | 0.317237 | 3.22795 |  |
| 1723 | 0.139258 | 2.49252 | 2.18271 | 0.307453 | 7.09934 |  |
| 1724 | 0.0331338 | 0.790062 | 1.42139 | 0.324961 | 4.37403 |  |
| 1725 | 0.0869162 | 1.67595 | 1.66779 | 0.238967 | 6.97915 |  |
| 1726 | 0.202644 | 2.57248 | 1.73093 | 0.29767 | 5.81494 |  |
| 1727 | 0.0379358 | 0.660219 | 0.820758 | 0.277516 | 2.95752 |  |
| 1728 | 0.0009604 | 0.032799 | 0.109284 | 0.143766 | 0.760151 |  |
| 1729 | 0.004802 | 0.13698 | 0.429406 | 0.119068 | 3.60639 |  |
| 1730 | 0.883568 | 9.75291 | 4.06713 | 0.35554 | 11.4393 |  |
| 1731 | 0.0230496 | 0.449878 | 0.627592 | 0.228352 | 2.74835 |  |
| 1732 | 0.0067228 | 0.189582 | 0.613631 | 0.142395 | 4.30934 |  |
| 1733 | 0.0033614 | 0.101886 | 0.361977 | 0.128851 | 2.80926 |  |
| 1734 | 0.0393764 | 0.704074 | 0.956602 | 0.267487 | 3.57626 |  |
| 1735 | 0.0091238 | 0.227103 | 0.449157 | 0.178602 | 2.51485 |  |
| 1736 | 0.0172872 | 0.422767 | 0.791119 | 0.158202 | 5.00069 |  |
| 1737 | 0.012005 | 0.399169 | 0.62759 | 0.138635 | 4.52693 |  |
| 1738 | 0.0086436 | 0.268602 | 0.567295 | 0.128851 | 4.40271 |  |
| 1739 | 0.004802 | 0.164874 | 0.426044 | 0.119068 | 3.57816 |  |
| 1740 | 0.02401 | 0.798215 | 1.18916 | 0.147587 | 8.05736 |  |
| 1741 | 0.0172872 | 0.445046 | 0.695064 | 0.148419 | 4.68313 |  |
| 1742 | 0.0182476 | 0.460298 | 0.81047 | 0.138635 | 5.84607 |  |
| 1743 | 0.0076832 | 0.225217 | 0.497661 | 0.138635 | 3.58972 |  |
| 1744 | 0.0043218 | 0.146011 | 0.416497 | 0.119068 | 3.49799 |  |
| 1745 | 0.031213 | 0.731742 | 0.98002 | 0.187554 | 5.22528 |  |
| 1746 | 0.0067228 | 0.204295 | 0.426044 | 0.128851 | 3.30647 |  |
| 1747 | 0.0043218 | 0.146011 | 0.411103 | 0.119068 | 3.45269 |  |
| 1748 | 0.0921984 | 2.05652 | 2.5559 | 0.187553 | 13.6276 |  |
| 1749 | 0.0115248 | 0.33994 | 0.689683 | 0.138635 | 4.9748 |  |
| 1750 | 0.0091238 | 0.273951 | 0.594168 | 0.119068 | 4.99015 |  |
| 1751 | 0.0196882 | 0.574791 | 1.14226 | 0.158203 | 7.22025 |  |
| 1752 | 0.036015 | 0.830948 | 0.98002 | 0.187554 | 5.22528 |  |
| 1753 | 0.0139258 | 0.346361 | 0.614015 | 0.148419 | 4.13704 |  |
| 1754 | 0.0086436 | 0.256341 | 0.52003 | 0.138635 | 3.75106 |  |
| 1755 | 0.0172872 | 0.470051 | 0.822177 | 0.158202 | 5.19699 |  |
| 1756 | 0.0398566 | 0.928736 | 1.22601 | 0.158202 | 7.74967 |  |
| 1757 | 0.0067228 | 0.219565 | 0.511811 | 0.128851 | 3.9721 |  |
| 1758 | 0.0110446 | 0.344799 | 0.901703 | 0.148419 | 6.0754 |  |
| 1759 | 0.0081634 | 0.253332 | 0.572703 | 0.128851 | 4.44469 |  |
| 1760 | 0.0043218 | 0.128079 | 0.352352 | 0.119068 | 2.95925 |  |
| 1761 | 0.0028812 | 0.0913024 | 0.308397 | 0.119068 | 2.59009 |  |
| 1762 | 0.0004802 | 0.019606 | 0.059534 | 0.059534 | 1 |  |
| 1763 | 0.0019208 | 0.0739304 | 0.308396 | 0.069318 | 4.449 |  |
| 1764 | 0.0076832 | 0.283287 | 0.840768 | 0.118236 | 7.11093 |  |
| 1765 | 0.0009604 | 0.0381041 | 0.208249 | 0.092507 | 2.25118 |  |
| 1766 | 0.0076832 | 0.278209 | 0.601484 | 0.108452 | 5.54609 |  |
| 1767 | 0.0014406 | 0.0566022 | 0.304721 | 0.09391 | 3.24481 |  |
| 1768 | 0.0019208 | 0.0728226 | 0.279904 | 0.069318 | 4.03799 |  |
| 1769 | 0.0009604 | 0.0381041 | 0.213139 | 0.059534 | 3.58012 |  |
| 1770 | 0.0004802 | 0.019606 | 0.059534 | 0.059534 | 1 |  |
| 1771 | 0.0028812 | 0.107541 | 0.356025 | 0.079101 | 4.50087 |  |
| 1772 | 0.0028812 | 0.108064 | 0.356028 | 0.079101 | 4.50092 |  |
| 1773 | 0.0158466 | 0.562481 | 0.915972 | 0.108452 | 8.44585 |  |
| 1774 | 0.0009604 | 0.0381041 | 0.213139 | 0.059534 | 3.58013 |  |
| 1775 | 0.0019208 | 0.0728226 | 0.279904 | 0.069318 | 4.03796 |  |
| 1776 | 0.0009604 | 0.0381041 | 0.213139 | 0.059534 | 3.58013 |  |
| 1777 | 0.0043218 | 0.159588 | 0.491929 | 0.079101 | 6.21898 |  |
| 1778 | 0.0009604 | 0.0381041 | 0.213139 | 0.059534 | 3.58012 |  |
| 1779 | 0.0081634 | 0.296707 | 0.702319 | 0.088886 | 7.90139 |  |
| 1780 | 0.0004802 | 0.019606 | 0.059534 | 0.059534 | 1 |  |
| 1781 | 0.0057624 | 0.210527 | 0.572703 | 0.079101 | 7.24012 |  |
| 1782 | 0.0009604 | 0.0381041 | 0.213137 | 0.059534 | 3.58009 |  |
| 1783 | 0.0004802 | 0.019606 | 0.059534 | 0.059534 | 1 |  |
| 1784 | 0.0009604 | 0.0381041 | 0.213139 | 0.059534 | 3.58013 |  |
| 1785 | 0.0028812 | 0.108064 | 0.356028 | 0.079101 | 4.50092 |  |
| 1786 | 0.0355348 | 1.24731 | 1.59894 | 0.167154 | 9.56564 |  |
| 1787 | 0.0014406 | 0.0566022 | 0.308397 | 0.059534 | 5.18019 |  |
| 1788 | 0.0004802 | 0.019606 | 0.059534 | 0.059534 | 1 |  |
| 1789 | 0.0009604 | 0.0381041 | 0.213139 | 0.059534 | 3.58013 |  |
| 1790 | 0.0028812 | 0.108649 | 0.416545 | 0.069318 | 6.00918 |  |
| 1791 | 0.0009604 | 0.0381041 | 0.213139 | 0.059534 | 3.58013 |  |
| 1792 | 0.0028812 | 0.108064 | 0.352348 | 0.079101 | 4.45439 |  |
| Mean | 25.4824 | 24.4595 | 1.17471 | 0.331788 | 3.32606 |  |
|  |  |  |  |  |  |  |
