# Supplementary material for: Investigation of Three-Dimensional Microstructure of Tricalcium Silicate (C3S) by Electron Microscopy
Source: Materials (Basel). 2018 Jun 29;11(7):1110. doi: 10.3390/ma11071110 (PMC6073500; doi:10.3390/ma11071110)
Supplement: Supplementary file 1 [file materials-11-01110-s001.zip › Supplementary Spreadsheet/Supplementary Spreadsheet S2_proofread.docx]

　　Spreadsheet S2: Areas of C_3_S and pores per slice image and the porosity per slice

| index | C_3_S (μm^2^) | Pore (μm^2^) | Porosity (%)%) |
| --- | --- | --- | --- |
| 1 | 2852.09 | 50.7283 | 1.747553 |
| 2 | 2844.09 | 47.463 | 1.641436 |
| 3 | 2825.21 | 47.5398 | 1.654853 |
| 4 | 2823.7 | 47.1748 | 1.64322 |
| 5 | 2850.68 | 45.2733 | 1.56333 |
| 6 | 2866.44 | 43.8038 | 1.505159 |
| 7 | 2909.57 | 39.6549 | 1.344587 |
| 8 | 2957.59 | 36.2071 | 1.209404 |
| 9 | 2973 | 235.519 | 7.340427 |
| 10 | 2984.6 | 236.306 | 7.336631 |
| 11 | 2974.31 | 235.164 | 7.327182 |
| 12 | 2963.59 | 230.266 | 7.209655 |
| 13 | 2959.17 | 228.662 | 7.172963 |
| 14 | 2961.79 | 226.962 | 7.117581 |
| 15 | 2986.61 | 224.849 | 7.001459 |
| 16 | 3009.77 | 222.64 | 6.88774 |
| 17 | 3021.89 | 220.258 | 6.793583 |
| 18 | 3076.07 | 219.864 | 6.670765 |
| 19 | 3084.56 | 218.75 | 6.622146 |
| 20 | 3084.24 | 217.924 | 6.59943 |
| 21 | 3090.39 | 216.993 | 6.560867 |
| 22 | 3095.05 | 213.497 | 6.452893 |
| 23 | 3092.76 | 213.91 | 6.469046 |
| 24 | 3077.9 | 214.371 | 6.511341 |
| 25 | 3084.55 | 213.91 | 6.485148 |
| 26 | 3076.85 | 214.918 | 6.528953 |
| 27 | 3081.25 | 217.29 | 6.58746 |
| 28 | 3096.25 | 216.08 | 6.523505 |
| 29 | 3092.42 | 215.994 | 6.528627 |
| 30 | 3160.12 | 237.488 | 6.989859 |
| 31 | 3155.86 | 240.686 | 7.086199 |
| 32 | 3181.38 | 242.088 | 7.071426 |
| 33 | 3207.08 | 241.08 | 6.991555 |
| 34 | 3220.37 | 294.487 | 8.378349 |
| 35 | 3186.39 | 323.146 | 9.207656 |
| 36 | 3187.38 | 334.286 | 9.492269 |
| 37 | 3177.33 | 339.962 | 9.665447 |
| 38 | 3147.44 | 341.24 | 9.78135 |
| 39 | 3135.21 | 345.389 | 9.923263 |
| 40 | 3113.51 | 343.333 | 9.931981 |
| 41 | 3126.15 | 335.439 | 9.690319 |
| 42 | 3133.96 | 335.324 | 9.66551 |
| 43 | 3148.74 | 336.745 | 9.661353 |
| 44 | 3143.22 | 338.397 | 9.719535 |
| 45 | 3153.84 | 335.919 | 9.625851 |
| 46 | 3174.9 | 343.996 | 9.77568 |
| 47 | 3214.19 | 365.912 | 10.22071 |
| 48 | 3228.68 | 369.744 | 10.27516 |
| 49 | 3181.47 | 372.395 | 10.47859 |
| 50 | 3205.45 | 372.702 | 10.41605 |
| 51 | 3220.92 | 371.319 | 10.3367 |
| 52 | 3227.3 | 370.705 | 10.30307 |
| 53 | 3229.2 | 370.686 | 10.29716 |
| 54 | 3225.22 | 370.858 | 10.31285 |
| 55 | 3237.17 | 368.323 | 10.21561 |
| 56 | 3246.09 | 367.68 | 10.17442 |
| 57 | 3234.98 | 364.212 | 10.11927 |
| 58 | 3265.07 | 363.367 | 10.01442 |
| 59 | 3252.14 | 368.525 | 10.17838 |
| 60 | 3269.72 | 375.488 | 10.30087 |
| 61 | 3271.74 | 379.195 | 10.38624 |
| 62 | 3279.26 | 379.358 | 10.36889 |
| 63 | 3291.77 | 378.705 | 10.3176 |
| 64 | 3355.11 | 383.93 | 10.26814 |
| 65 | 3368.65 | 389.903 | 10.37375 |
| 66 | 3355.74 | 391.334 | 10.44372 |
| 67 | 3352.43 | 392.621 | 10.48373 |
| 68 | 3354.85 | 391.891 | 10.45952 |
| 69 | 3347.32 | 391.142 | 10.46264 |
| 70 | 3290.35 | 380.914 | 10.37555 |
| 71 | 3299.2 | 377.831 | 10.27544 |
| 72 | 3324.78 | 373.394 | 10.09671 |
| 73 | 3330.89 | 370.714 | 10.01496 |
| 74 | 3358.7 | 371.415 | 9.9572 |
| 75 | 3357.97 | 370.013 | 9.925287 |
| 76 | 3342.4 | 369.658 | 9.958303 |
| 77 | 3369.78 | 370.446 | 9.904375 |
| 78 | 3382.11 | 370.609 | 9.875746 |
| 79 | 3399.91 | 372.837 | 9.882375 |
| 80 | 3389.23 | 455.911 | 11.85681 |
| 81 | 3359.46 | 451.743 | 11.85303 |
| 82 | 3332.76 | 448.219 | 11.85458 |
| 83 | 3326.98 | 445.827 | 11.81685 |
| 84 | 3356.14 | 442.562 | 11.65035 |
| 85 | 3346.37 | 440.622 | 11.63514 |
| 86 | 3451.42 | 431.873 | 11.12131 |
| 87 | 3510.51 | 424.554 | 10.789 |
| 88 | 3548.62 | 435.224 | 10.92472 |
| 89 | 3520.93 | 440.295 | 11.11512 |
| 90 | 3531.14 | 439.69 | 11.073 |
| 91 | 3559.65 | 436.636 | 10.92604 |
| 92 | 3553.39 | 434.034 | 10.88507 |
| 93 | 3540.5 | 433.208 | 10.90186 |
| 94 | 3522.87 | 433.736 | 10.96232 |
| 95 | 3532.86 | 435.57 | 10.97588 |
| 96 | 3511.75 | 436.867 | 11.0638 |
| 97 | 3523.9 | 434.101 | 10.96768 |
| 98 | 3594.85 | 428.175 | 10.64311 |
| 99 | 3582.66 | 422.49 | 10.54867 |
| 100 | 3561.17 | 418.715 | 10.52078 |
| 101 | 3597.59 | 415.555 | 10.35485 |
| 102 | 3566 | 413.471 | 10.3901 |
| 103 | 3574.46 | 412.088 | 10.33696 |
| 104 | 3587.32 | 413.923 | 10.34486 |
| 105 | 3614.96 | 435.071 | 10.74241 |
| 106 | 3644.16 | 439.268 | 10.75733 |
| 107 | 3689.08 | 441.419 | 10.68682 |
| 108 | 3662.91 | 441.16 | 10.74933 |
| 109 | 3656.91 | 444.464 | 10.83695 |
| 110 | 3627.42 | 446.807 | 10.96667 |
| 111 | 3629.06 | 447.998 | 10.98827 |
| 112 | 3630.88 | 448.564 | 10.99571 |
| 113 | 3625.1 | 447.383 | 10.98551 |
| 114 | 3643.23 | 448.468 | 10.96044 |
| 115 | 3643.39 | 448.997 | 10.97152 |
| 116 | 3674.14 | 448.843 | 10.88637 |
| 117 | 3686.75 | 445.357 | 10.77796 |
| 118 | 3758.97 | 440.507 | 10.48957 |
| 119 | 3686.06 | 434.005 | 10.53394 |
| 120 | 3782.58 | 431.335 | 10.23597 |
| 121 | 3811.8 | 424.045 | 10.01087 |
| 122 | 3771.94 | 433.611 | 10.31044 |
| 123 | 3781.5 | 444.982 | 10.52843 |
| 124 | 3802.26 | 444.185 | 10.46016 |
| 125 | 3815.65 | 442.437 | 10.39051 |
| 126 | 3807.27 | 440.238 | 10.36462 |
| 127 | 3768.72 | 437.77 | 10.40701 |
| 128 | 3781.57 | 433.525 | 10.28506 |
| 129 | 3802.43 | 426.955 | 10.09497 |
| 130 | 3826.34 | 420.098 | 9.89295 |
| 131 | 3838.31 | 410.753 | 9.666908 |
| 132 | 3841.72 | 411.579 | 9.6767 |
| 133 | 3854 | 407.978 | 9.572504 |
| 134 | 3939.38 | 386.916 | 8.943355 |
| 135 | 3964.01 | 375.612 | 8.655408 |
| 136 | 3940.11 | 372.664 | 8.640935 |
| 137 | 3942.12 | 370.657 | 8.594393 |
| 138 | 3947.2 | 370.321 | 8.577167 |
| 139 | 3915.54 | 368.976 | 8.611848 |
| 140 | 3943.19 | 365.384 | 8.480393 |
| 141 | 3995.5 | 358.248 | 8.228496 |
| 142 | 3989.4 | 345.754 | 7.975587 |
| 143 | 4014.64 | 342.21 | 7.854528 |
| 144 | 3997.12 | 342.748 | 7.89766 |
| 145 | 3948.19 | 343.401 | 8.001718 |
| 146 | 3939.22 | 343.189 | 8.013924 |
| 147 | 3991.18 | 337.283 | 7.792212 |
| 148 | 4002.48 | 333.931 | 7.700631 |
| 149 | 4051.56 | 320.966 | 7.340517 |
| 150 | 4183.76 | 281.983 | 6.314358 |
| 151 | 4214.83 | 266.607 | 5.949141 |
| 152 | 4213.02 | 266.184 | 5.942663 |
| 153 | 4221.3 | 265.234 | 5.91178 |
| 154 | 4219.68 | 264.946 | 5.907873 |
| 155 | 4224.4 | 263.851 | 5.878704 |
| 156 | 4214.5 | 262.89 | 5.871501 |
| 157 | 4196.5 | 262.132 | 5.879202 |
| 158 | 4214.95 | 256.955 | 5.745985 |
| 159 | 4247.01 | 247.822 | 5.513487 |
| 160 | 4303.36 | 224.724 | 4.962894 |
| 161 | 4383.98 | 155.815 | 3.432203 |
| 162 | 4343.66 | 158.696 | 3.524732 |
| 163 | 4363.39 | 161.059 | 3.559748 |
| 164 | 4325.28 | 162.288 | 3.616391 |
| 165 | 4345.43 | 163.326 | 3.622418 |
| 166 | 4306.5 | 169.136 | 3.779038 |
| 167 | 4341.01 | 169.876 | 3.765912 |
| 168 | 4335.44 | 167.273 | 3.714938 |
| 169 | 4354.71 | 161.923 | 3.585038 |
| 170 | 4339.36 | 174.822 | 3.872728 |
| 171 | 4342.72 | 180.574 | 3.992091 |
| 172 | 4330.38 | 187.71 | 4.154632 |
| 173 | 4328.69 | 192.743 | 4.262874 |
| 174 | 4323.66 | 195.038 | 4.316243 |
| 175 | 4315.25 | 198.082 | 4.38882 |
| 176 | 4322.62 | 200.752 | 4.438105 |
| 177 | 4318.22 | 201.031 | 4.448326 |
| 178 | 4315.13 | 200.906 | 4.448725 |
| 179 | 4315.72 | 197.554 | 4.377177 |
| 180 | 4333.41 | 196.92 | 4.346703 |
| 181 | 4358.4 | 189.458 | 4.165873 |
| 182 | 4206.67 | 209.694 | 4.748114 |
| 183 | 4172.2 | 217.406 | 4.952745 |
| 184 | 4150.78 | 226.203 | 5.168012 |
| 185 | 4147.03 | 235.432 | 5.37214 |
| 186 | 4040.08 | 233.819 | 5.470859 |
| 187 | 4042.02 | 237.603 | 5.551961 |
| 188 | 4044.61 | 246.852 | 5.752166 |
| 189 | 4038.91 | 249.81 | 5.824815 |
| 190 | 4051.2 | 255.073 | 5.923289 |
| 191 | 4028.77 | 252.758 | 5.903453 |
| 192 | 4006.41 | 270.314 | 6.320586 |
| 193 | 4007.49 | 267.923 | 6.266599 |
| 194 | 4039.52 | 258.722 | 6.019252 |
| 195 | 4090.02 | 260.422 | 5.986104 |
| 196 | 4076.55 | 271.303 | 6.23993 |
| 197 | 4053.24 | 277.431 | 6.40619 |
| 198 | 4029.54 | 278.43 | 6.463137 |
| 199 | 4019.76 | 277.056 | 6.447937 |
| 200 | 4005.95 | 275.472 | 6.434124 |
|  |  |  |  |
