# Supplementary material for: Investigation of Three-Dimensional Microstructure of Tricalcium Silicate (C3S) by Electron Microscopy
Source: Materials (Basel). 2018 Jun 29;11(7):1110. doi: 10.3390/ma11071110 (PMC6073500; doi:10.3390/ma11071110)
Supplement: Supplementary file 1 [file materials-11-01110-s001.zip › Supplementary Spreadsheet/Supplementary Spreadsheet S3_proofread.docx]

Spreadsheet S3: Volumes, areas, lengths, widths and length-width ratios of pores within the largest C_3_S grain

| index | Volume(μm^3^)3) | Area3d (μm^2^) | Length (μm)μm) | Width (μm)μm) m) | Length-width ratio | |  |
| --- | --- | --- | --- | --- | --- | --- | --- |
| 1 | 0.102763 | 3.54543 | 2.25585 | 0.225857 | 9.98796 |  |  |
| 2 | 0.002401 | 0.0918436 | 0.411103 | 0.0791013 | 5.19718 |  |  |
| 3 | 0.0907578 | 3.1767 | 2.98431 | 0.333477 | 8.94909 |  |  |
| 4 | 53.8323 | 135.324 | 12.3207 | 3.52561 | 3.49462 |  |  |
| 5 | 0.0076832 | 0.281071 | 0.690606 | 0.118236 | 5.84092 |  |  |
| 6 | 0.021609 | 0.487658 | 0.782243 | 0.168818 | 4.63365 |  |  |
| 7 | 0.297724 | 4.29911 | 2.23841 | 0.326189 | 6.86232 |  |  |
| 8 | 0.207446 | 3.34984 | 2.91743 | 0.296837 | 9.82837 |  |  |
| 9 | 0.009604 | 0.345953 | 0.708391 | 0.0986684 | 7.17951 |  |  |
| 10 | 0.0067228 | 0.242967 | 0.491903 | 0.088885 | 5.53415 |  |  |
| 11 | 0.0038416 | 0.130156 | 0.411104 | 0.119068 | 3.45269 |  |  |
| 12 | 0.0004802 | 0.019606 | 0.0595339 | 0.0595339 | 1 |  |  |
| 13 | 0.275635 | 3.51977 | 2.61575 | 0.407786 | 6.41451 |  |  |
| 14 | 0.0057624 | 0.187483 | 0.416545 | 0.128852 | 3.23275 |  |  |
| 15 | 0.0749112 | 1.16831 | 1.42889 | 0.278102 | 5.13801 |  |  |
| 16 | 0.019208 | 0.384752 | 0.636774 | 0.316394 | 2.0126 |  |  |
| 17 | 0.379358 | 3.92954 | 3.07292 | 0.523526 | 5.86967 |  |  |
| 18 | 0.0028812 | 0.108064 | 0.356027 | 0.0791013 | 4.50091 |  |  |
| 19 | 0.026411 | 0.695864 | 1.09624 | 0.158203 | 6.92933 |  |  |
| 20 | 0.0004802 | 0.019606 | 0.0595339 | 0.0595339 | 1 |  |  |
| 21 | 0.522938 | 4.49277 | 2.21414 | 0.425689 | 5.20131 |  |  |
| 22 | 0.0009604 | 0.0381041 | 0.208249 | 0.0925057 | 2.2512 |  |  |
| 23 | 0.102763 | 1.46843 | 1.6041 | 0.268319 | 5.97835 |  |  |
| 24 | 0.0009604 | 0.0381041 | 0.213139 | 0.0595339 | 3.58013 |  |  |
| 25 | 0.002401 | 0.0820625 | 0.28506 | 0.153508 | 1.85697 |  |  |
| 26 | 0.14358 | 2.55869 | 1.85831 | 0.207953 | 8.9362 |  |  |
| 27 | 0.0225694 | 0.792951 | 0.875067 | 0.12802 | 6.83541 |  |  |
| 28 | 2393.43 | 2938.56 | 69.8049 | 11.7971 | 5.91712 |  |  |
| 29 | 0.0773122 | 0.976877 | 0.983859 | 0.466633 | 2.10842 |  |  |
| 30 | 0.382719 | 4.69302 | 2.93632 | 0.424025 | 6.92488 |  |  |
| 31 | 0.0345744 | 0.59292 | 0.811133 | 0.341592 | 2.37456 |  |  |
| 32 | 0.248744 | 2.2752 | 1.51626 | 0.647028 | 2.34342 |  |  |
| 33 | 1.01994 | 8.55125 | 3.29842 | 0.915071 | 3.60455 |  |  |
| 34 | 0.487403 | 6.25975 | 3.73997 | 0.860614 | 4.34569 |  |  |
| 35 | 0.0129654 | 0.26358 | 0.449157 | 0.283064 | 1.58677 |  |  |
| 36 | 0.0710696 | 1.06051 | 1.40035 | 0.443433 | 3.15798 |  |  |
| 37 | 0.0451388 | 0.784585 | 0.925134 | 0.288718 | 3.20428 |  |  |
| 38 | 0.127733 | 1.59516 | 1.29325 | 0.377603 | 3.42489 |  |  |
| 39 | 0.0019208 | 0.0662077 | 0.279906 | 0.161794 | 1.73002 |  |  |
| 40 | 0.0115248 | 0.245568 | 0.461466 | 0.331943 | 1.3902 |  |  |
| 41 | 0.004802 | 0.144885 | 0.426326 | 0.119068 | 3.58054 |  |  |
| 42 | 0.106604 | 2.04518 | 2.65849 | 0.484205 | 5.49041 |  |  |
| 43 | 0.060025 | 0.889772 | 1.12092 | 0.268318 | 4.17757 |  |  |
| 44 | 0.0797132 | 1.28013 | 1.0081 | 0.649465 | 1.5522 |  |  |
| 45 | 0.0043218 | 0.122251 | 0.364012 | 0.128852 | 2.82505 |  |  |
| 46 | 0.016807 | 0.380032 | 0.74004 | 0.168818 | 4.38365 |  |  |
| 47 | 0.0134456 | 0.279847 | 0.497697 | 0.260864 | 1.90788 |  |  |
| 48 | 0.0220892 | 0.451178 | 0.751238 | 0.265776 | 2.82658 |  |  |
| 49 | 0.0028812 | 0.108064 | 0.352348 | 0.0791012 | 4.4544 |  |  |
| 50 | 0.0009604 | 0.0381041 | 0.213139 | 0.0595339 | 3.58013 |  |  |
| 51 | 0.0393764 | 0.666415 | 0.844078 | 0.218568 | 3.86186 |  |  |
| 52 | 0.155585 | 3.86883 | 3.11981 | 0.356371 | 8.75439 |  |  |
| 53 | 0.406729 | 3.87697 | 1.67933 | 0.795172 | 2.1119 |  |  |
| 54 | 0.0009604 | 0.0381041 | 0.213139 | 0.0595339 | 3.58013 |  |  |
| 55 | 0.0364952 | 0.801187 | 1.43865 | 0.208784 | 6.89062 |  |  |
| 56 | 0.116689 | 1.64136 | 1.56345 | 0.458368 | 3.4109 |  |  |
| 57 | 0.0081634 | 0.220576 | 0.498912 | 0.159034 | 3.13713 |  |  |
| 58 | 0.0009604 | 0.0381041 | 0.208251 | 0.0925057 | 2.25122 |  |  |
| 59 | 0.0028812 | 0.0848895 | 0.288523 | 0.119068 | 2.42317 |  |  |
| 60 | 0.0057624 | 0.166577 | 0.429407 | 0.128851 | 3.33258 |  |  |
| 61 | 0.141179 | 1.81783 | 1.81782 | 0.465876 | 3.90194 |  |  |
| 62 | 0.0364952 | 0.617146 | 0.721825 | 0.311409 | 2.31793 |  |  |
| 63 | 0.154144 | 2.16358 | 1.55651 | 0.475439 | 3.27383 |  |  |
| 64 | 0.0393764 | 0.650473 | 0.915976 | 0.371316 | 2.46684 |  |  |
| 65 | 0.0033614 | 0.0977417 | 0.293119 | 0.119068 | 2.46178 |  |  |
| 66 | 0.0043218 | 0.160696 | 0.497665 | 0.0986687 | 5.0438 |  |  |
| 67 | 0.105644 | 1.56828 | 1.94724 | 0.278102 | 7.0019 |  |  |
| 68 | 0.0004802 | 0.019606 | 0.0595339 | 0.0595339 | 1 |  |  |
| 69 | 3.565 | 32.7656 | 7.37877 | 1.90259 | 3.87828 |  |  |
| 70 | 0.016807 | 0.447934 | 0.975197 | 0.168818 | 5.77662 |  |  |
| 71 | 0.0004802 | 0.019606 | 0.0595339 | 0.0595339 | 1 |  |  |
| 72 | 0.0028812 | 0.107541 | 0.356215 | 0.0693176 | 5.13889 |  |  |
| 73 | 2.56139 | 28.3993 | 7.67374 | 0.521862 | 14.7045 |  |  |
| 74 | 0.0182476 | 0.399665 | 0.793231 | 0.218568 | 3.62921 |  |  |
| 75 | 0.0014406 | 0.0571251 | 0.352348 | 0.0791012 | 4.4544 |  |  |
| 76 | 0.0710696 | 0.996751 | 0.939081 | 0.406447 | 2.31046 |  |  |
| 77 | 0.0115248 | 0.287987 | 0.676537 | 0.168818 | 4.0075 |  |  |
| 78 | 0.0009604 | 0.0381041 | 0.208249 | 0.0925057 | 2.2512 |  |  |
| 79 | 0.271793 | 3.19577 | 2.67719 | 0.457536 | 5.85133 |  |  |
| 80 | 4.00295 | 20.7787 | 5.45572 | 1.34076 | 4.06912 |  |  |
| 81 | 0.0019208 | 0.0728226 | 0.279906 | 0.0693176 | 4.03802 |  |  |
| 82 | 0.0225694 | 0.447016 | 0.720743 | 0.208785 | 3.45209 |  |  |
| 83 | 0.0033614 | 0.11357 | 0.36401 | 0.138976 | 2.61922 |  |  |
| 84 | 0.107565 | 1.26978 | 1.13833 | 0.463167 | 2.45771 |  |  |
| 85 | 0.0043218 | 0.115114 | 0.309435 | 0.159034 | 1.94571 |  |  |
| 86 | 0.675641 | 7.96587 | 3.09993 | 0.822028 | 3.77107 |  |  |
| 87 | 0.0230496 | 0.438936 | 0.68339 | 0.275469 | 2.48083 |  |  |
| 88 | 0.158946 | 1.68489 | 1.23275 | 0.552479 | 2.2313 |  |  |
| 89 | 0.0489804 | 0.933201 | 1.2999 | 0.312222 | 4.16338 |  |  |
| 90 | 0.0004802 | 0.019606 | 0.0595339 | 0.0595339 | 1 |  |  |
| 91 | 0.0009604 | 0.038627 | 0.275415 | 0.148208 | 1.8583 |  |  |
| 92 | 0.0014406 | 0.0571251 | 0.356215 | 0.0693176 | 5.13888 |  |  |
| 93 | 0.0014406 | 0.0571251 | 0.356215 | 0.0693176 | 5.13888 |  |  |
| 94 | 0.0004802 | 0.019606 | 0.0595339 | 0.0595339 | 1 |  |  |
| 95 | 0.0086436 | 0.319238 | 1.07046 | 0.0693178 | 15.4428 |  |  |
| 96 | 0.007203 | 0.170449 | 0.374719 | 0.168818 | 2.21966 |  |  |
| 97 | 0.0105644 | 0.377809 | 0.695063 | 0.108452 | 6.40893 |  |  |
| 98 | 0.0230496 | 0.429277 | 0.699966 | 0.316229 | 2.21348 |  |  |
| 99 | 0.0004802 | 0.019606 | 0.0595339 | 0.0595339 | 1 |  |  |
| 100 | 0.0422576 | 0.650943 | 0.844078 | 0.331981 | 2.54255 |  |  |
| 101 | 0.021609 | 0.440883 | 0.76877 | 0.255554 | 3.00825 |  |  |
| 102 | 0.0062426 | 0.161838 | 0.363887 | 0.238787 | 1.5239 |  |  |
| 103 | 0.0004802 | 0.019606 | 0.0595339 | 0.0595339 | 1 |  |  |
| 104 | 0.004802 | 0.135104 | 0.360254 | 0.119068 | 3.02562 |  |  |
| 105 | 0.0067228 | 0.183214 | 0.426044 | 0.183607 | 2.32041 |  |  |
| 106 | 0.0009604 | 0.032799 | 0.109284 | 0.143766 | 0.760151 |  |  |
| 107 | 0.0043218 | 0.133759 | 0.415142 | 0.194284 | 2.13678 |  |  |
| 108 | 0.004802 | 0.175808 | 0.416549 | 0.0791013 | 5.26602 |  |  |
| 109 | 0.0028812 | 0.108649 | 0.401192 | 0.0888849 | 4.51362 |  |  |
| 110 | 0.004802 | 0.144343 | 0.429407 | 0.119068 | 3.60641 |  |  |
| 111 | 0.0172872 | 0.429872 | 0.913528 | 0.138635 | 6.58945 |  |  |
| 112 | 0.0441784 | 0.827266 | 1.46452 | 0.258535 | 5.6647 |  |  |
| 113 | 0.0129654 | 0.305999 | 0.521529 | 0.138635 | 3.76188 |  |  |
| 114 | 0.0043218 | 0.130166 | 0.414598 | 0.119068 | 3.48203 |  |  |
| 115 | 0.0028812 | 0.0879606 | 0.318127 | 0.134435 | 2.36641 |  |  |
| 116 | 0.012005 | 0.304908 | 0.662405 | 0.158203 | 4.18707 |  |  |
| 117 | 0.0033614 | 0.125454 | 0.416549 | 0.0693177 | 6.00928 |  |  |
| 118 | 0.033614 | 0.576095 | 0.741242 | 0.274818 | 2.69721 |  |  |
| 119 | 1.2442 | 13.0977 | 3.25586 | 1.26166 | 2.58062 |  |  |
| 120 | 0.0004802 | 0.019606 | 0.0595339 | 0.0595339 | 1 |  |  |
| 121 | 0.0009604 | 0.0381041 | 0.213139 | 0.059534 | 3.58012 |  |  |
| 122 | 1.2562 | 10.1136 | 3.14104 | 1.13944 | 2.75666 |  |  |
| 123 | 0.0076832 | 0.279964 | 0.715213 | 0.0791013 | 9.04174 |  |  |
| 124 | 0.0004802 | 0.019606 | 0.0595339 | 0.0595339 | 1 |  |  |
| 125 | 0.0307328 | 0.613206 | 0.978548 | 0.334751 | 2.92321 |  |  |
| 126 | 0.0062426 | 0.166052 | 0.415143 | 0.128852 | 3.22187 |  |  |
| 127 | 0.0009604 | 0.032799 | 0.109284 | 0.143766 | 0.760153 |  |  |
| 128 | 0.0091238 | 0.213762 | 0.429407 | 0.168818 | 2.54361 |  |  |
| 129 | 0.0038416 | 0.113203 | 0.36401 | 0.119068 | 3.05717 |  |  |
| 130 | 0.0043218 | 0.161866 | 0.567351 | 0.0791013 | 7.17246 |  |  |
| 131 | 0.0139258 | 0.301372 | 0.567359 | 0.178602 | 3.17667 |  |  |
| 132 | 0.0211288 | 0.43227 | 0.653296 | 0.208785 | 3.12904 |  |  |
| 133 | 0.0158466 | 0.405607 | 0.965628 | 0.259604 | 3.71961 |  |  |
| 134 | 0.0004802 | 0.019606 | 0.0595339 | 0.0595339 | 1 |  |  |
| 135 | 0.0331338 | 0.574475 | 0.817037 | 0.278102 | 2.9379 |  |  |
| 136 | 0.142619 | 1.82406 | 1.66803 | 0.406953 | 4.09883 |  |  |
| 137 | 0.0057624 | 0.167719 | 0.480114 | 0.159034 | 3.01893 |  |  |
| 138 | 0.0172872 | 0.392021 | 0.572703 | 0.148419 | 3.8587 |  |  |
| 139 | 0.0004802 | 0.019606 | 0.0595339 | 0.0595339 | 1 |  |  |
| 140 | 0.103243 | 1.71867 | 1.4486 | 0.548732 | 2.63991 |  |  |
| 141 | 0.109005 | 1.639 | 1.30708 | 0.491244 | 2.66076 |  |  |
| 142 | 0.0105644 | 0.242695 | 0.498912 | 0.168818 | 2.95533 |  |  |
| 143 | 0.0081634 | 0.213004 | 0.494509 | 0.138635 | 3.56698 |  |  |
| 144 | 0.0009604 | 0.0381041 | 0.213137 | 0.0595339 | 3.58009 |  |  |
| 145 | 0.0009604 | 0.0381041 | 0.213139 | 0.059534 | 3.58012 |  |  |
| 146 | 0.0052822 | 0.151858 | 0.426326 | 0.159034 | 2.68072 |  |  |
| 147 | 0.004802 | 0.128691 | 0.366904 | 0.119068 | 3.08147 |  |  |
| 148 | 0.0701092 | 1.23909 | 1.57337 | 0.238136 | 6.60702 |  |  |
| 149 | 0.382719 | 3.01274 | 1.74173 | 0.679665 | 2.56263 |  |  |
| 150 | 0.510933 | 4.15388 | 2.11457 | 0.656537 | 3.2208 |  |  |
| 151 | 0.0302526 | 0.554136 | 0.707171 | 0.198169 | 3.56852 |  |  |
| 152 | 0.0076832 | 0.210176 | 0.452414 | 0.189882 | 2.38261 |  |  |
| 153 | 0.0201684 | 0.412621 | 0.607061 | 0.188385 | 3.22244 |  |  |
| 154 | 0.0043218 | 0.12772 | 0.352352 | 0.205167 | 1.71739 |  |  |
| 155 | 0.12101 | 1.95867 | 1.86298 | 0.406122 | 4.58726 |  |  |
| 156 | 0.300605 | 4.24133 | 3.48425 | 0.32702 | 10.6546 |  |  |
| 157 | 0.0225694 | 0.441728 | 0.710671 | 0.238135 | 2.98432 |  |  |
| 158 | 0.079233 | 0.976403 | 0.900186 | 0.371769 | 2.42136 |  |  |
| 159 | 0.0014406 | 0.0560173 | 0.275415 | 0.0693176 | 3.97324 |  |  |
| 160 | 0.0331338 | 0.55617 | 0.868249 | 0.278102 | 3.12205 |  |  |
| 161 | 0.0043218 | 0.119642 | 0.36401 | 0.119068 | 3.05717 |  |  |
| 162 | 0.0014406 | 0.0497959 | 0.228973 | 0.130776 | 1.75089 |  |  |
| 163 | 0.0220892 | 0.593446 | 0.854812 | 0.138635 | 6.16591 |  |  |
| 164 | 0.0014406 | 0.0560173 | 0.275415 | 0.0693176 | 3.97324 |  |  |
| 165 | 0.0067228 | 0.246415 | 0.702319 | 0.0693176 | 10.1319 |  |  |
| 166 | 0.0124852 | 0.29551 | 0.634946 | 0.159035 | 3.9925 |  |  |
| 167 | 0.0038416 | 0.141675 | 0.411103 | 0.0791014 | 5.19716 |  |  |
| 168 | 0.0043218 | 0.166421 | 0.797577 | 0.0693177 | 11.5061 |  |  |
| 169 | 0.0067228 | 0.17771 | 0.498648 | 0.128851 | 3.86994 |  |  |
| 170 | 0.0004802 | 0.019606 | 0.0595339 | 0.0595339 | 1 |  |  |
| 171 | 0.002401 | 0.0758411 | 0.293115 | 0.109284 | 2.68214 |  |  |
| 172 | 0.0019208 | 0.0603798 | 0.219568 | 0.127085 | 1.72772 |  |  |
| 173 | 0.007203 | 0.175675 | 0.397691 | 0.214032 | 1.85809 |  |  |
| 174 | 0.0758716 | 1.19047 | 1.66463 | 0.278102 | 5.98567 |  |  |
| 175 | 0.155585 | 2.43773 | 3.09477 | 0.466488 | 6.6342 |  |  |
| 176 | 0.211288 | 2.27327 | 1.43523 | 0.398002 | 3.6061 |  |  |
| 177 | 0.0340942 | 0.62861 | 0.937455 | 0.333798 | 2.80845 |  |  |
| 178 | 371.622 | 590.912 | 22.1621 | 6.4844 | 3.41775 |  |  |
| 179 | 0.0067228 | 0.178443 | 0.495706 | 0.128851 | 3.84711 |  |  |
| 180 | 0.0067228 | 0.178668 | 0.445903 | 0.159034 | 2.80382 |  |  |
| 181 | 0.0542626 | 0.932691 | 1.31903 | 0.247919 | 5.3204 |  |  |
| 182 | 0.0052822 | 0.156368 | 0.461462 | 0.149251 | 3.09185 |  |  |
| 183 | 0.0076832 | 0.207943 | 0.435763 | 0.119068 | 3.65978 |  |  |
| 184 | 0.227615 | 2.96779 | 2.1651 | 0.358035 | 6.04717 |  |  |
| 185 | 0.0134456 | 0.310902 | 0.559812 | 0.148419 | 3.77184 |  |  |
| 186 | 0.0038416 | 0.141675 | 0.416492 | 0.0791012 | 5.2653 |  |  |
| 187 | 0.0004802 | 0.019606 | 0.0595339 | 0.0595339 | 1 |  |  |
| 188 | 0.0350546 | 0.5311 | 0.596184 | 0.308285 | 1.93387 |  |  |
| 189 | 0.0110446 | 0.272491 | 0.633458 | 0.252441 | 2.50933 |  |  |
| 190 | 1.55009 | 13.8339 | 6.08011 | 1.02269 | 5.94519 |  |  |
| 191 | 0.0182476 | 0.379074 | 0.642437 | 0.199001 | 3.22831 |  |  |
| 192 | 0.353907 | 3.60925 | 1.80638 | 0.577435 | 3.12829 |  |  |
| 193 | 0.169991 | 1.77886 | 1.35307 | 0.39717 | 3.40678 |  |  |
| 194 | 0.0187278 | 0.364798 | 0.580781 | 0.278458 | 2.0857 |  |  |
| 195 | 0.028812 | 0.475888 | 0.580782 | 0.228352 | 2.54336 |  |  |
| 196 | 0.0028812 | 0.0901946 | 0.288523 | 0.137896 | 2.09231 |  |  |
| 197 | 0.0052822 | 0.154709 | 0.426278 | 0.184363 | 2.31216 |  |  |
| 198 | 0.0110446 | 0.26471 | 0.653294 | 0.168818 | 3.86981 |  |  |
| 199 | 0.0014406 | 0.0560173 | 0.275414 | 0.0693176 | 3.97323 |  |  |
| 200 | 0.004802 | 0.155251 | 0.491921 | 0.119068 | 4.13144 |  |  |
| 201 | 0.0043218 | 0.119642 | 0.359161 | 0.119068 | 3.01644 |  |  |
| 202 | 0.0004802 | 0.019606 | 0.0595339 | 0.0595339 | 1 |  |  |
| 203 | 0.705414 | 6.32718 | 2.83333 | 0.613242 | 4.62024 |  |  |
| 204 | 0.0667478 | 1.12534 | 1.34384 | 0.327769 | 4.09995 |  |  |
| 205 | 0.164228 | 2.2694 | 1.89382 | 0.297669 | 6.36216 |  |  |
| 206 | 0.16807 | 2.35002 | 2.27895 | 0.436305 | 5.2233 |  |  |
| 207 | 0.0201684 | 0.423608 | 0.702758 | 0.199001 | 3.53143 |  |  |
| 208 | 0.0110446 | 0.261716 | 0.572703 | 0.168818 | 3.39242 |  |  |
| 209 | 0.0028812 | 0.0984465 | 0.30472 | 0.119068 | 2.55921 |  |  |
| 210 | 0.0033614 | 0.105656 | 0.356028 | 0.119068 | 2.99013 |  |  |
| 211 | 0.002401 | 0.0935985 | 0.498911 | 0.059534 | 8.38027 |  |  |
| 212 | 0.0019208 | 0.0756232 | 0.430344 | 0.0693176 | 6.2083 |  |  |
| 213 | 0.0009604 | 0.032799 | 0.109284 | 0.143765 | 0.760158 |  |  |
| 214 | 0.0028812 | 0.0879606 | 0.318127 | 0.134435 | 2.36641 |  |  |
| 215 | 0.014406 | 0.327288 | 0.57978 | 0.178602 | 3.24622 |  |  |
| 216 | 0.004802 | 0.132478 | 0.356027 | 0.159034 | 2.23868 |  |  |
| 217 | 0.0845152 | 1.23826 | 1.12044 | 0.247919 | 4.5194 |  |  |
| 218 | 0.0196882 | 0.352728 | 0.531268 | 0.295824 | 1.79589 |  |  |
| 219 | 0.009604 | 0.235584 | 0.521537 | 0.119068 | 4.38016 |  |  |
| 220 | 0.0028812 | 0.108649 | 0.401192 | 0.0888849 | 4.51361 |  |  |
| 221 | 0.0086436 | 0.237609 | 0.633458 | 0.227197 | 2.78814 |  |  |
| 222 | 0.152223 | 2.18557 | 2.35722 | 0.268319 | 8.78514 |  |  |
| 223 | 0.0139258 | 0.334129 | 0.692724 | 0.168818 | 4.10338 |  |  |
| 224 | 0.0158466 | 0.336527 | 0.583341 | 0.198169 | 2.94365 |  |  |
| 225 | 0.0019208 | 0.0603798 | 0.219567 | 0.127087 | 1.72769 |  |  |
| 226 | 0.007203 | 0.19639 | 0.498648 | 0.128852 | 3.86994 |  |  |
| 227 | 0.0004802 | 0.019606 | 0.0595339 | 0.0595339 | 1 |  |  |
| 228 | 0.0585844 | 0.846434 | 0.844004 | 0.367819 | 2.29462 |  |  |
| 229 | 0.004802 | 0.164874 | 0.480118 | 0.119068 | 4.03231 |  |  |
| 230 | 0.0067228 | 0.17771 | 0.498648 | 0.128851 | 3.86994 |  |  |
| 231 | 0.0009604 | 0.0381041 | 0.20825 | 0.0925057 | 2.25121 |  |  |
| 232 | 0.0014406 | 0.0497959 | 0.233949 | 0.135086 | 1.73185 |  |  |
| 233 | 0.0163268 | 0.577531 | 0.797576 | 0.108452 | 7.35416 |  |  |
| 234 | 0.0004802 | 0.019606 | 0.0595339 | 0.0595339 | 1 |  |  |
| 235 | 0.0163268 | 0.578701 | 0.869991 | 0.108452 | 8.02187 |  |  |
| 236 | 0.677082 | 5.82104 | 2.78727 | 0.853873 | 3.26426 |  |  |
| 237 | 0.0307328 | 0.705496 | 0.960517 | 0.347461 | 2.76439 |  |  |
| 238 | 3.0776 | 25.1936 | 5.68516 | 1.26249 | 4.50313 |  |  |
| 239 | 0.0153664 | 0.327331 | 0.587059 | 0.199001 | 2.95003 |  |  |
| 240 | 0.002401 | 0.0773766 | 0.321591 | 0.149895 | 2.14544 |  |  |
| 241 | 0.0163268 | 0.338744 | 0.531266 | 0.208785 | 2.54457 |  |  |
| 242 | 1.59042 | 15.2441 | 6.74948 | 0.853041 | 7.91226 |  |  |
| 243 | 0.130614 | 2.38065 | 2.75931 | 0.278103 | 9.92192 |  |  |
| 244 | 0.0091238 | 0.237339 | 0.640984 | 0.228672 | 2.80308 |  |  |
| 245 | 0.101802 | 1.79694 | 2.12215 | 0.40529 | 5.23613 |  |  |
| 246 | 0.626661 | 5.67708 | 3.34215 | 0.61657 | 5.42055 |  |  |
| 247 | 0.138298 | 1.82717 | 1.47197 | 0.388218 | 3.79162 |  |  |
| 248 | 0.729904 | 7.13073 | 3.36307 | 0.774772 | 4.34072 |  |  |
| 249 | 107.799 | 202.577 | 10.7616 | 4.39093 | 2.45088 |  |  |
| 250 | 0.036015 | 0.666062 | 0.951071 | 0.308579 | 3.0821 |  |  |
| 251 | 0.411051 | 4.29009 | 2.55235 | 0.595339 | 4.28722 |  |  |
| 252 | 0.0739508 | 1.09681 | 1.10367 | 0.389259 | 2.83531 |  |  |
| 253 | 0.0028812 | 0.0879606 | 0.318127 | 0.134435 | 2.36641 |  |  |
| 254 | 0.352947 | 3.44202 | 1.95398 | 0.547252 | 3.57052 |  |  |
| 255 | 0.0225694 | 0.458079 | 0.757562 | 0.207953 | 3.64295 |  |  |
| 256 | 0.0629062 | 0.933712 | 0.860235 | 0.391919 | 2.19493 |  |  |
| 257 | 0.0134456 | 0.286026 | 0.561586 | 0.178602 | 3.14435 |  |  |
| 258 | 0.0038416 | 0.115846 | 0.364576 | 0.205168 | 1.77697 |  |  |
| 259 | 0.0129654 | 0.26491 | 0.436008 | 0.218568 | 1.99484 |  |  |
| 260 | 0.0340942 | 0.571194 | 0.76822 | 0.288718 | 2.6608 |  |  |
| 261 | 0.0067228 | 0.198641 | 0.577047 | 0.238787 | 2.41657 |  |  |
| 262 | 0.0009604 | 0.0381041 | 0.208251 | 0.0925057 | 2.25122 |  |  |
| 263 | 0.0643468 | 1.07638 | 1.47433 | 0.38905 | 3.78956 |  |  |
| 264 | 0.0115248 | 0.294524 | 0.680949 | 0.245135 | 2.77785 |  |  |
| 265 | 0.0139258 | 0.303222 | 0.576926 | 0.227914 | 2.53133 |  |  |
| 266 | 1.40843 | 11.196 | 3.55247 | 1.21723 | 2.91848 |  |  |
| 267 | 0.519576 | 6.03114 | 3.73135 | 0.467319 | 7.9846 |  |  |
| 268 | 0.0004802 | 0.019606 | 0.0595339 | 0.0595339 | 1 |  |  |
| 269 | 0.0835548 | 1.33942 | 1.21753 | 0.318068 | 3.82789 |  |  |
| 270 | 0.0019208 | 0.0603798 | 0.219566 | 0.127087 | 1.72768 |  |  |
| 271 | 0.0340942 | 0.607429 | 0.8361 | 0.306112 | 2.73135 |  |  |
| 272 | 0.0163268 | 0.32624 | 0.531266 | 0.230076 | 2.30909 |  |  |
| 273 | 0.0009604 | 0.032799 | 0.109284 | 0.143766 | 0.760153 |  |  |
| 274 | 0.0028812 | 0.0901946 | 0.286624 | 0.119068 | 2.40724 |  |  |
| 275 | 0.0038416 | 0.10904 | 0.321587 | 0.203129 | 1.58317 |  |  |
| 276 | 0.0494606 | 0.965042 | 1.20168 | 0.306621 | 3.9191 |  |  |
| 277 | 0.0729904 | 0.982306 | 0.879951 | 0.29767 | 2.95613 |  |  |
| 278 | 0.0100842 | 0.263576 | 0.628519 | 0.159034 | 3.9521 |  |  |
| 279 | 0.002401 | 0.0758411 | 0.286617 | 0.136814 | 2.09493 |  |  |
| 280 | 0.002401 | 0.0758411 | 0.293123 | 0.119068 | 2.46182 |  |  |
| 281 | 0.0033614 | 0.0977417 | 0.288523 | 0.119068 | 2.42318 |  |  |
| 282 | 0.0196882 | 0.401304 | 0.657521 | 0.188385 | 3.4903 |  |  |
| 283 | 0.0057624 | 0.166218 | 0.416549 | 0.202331 | 2.05875 |  |  |
| 284 | 0.0019208 | 0.0603798 | 0.219566 | 0.127085 | 1.72771 |  |  |
| 285 | 0.21609 | 3.50281 | 1.99708 | 0.34742 | 5.74831 |  |  |
| 286 | 0.004802 | 0.140784 | 0.435767 | 0.128852 | 3.38193 |  |  |
| 287 | 0.0004802 | 0.019606 | 0.0595339 | 0.0595339 | 1 |  |  |
| 288 | 0.002401 | 0.0820625 | 0.286624 | 0.137894 | 2.07858 |  |  |
| 289 | 0.0220892 | 0.482735 | 0.962867 | 0.198169 | 4.85882 |  |  |
| 290 | 0.0100842 | 0.257311 | 0.619391 | 0.168818 | 3.66899 |  |  |
| 291 | 0.0230496 | 0.49675 | 0.71243 | 0.138635 | 5.13888 |  |  |
| 292 | 0.0729904 | 1.75424 | 2.06527 | 0.236472 | 8.73368 |  |  |
| 293 | 0.0105644 | 0.260094 | 0.603901 | 0.119068 | 5.0719 |  |  |
| 294 | 0.0067228 | 0.185999 | 0.574078 | 0.128852 | 4.45534 |  |  |
| 295 | 0.0062426 | 0.226747 | 0.572703 | 0.0791013 | 7.24012 |  |  |
| 296 | 0.639146 | 7.11559 | 2.40008 | 1.16126 | 2.06679 |  |  |
| 297 | 0.0230496 | 0.4568 | 0.765066 | 0.199001 | 3.84454 |  |  |
| 298 | 0.0667478 | 1.16409 | 1.23058 | 0.421595 | 2.91886 |  |  |
| 299 | 0.211768 | 2.43512 | 1.46779 | 0.462692 | 3.17228 |  |  |
| 300 | 0.0283318 | 0.568257 | 0.892835 | 0.208785 | 4.27635 |  |  |
| 301 | 0.0364952 | 0.692485 | 1.03197 | 0.307518 | 3.35581 |  |  |
| 302 | 0.0057624 | 0.158078 | 0.428906 | 0.119068 | 3.6022 |  |  |
| 303 | 0.0633864 | 0.980239 | 0.998124 | 0.400581 | 2.49169 |  |  |
| 304 | 0.0115248 | 0.283398 | 0.59417 | 0.168818 | 3.51959 |  |  |
| 305 | 0.0249704 | 0.426735 | 0.629301 | 0.268318 | 2.34535 |  |  |
| 306 | 0.0067228 | 0.167509 | 0.359161 | 0.168818 | 2.1275 |  |  |
| 307 | 0.0019208 | 0.0662077 | 0.279898 | 0.161793 | 1.72997 |  |  |
| 308 | 0.0921984 | 1.1134 | 0.960738 | 0.555947 | 1.72811 |  |  |
| 309 | 0.002401 | 0.0758411 | 0.288523 | 0.137894 | 2.09235 |  |  |
| 310 | 0.0009604 | 0.0381041 | 0.208251 | 0.0925057 | 2.25122 |  |  |
| 311 | 0.104684 | 1.26082 | 1.04695 | 0.318069 | 3.29157 |  |  |
| 312 | 0.016807 | 0.325159 | 0.499981 | 0.218568 | 2.28753 |  |  |
| 313 | 0.0321734 | 0.506842 | 0.595947 | 0.268318 | 2.22105 |  |  |
| 314 | 0.138298 | 1.89692 | 1.95356 | 0.29767 | 6.56286 |  |  |
| 315 | 0.0009604 | 0.0381041 | 0.208251 | 0.0925057 | 2.25122 |  |  |
| 316 | 0.161347 | 1.97497 | 1.5702 | 0.29767 | 5.27496 |  |  |
| 317 | 0.0182476 | 0.374351 | 0.594168 | 0.199001 | 2.98576 |  |  |
| 318 | 0.0057624 | 0.159411 | 0.414596 | 0.216977 | 1.91079 |  |  |
| 319 | 0.0139258 | 0.319033 | 0.549938 | 0.168818 | 3.25758 |  |  |
| 320 | 0.0081634 | 0.233394 | 0.59417 | 0.159034 | 3.73611 |  |  |
| 321 | 0.0297724 | 0.627991 | 1.08019 | 0.178602 | 6.04803 |  |  |
| 322 | 0.0158466 | 0.356002 | 0.618256 | 0.246648 | 2.50664 |  |  |
| 323 | 0.0153664 | 0.328857 | 0.643923 | 0.178602 | 3.60535 |  |  |
| 324 | 0.0153664 | 0.342994 | 0.657542 | 0.178602 | 3.68161 |  |  |
| 325 | 0.0110446 | 0.289438 | 0.629301 | 0.245135 | 2.56716 |  |  |
| 326 | 0.0081634 | 0.199681 | 0.426322 | 0.168818 | 2.52533 |  |  |
| 327 | 0.0019208 | 0.0728226 | 0.279906 | 0.0693176 | 4.03802 |  |  |
| 328 | 0.0019208 | 0.0662077 | 0.275415 | 0.143737 | 1.9161 |  |  |
| 329 | 0.0110446 | 0.252232 | 0.445481 | 0.178602 | 2.49427 |  |  |
| 330 | 0.0028812 | 0.0848895 | 0.293119 | 0.119068 | 2.46178 |  |  |
| 331 | 0.002401 | 0.0852566 | 0.36025 | 0.148144 | 2.43176 |  |  |
| 332 | 0.0052822 | 0.195414 | 0.594169 | 0.0791013 | 7.5115 |  |  |
| 333 | 0.0513814 | 0.901324 | 1.14966 | 0.238136 | 4.82775 |  |  |
| 334 | 0.045619 | 0.936405 | 1.00027 | 0.217737 | 4.59395 |  |  |
| 335 | 0.0393764 | 0.801617 | 1.37936 | 0.247087 | 5.58249 |  |  |
| 336 | 0.0100842 | 0.269222 | 0.713466 | 0.159034 | 4.48624 |  |  |
| 337 | 0.272754 | 3.97627 | 2.66577 | 0.287054 | 9.28665 |  |  |
| 338 | 0.0153664 | 0.369135 | 0.826782 | 0.128851 | 6.41655 |  |  |
| 339 | 0.019208 | 0.450595 | 0.889673 | 0.138635 | 6.41737 |  |  |
| 340 | 0.736147 | 5.95139 | 2.88532 | 0.526021 | 5.48518 |  |  |
| 341 | 0.0019208 | 0.0728226 | 0.279898 | 0.0693176 | 4.0379 |  |  |
| 342 | 0.0019208 | 0.0667585 | 0.293115 | 0.161794 | 1.81166 |  |  |
| 343 | 0.654993 | 5.37338 | 2.61136 | 0.835138 | 3.12686 |  |  |
| 344 | 0.0931588 | 1.29157 | 1.50306 | 0.358035 | 4.19807 |  |  |
| 345 | 0.062426 | 0.829262 | 0.804256 | 0.358035 | 2.2463 |  |  |
| 346 | 0.0158466 | 0.305298 | 0.511166 | 0.293175 | 1.74355 |  |  |
| 347 | 0.157025 | 1.76872 | 1.10885 | 0.707457 | 1.56737 |  |  |
| 348 | 0.004802 | 0.13698 | 0.429407 | 0.119068 | 3.6064 |  |  |
| 349 | 0.0518616 | 0.720675 | 0.79282 | 0.327852 | 2.41822 |  |  |
| 350 | 0.235298 | 2.31846 | 1.55269 | 0.680793 | 2.2807 |  |  |
| 351 | 0.0989212 | 1.78327 | 1.63463 | 0.308285 | 5.30233 |  |  |
| 352 | 0.009604 | 0.345368 | 0.703953 | 0.0986686 | 7.13451 |  |  |
| 353 | 0.113807 | 1.52351 | 1.4999 | 0.29767 | 5.03879 |  |  |
| 354 | 0.199283 | 1.86433 | 1.24015 | 0.588362 | 2.10781 |  |  |
| 355 | 0.021609 | 0.441998 | 0.670886 | 0.291942 | 2.29801 |  |  |
| 356 | 0.0038416 | 0.11377 | 0.365525 | 0.203127 | 1.79949 |  |  |
| 357 | 0.0331338 | 0.638957 | 0.905729 | 0.297178 | 3.04776 |  |  |
| 358 | 0.0100842 | 0.295598 | 0.664106 | 0.158202 | 4.19783 |  |  |
| 359 | 0.0004802 | 0.019606 | 0.0595339 | 0.0595339 | 1 |  |  |
| 360 | 0.0509012 | 0.724214 | 0.822297 | 0.414243 | 1.98506 |  |  |
| 361 | 1.4478 | 12.138 | 2.80995 | 1.85672 | 1.51339 |  |  |
| 362 | 0.0340942 | 0.594464 | 0.772906 | 0.319125 | 2.42196 |  |  |
| 363 | 0.0009604 | 0.032799 | 0.109284 | 0.143766 | 0.760151 |  |  |
| 364 | 0.0129654 | 0.273731 | 0.518373 | 0.178602 | 2.9024 |  |  |
| 365 | 0.002401 | 0.0820625 | 0.28506 | 0.153509 | 1.85697 |  |  |
| 366 | 0.002401 | 0.0758411 | 0.288523 | 0.137894 | 2.09235 |  |  |
| 367 | 0.0393764 | 0.693944 | 0.963855 | 0.340651 | 2.82945 |  |  |
| 368 | 0.0753914 | 1.13647 | 1.28676 | 0.337636 | 3.81108 |  |  |
| 369 | 0.0302526 | 0.687209 | 1.15519 | 0.284001 | 4.06756 |  |  |
| 370 | 0.038416 | 0.66515 | 0.861352 | 0.311409 | 2.76598 |  |  |
| 371 | 0.002401 | 0.0758411 | 0.286624 | 0.136816 | 2.09496 |  |  |
| 372 | 0.124372 | 1.89858 | 1.6132 | 0.306621 | 5.26123 |  |  |
| 373 | 0.0302526 | 0.644514 | 1.12757 | 0.356597 | 3.16204 |  |  |
| 374 | 0.0009604 | 0.032799 | 0.109284 | 0.143766 | 0.760151 |  |  |
| 375 | 0.0043218 | 0.138873 | 0.416545 | 0.109284 | 3.81157 |  |  |
| 376 | 0.0561834 | 0.98214 | 0.978548 | 0.37879 | 2.58335 |  |  |
| 377 | 0.0014406 | 0.0566022 | 0.308397 | 0.0595339 | 5.18019 |  |  |
| 378 | 0.0091238 | 0.237975 | 0.617399 | 0.210321 | 2.9355 |  |  |
| 379 | 0.0009604 | 0.032799 | 0.109284 | 0.143766 | 0.760154 |  |  |
| 380 | 0.538304 | 4.54139 | 2.87121 | 0.676104 | 4.24669 |  |  |
| 381 | 4.15037 | 31.3255 | 6.3453 | 1.76811 | 3.58874 |  |  |
| 382 | 1.4334 | 8.72431 | 3.09973 | 0.713575 | 4.34395 |  |  |
| 383 | 0.161347 | 1.96354 | 1.49309 | 0.487719 | 3.06137 |  |  |
| 384 | 0.427858 | 3.9219 | 2.49655 | 0.54642 | 4.56893 |  |  |
| 385 | 0.0686686 | 0.912046 | 1.00027 | 0.444997 | 2.24781 |  |  |
| 386 | 0.0398566 | 0.601827 | 0.802274 | 0.409956 | 1.95697 |  |  |
| 387 | 0.650671 | 6.38577 | 2.06508 | 1.42668 | 1.44747 |  |  |
| 388 | 0.187758 | 2.3573 | 2.20597 | 0.677037 | 3.25828 |  |  |
| 389 | 0.0052822 | 0.142449 | 0.359161 | 0.159034 | 2.25838 |  |  |
| 390 | 0.0316932 | 0.541808 | 0.811219 | 0.268318 | 3.02335 |  |  |
| 391 | 0.108045 | 1.64915 | 1.30099 | 0.518194 | 2.51062 |  |  |
| 392 | 0.0374556 | 0.701185 | 1.14866 | 0.358157 | 3.20715 |  |  |
| 393 | 0.12101 | 2.08186 | 2.35899 | 0.42122 | 5.60038 |  |  |
| 394 | 0.138298 | 1.96941 | 1.68222 | 0.467319 | 3.59973 |  |  |
| 395 | 0.0475398 | 0.822523 | 1.01072 | 0.228352 | 4.42613 |  |  |
| 396 | 0.0364952 | 0.611548 | 0.838966 | 0.303639 | 2.76304 |  |  |
| 397 | 0.102283 | 1.31485 | 1.14561 | 0.437136 | 2.62071 |  |  |
| 398 | 0.124372 | 1.46429 | 1.30938 | 0.39717 | 3.29679 |  |  |
| 399 | 0.067228 | 1.27859 | 1.41918 | 0.335972 | 4.22409 |  |  |
| 400 | 0.0019208 | 0.0667585 | 0.293123 | 0.145524 | 2.01426 |  |  |
| 401 | 0.004802 | 0.175808 | 0.491925 | 0.0791013 | 6.21893 |  |  |
| 402 | 0.0235298 | 0.829886 | 1.16555 | 0.147587 | 7.89737 |  |  |
| 403 | 0.0091238 | 0.330255 | 0.779382 | 0.0986682 | 7.89902 |  |  |
| 404 | 0.0283318 | 0.470576 | 0.649507 | 0.317613 | 2.04496 |  |  |
| 405 | 0.150783 | 1.84027 | 1.65174 | 0.318068 | 5.19302 |  |  |
| 406 | 0.004802 | 0.128882 | 0.374716 | 0.159034 | 2.35619 |  |  |
| 407 | 0.0004802 | 0.019606 | 0.0595339 | 0.0595339 | 1 |  |  |
| 408 | 0.0244902 | 0.470522 | 0.629301 | 0.270565 | 2.32588 |  |  |
| 409 | 0.026411 | 0.486613 | 0.71243 | 0.238136 | 2.99169 |  |  |
| 410 | 0.0057624 | 0.146464 | 0.366904 | 0.183281 | 2.00186 |  |  |
| 411 | 0.0009604 | 0.0381041 | 0.213139 | 0.0595342 | 3.58011 |  |  |
| 412 | 0.0038416 | 0.113394 | 0.356215 | 0.183285 | 1.9435 |  |  |
| 413 | 0.002401 | 0.0820625 | 0.286624 | 0.137894 | 2.07858 |  |  |
| 414 | 0.0019208 | 0.0603798 | 0.219568 | 0.127085 | 1.72772 |  |  |
| 415 | 0.0038416 | 0.115812 | 0.411102 | 0.119068 | 3.45267 |  |  |
| 416 | 0.0009604 | 0.0381041 | 0.213139 | 0.0595339 | 3.58013 |  |  |
| 417 | 0.181516 | 3.40491 | 2.27967 | 0.226688 | 10.0564 |  |  |
| 418 | 0.453309 | 4.07741 | 2.96074 | 0.537469 | 5.50867 |  |  |
| 419 | 106.986 | 319.294 | 19.7753 | 3.14448 | 6.28889 |  |  |
| 420 | 3.78446 | 27.6146 | 5.45129 | 2.07806 | 2.62326 |  |  |
| 421 | 0.0974806 | 1.2634 | 1.24756 | 0.377603 | 3.30389 |  |  |
| 422 | 0.508052 | 4.02982 | 2.3059 | 0.972061 | 2.37217 |  |  |
| 423 | 2.58059 | 19.5737 | 5.43145 | 1.29913 | 4.18084 |  |  |
| 424 | 1.19474 | 12.5886 | 5.12042 | 1.13281 | 4.52012 |  |  |
| 425 | 0.227615 | 2.75436 | 2.36586 | 0.541985 | 4.36518 |  |  |
| 426 | 0.385601 | 3.55594 | 2.28954 | 0.798756 | 2.86638 |  |  |
| 427 | 0.0412972 | 0.666862 | 1.01257 | 0.37006 | 2.73623 |  |  |
| 428 | 0.152223 | 1.73357 | 1.42782 | 0.407786 | 3.50141 |  |  |
| 429 | 0.278036 | 3.11528 | 2.73268 | 0.515406 | 5.302 |  |  |
| 430 | 0.0302526 | 0.756244 | 1.65417 | 0.218568 | 7.5682 |  |  |
| 431 | 0.0854756 | 1.36016 | 1.18594 | 0.493804 | 2.40164 |  |  |
| 432 | 0.0177674 | 0.434505 | 0.773942 | 0.138635 | 5.58259 |  |  |
| 433 | 0.566636 | 5.02752 | 2.33388 | 0.987616 | 2.36314 |  |  |
| 434 | 0.28812 | 2.63036 | 1.35453 | 0.577672 | 2.34482 |  |  |
| 435 | 0.0062426 | 0.23358 | 0.890899 | 0.147726 | 6.03074 |  |  |
| 436 | 0.116689 | 1.85043 | 1.69041 | 0.328684 | 5.14297 |  |  |
| 437 | 0.0182476 | 0.34603 | 0.589547 | 0.278102 | 2.11989 |  |  |
| 438 | 0.0009604 | 0.032799 | 0.109284 | 0.143766 | 0.760153 |  |  |
| 439 | 0.0076832 | 0.187779 | 0.415612 | 0.159034 | 2.61335 |  |  |
| 440 | 0.0701092 | 1.05265 | 1.21813 | 0.288717 | 4.2191 |  |  |
| 441 | 0.0163268 | 0.343282 | 0.653503 | 0.208785 | 3.13003 |  |  |
| 442 | 0.0009604 | 0.0381041 | 0.208247 | 0.0925057 | 2.25118 |  |  |
| 443 | 0.014406 | 0.307523 | 0.549192 | 0.208785 | 2.63042 |  |  |
| 444 | 0.0518616 | 0.783439 | 0.996464 | 0.359111 | 2.77481 |  |  |
| 445 | 0.0153664 | 0.307944 | 0.499393 | 0.238136 | 2.09709 |  |  |
| 446 | 0.0019208 | 0.0739304 | 0.308397 | 0.0693176 | 4.44904 |  |  |
| 447 | 0.0052822 | 0.143916 | 0.359161 | 0.159034 | 2.25839 |  |  |
| 448 | 0.0014406 | 0.0497959 | 0.219564 | 0.127083 | 1.72772 |  |  |
| 449 | 0.004802 | 0.135267 | 0.359157 | 0.205168 | 1.75055 |  |  |
| 450 | 0.0038416 | 0.107567 | 0.302241 | 0.203125 | 1.48796 |  |  |
| 451 | 0.0081634 | 0.294429 | 0.628515 | 0.0986687 | 6.36996 |  |  |
| 452 | 0.0009604 | 0.0381041 | 0.208247 | 0.0925077 | 2.25113 |  |  |
| 453 | 7.88344 | 34.9654 | 4.98794 | 1.12782 | 4.42265 |  |  |
| 454 | 0.0110446 | 0.25806 | 0.536337 | 0.188385 | 2.84702 |  |  |
| 455 | 0.0028812 | 0.107541 | 0.356214 | 0.0693176 | 5.13887 |  |  |
| 456 | 0.139258 | 1.96451 | 1.52368 | 0.380177 | 4.00782 |  |  |
| 457 | 0.0004802 | 0.019606 | 0.0595339 | 0.0595339 | 1 |  |  |
| 458 | 8.0083 | 24.6972 | 3.41035 | 1.72815 | 1.97342 |  |  |
| 459 | 0.0518616 | 0.749613 | 0.896814 | 0.376726 | 2.38055 |  |  |
| 460 | 0.0062426 | 0.227855 | 0.511803 | 0.088885 | 5.75804 |  |  |
| 461 | 10.393 | 59.535 | 10.8225 | 1.64988 | 6.55956 |  |  |
| 462 | 0.0686686 | 0.966209 | 1.07703 | 0.367265 | 2.93256 |  |  |
| 463 | 0.0182476 | 0.363043 | 0.639968 | 0.255554 | 2.50424 |  |  |
| 464 | 0.0091238 | 0.218674 | 0.445903 | 0.178601 | 2.49664 |  |  |
| 465 | 0.0201684 | 0.419289 | 0.727489 | 0.208785 | 3.48439 |  |  |
| 466 | 0.0451388 | 0.758494 | 1.02361 | 0.228352 | 4.4826 |  |  |
| 467 | 0.038416 | 0.659416 | 0.845653 | 0.278102 | 3.0408 |  |  |
| 468 | 0.0259308 | 0.575811 | 1.10281 | 0.268318 | 4.11009 |  |  |
| 469 | 0.0489804 | 0.89404 | 1.3343 | 0.362167 | 3.68422 |  |  |
| 470 | 0.012005 | 0.267134 | 0.51297 | 0.188385 | 2.72298 |  |  |
| 471 | 0.117649 | 1.74524 | 1.88117 | 0.398002 | 4.72655 |  |  |
| 472 | 0.0004802 | 0.019606 | 0.0595339 | 0.0595339 | 1 |  |  |
| 473 | 0.0134456 | 0.311973 | 0.687901 | 0.28412 | 2.42116 |  |  |
| 474 | 0.0470596 | 0.907364 | 0.954383 | 0.371316 | 2.57027 |  |  |
| 475 | 0.0667478 | 1.5003 | 1.39491 | 0.167986 | 8.30373 |  |  |
| 476 | 0.009604 | 0.225166 | 0.503542 | 0.240606 | 2.09281 |  |  |
| 477 | 0.045619 | 0.751616 | 1.04771 | 0.275791 | 3.79894 |  |  |
| 478 | 0.0427378 | 0.702153 | 0.820362 | 0.316857 | 2.58906 |  |  |
| 479 | 0.0629062 | 0.896045 | 0.93381 | 0.451887 | 2.06647 |  |  |
| 480 | 0.322694 | 2.94107 | 2.11219 | 0.466488 | 4.52786 |  |  |
| 481 | 0.275635 | 2.49734 | 1.55204 | 0.685887 | 2.26282 |  |  |
| 482 | 0.007203 | 0.166837 | 0.382363 | 0.231515 | 1.65157 |  |  |
| 483 | 0.040817 | 0.606312 | 0.683364 | 0.435232 | 1.57012 |  |  |
| 484 | 0.004802 | 0.132014 | 0.312681 | 0.168818 | 1.85217 |  |  |
| 485 | 0.021609 | 0.526194 | 0.82677 | 0.218568 | 3.78266 |  |  |
| 486 | 0.028812 | 0.639787 | 1.04741 | 0.207953 | 5.03677 |  |  |
| 487 | 0.0657874 | 0.942037 | 1.05636 | 0.36084 | 2.92751 |  |  |
| 488 | 0.0014406 | 0.0560173 | 0.275414 | 0.0693176 | 3.97323 |  |  |
| 489 | 0.109486 | 1.6482 | 1.77759 | 0.467012 | 3.8063 |  |  |
| 490 | 0.0158466 | 0.350924 | 0.710671 | 0.178602 | 3.97908 |  |  |
| 491 | 0.0412972 | 0.636303 | 0.818151 | 0.371243 | 2.20382 |  |  |
| 492 | 0.0585844 | 0.784641 | 0.839291 | 0.318069 | 2.63871 |  |  |
| 493 | 0.0038416 | 0.107567 | 0.302237 | 0.203129 | 1.48791 |  |  |
| 494 | 0.118609 | 2.41376 | 1.84572 | 0.207121 | 8.91129 |  |  |
| 495 | 0.0225694 | 0.409853 | 0.595947 | 0.288019 | 2.06913 |  |  |
| 496 | 0.0533022 | 0.840404 | 0.979249 | 0.385914 | 2.53748 |  |  |
| 497 | 0.002401 | 0.0758411 | 0.288519 | 0.109284 | 2.64008 |  |  |
| 498 | 0.0019208 | 0.0739304 | 0.356215 | 0.0693176 | 5.13889 |  |  |
| 499 | 0.0249704 | 0.501646 | 0.935274 | 0.229969 | 4.06696 |  |  |
| 500 | 0.0086436 | 0.19619 | 0.429937 | 0.178602 | 2.40724 |  |  |
| 501 | 0.0153664 | 0.335645 | 0.657539 | 0.178602 | 3.6816 |  |  |
| 502 | 0.0124852 | 0.307934 | 0.663368 | 0.280484 | 2.36508 |  |  |
| 503 | 0.0201684 | 0.410222 | 0.767321 | 0.228352 | 3.36026 |  |  |
| 504 | 0.0033614 | 0.124869 | 0.411103 | 0.0791013 | 5.19717 |  |  |
| 505 | 0.0158466 | 0.346995 | 0.657542 | 0.178602 | 3.68161 |  |  |
| 506 | 0.0364952 | 0.65647 | 0.998124 | 0.218568 | 4.56665 |  |  |
| 507 | 0.0883568 | 1.32983 | 1.59133 | 0.327852 | 4.8538 |  |  |
| 508 | 0.004802 | 0.122661 | 0.312583 | 0.168818 | 1.8516 |  |  |
| 509 | 0.0509012 | 0.733825 | 0.778554 | 0.287885 | 2.70439 |  |  |
| 510 | 0.0019208 | 0.0603798 | 0.219568 | 0.127087 | 1.7277 |  |  |
| 511 | 0.0110446 | 0.281984 | 0.696406 | 0.178601 | 3.89922 |  |  |
| 512 | 0.0091238 | 0.212654 | 0.429403 | 0.168818 | 2.54358 |  |  |
| 513 | 0.007203 | 0.206704 | 0.628107 | 0.148419 | 4.23198 |  |  |
| 514 | 0.0014406 | 0.0560173 | 0.275414 | 0.0693176 | 3.97323 |  |  |
| 515 | 0.0422576 | 1.22619 | 1.60689 | 0.188385 | 8.52981 |  |  |
| 516 | 0.0009604 | 0.032799 | 0.109284 | 0.143766 | 0.760154 |  |  |
| 517 | 0.0379358 | 1.30462 | 1.76305 | 0.138635 | 12.7172 |  |  |
| 518 | 0.242021 | 8.02655 | 3.46307 | 0.31391 | 11.032 |  |  |
| 519 | 0.0081634 | 0.293844 | 0.573573 | 0.0986687 | 5.81313 |  |  |
| 520 | 0.173832 | 2.59636 | 2.13074 | 0.615047 | 3.46436 |  |  |
| 521 | 4.61424 | 41.531 | 10.6897 | 1.32682 | 8.05663 |  |  |
| 522 | 0.0393764 | 0.732377 | 0.879051 | 0.257703 | 3.4111 |  |  |
| 523 | 0.0062426 | 0.167725 | 0.403654 | 0.159034 | 2.53816 |  |  |
| 524 | 0.0801934 | 1.1347 | 1.10618 | 0.267487 | 4.13546 |  |  |
| 525 | 0.0244902 | 0.683118 | 1.03465 | 0.178602 | 5.79308 |  |  |
| 526 | 0.0105644 | 0.289995 | 0.63866 | 0.215266 | 2.96684 |  |  |
| 527 | 0.0081634 | 0.294429 | 0.627591 | 0.0986687 | 6.36059 |  |  |
| 528 | 0.0201684 | 0.720376 | 1.38377 | 0.118236 | 11.7034 |  |  |
| 529 | 0.0033614 | 0.124869 | 0.356028 | 0.0791013 | 4.50092 |  |  |
| 530 | 0.0086436 | 0.319698 | 1.06057 | 0.108453 | 9.77908 |  |  |
| 531 | 0.0542626 | 1.90191 | 2.38847 | 0.225857 | 10.5752 |  |  |
| 532 | 0.0172872 | 0.616743 | 1.19191 | 0.147587 | 8.07598 |  |  |
| 533 | 0.0163268 | 0.585692 | 1.02557 | 0.167986 | 6.10509 |  |  |
| 534 | 0.0187278 | 0.497764 | 0.924718 | 0.207953 | 4.44677 |  |  |
| 535 | 0.0533022 | 1.36096 | 2.04882 | 0.138635 | 14.7785 |  |  |
| 536 | 1.80843 | 13.7843 | 3.535 | 0.843258 | 4.19207 |  |  |
| 537 | 1.32871 | 10.2417 | 4.53663 | 0.994173 | 4.56322 |  |  |
| 538 | 0.009604 | 0.322448 | 0.913528 | 0.159034 | 5.74422 |  |  |
| 539 | 0.012005 | 0.285493 | 0.576926 | 0.188385 | 3.06248 |  |  |
| 540 | 0.0004802 | 0.019606 | 0.0595339 | 0.0595339 | 1 |  |  |
| 541 | 0.0547428 | 1.3348 | 1.83252 | 0.167987 | 10.9087 |  |  |
| 542 | 0.0019208 | 0.0728226 | 0.279906 | 0.0693176 | 4.03802 |  |  |
| 543 | 1.13039 | 11.1786 | 3.56942 | 0.723358 | 4.93451 |  |  |
| 544 | 0.0206486 | 0.502919 | 0.703951 | 0.148419 | 4.74299 |  |  |
| 545 | 0.0009604 | 0.0381041 | 0.208249 | 0.0925057 | 2.2512 |  |  |
| 546 | 0.0019208 | 0.0739304 | 0.355335 | 0.0693171 | 5.12623 |  |  |
| 547 | 0.0004802 | 0.019606 | 0.0595339 | 0.0595339 | 1 |  |  |
| 548 | 0.0014406 | 0.0560173 | 0.279902 | 0.0693176 | 4.03796 |  |  |
| 549 | 0.0014406 | 0.0497959 | 0.228969 | 0.130774 | 1.75088 |  |  |
| 550 | 10.8775 | 50.773 | 11.716 | 1.34306 | 8.72334 |  |  |
| 551 | 0.351026 | 3.69217 | 2.5349 | 0.565988 | 4.47871 |  |  |
| 552 | 0.555111 | 4.76182 | 2.2232 | 0.96409 | 2.30601 |  |  |
| 553 | 0.396645 | 3.84672 | 1.86727 | 0.903624 | 2.06643 |  |  |
| 554 | 0.0542626 | 1.03521 | 1.17545 | 0.357203 | 3.2907 |  |  |
| 555 | 0.201684 | 2.71434 | 1.78431 | 0.536637 | 3.32499 |  |  |
| 556 | 0.0388962 | 0.813464 | 1.20526 | 0.384238 | 3.13674 |  |  |
| 557 | 0.0028812 | 0.109234 | 0.430348 | 0.0693176 | 6.20836 |  |  |
| 558 | 0.342383 | 3.22409 | 1.94067 | 0.477103 | 4.06761 |  |  |
| 559 | 1.29318 | 14.6721 | 5.23874 | 0.620531 | 8.44235 |  |  |
| 560 | 0.0057624 | 0.209419 | 0.489625 | 0.088885 | 5.50853 |  |  |
| 561 | 0.159426 | 1.7785 | 1.3347 | 0.506454 | 2.63538 |  |  |
| 562 | 0.64875 | 7.43365 | 3.05062 | 0.706287 | 4.31923 |  |  |
| 563 | 0.373596 | 4.84903 | 3.89102 | 0.485222 | 8.01904 |  |  |
| 564 | 0.0955598 | 1.21961 | 1.27547 | 0.337636 | 3.77765 |  |  |
| 565 | 0.062426 | 1.03301 | 1.01256 | 0.409891 | 2.47032 |  |  |
| 566 | 0.108045 | 1.52122 | 1.58712 | 0.552354 | 2.87338 |  |  |
| 567 | 0.0691488 | 1.00768 | 1.02909 | 0.437708 | 2.35109 |  |  |
| 568 | 0.0955598 | 1.13881 | 0.934668 | 0.427353 | 2.18711 |  |  |
| 569 | 0.0211288 | 0.451005 | 0.811223 | 0.168818 | 4.80531 |  |  |
| 570 | 0.0283318 | 0.991566 | 1.05832 | 0.147587 | 7.17078 |  |  |
| 571 | 0.0965202 | 3.3386 | 2.59497 | 0.167155 | 15.5244 |  |  |
| 572 | 0.028812 | 0.562122 | 0.892835 | 0.178602 | 4.99903 |  |  |
| 573 | 0.0028812 | 0.0848895 | 0.288523 | 0.119068 | 2.42319 |  |  |
| 574 | 0.0033614 | 0.107129 | 0.320172 | 0.119068 | 2.68899 |  |  |
| 575 | 0.0014406 | 0.0497959 | 0.237959 | 0.119735 | 1.98738 |  |  |
| 576 | 0.0019208 | 0.0662077 | 0.275415 | 0.161203 | 1.7085 |  |  |
| 577 | 0.0004802 | 0.019606 | 0.0595339 | 0.0595339 | 1 |  |  |
| 578 | 0.0019208 | 0.0728226 | 0.279906 | 0.0693176 | 4.03802 |  |  |
| 579 | 0.11909 | 1.65455 | 1.63419 | 0.336804 | 4.85206 |  |  |
| 580 | 0.0014406 | 0.0566022 | 0.304724 | 0.093911 | 3.24481 |  |  |
| 581 | 0.43266 | 7.16883 | 3.88166 | 0.305789 | 12.6939 |  |  |
| 582 | 0.0470596 | 0.819378 | 1.05257 | 0.217737 | 4.83413 |  |  |
| 583 | 0.0076832 | 0.275452 | 0.81047 | 0.119068 | 6.80678 |  |  |
| 584 | 0.0571438 | 0.845668 | 0.997192 | 0.278102 | 3.5857 |  |  |
| 585 | 0.0019208 | 0.0658421 | 0.293123 | 0.161203 | 1.81835 |  |  |
| 586 | 0.0110446 | 0.239667 | 0.491903 | 0.230074 | 2.13802 |  |  |
| 587 | 0.0028812 | 0.0848895 | 0.288523 | 0.119068 | 2.42318 |  |  |
| 588 | 0.0412972 | 0.69583 | 0.810108 | 0.228352 | 3.54763 |  |  |
| 589 | 0.0518616 | 0.854603 | 1.01076 | 0.218568 | 4.62448 |  |  |
| 590 | 0.0100842 | 0.234284 | 0.429407 | 0.178601 | 2.40428 |  |  |
| 591 | 0.16903 | 2.30092 | 1.73998 | 0.296838 | 5.86172 |  |  |
| 592 | 0.0163268 | 0.341337 | 0.573573 | 0.198169 | 2.89436 |  |  |
| 593 | 0.0004802 | 0.019606 | 0.0595339 | 0.0595339 | 1 |  |  |
| 594 | 0.0043218 | 0.119642 | 0.360254 | 0.119068 | 3.02561 |  |  |
| 595 | 0.0124852 | 0.291837 | 0.643925 | 0.178602 | 3.60537 |  |  |
| 596 | 0.007203 | 0.187491 | 0.429405 | 0.128851 | 3.33256 |  |  |
| 597 | 0.014406 | 0.372032 | 0.860688 | 0.138635 | 6.2083 |  |  |
| 598 | 0.0533022 | 1.00895 | 1.29649 | 0.207953 | 6.23453 |  |  |
| 599 | 0.0206486 | 0.437731 | 0.810471 | 0.188385 | 4.30219 |  |  |
| 600 | 0.0043218 | 0.12772 | 0.356028 | 0.210051 | 1.69496 |  |  |
| 601 | 0.0067228 | 0.195064 | 0.491929 | 0.128851 | 3.8178 |  |  |
| 602 | 6.06108 | 32.915 | 5.8748 | 0.878234 | 6.68933 |  |  |
| 603 | 0.0220892 | 0.513505 | 0.727488 | 0.148419 | 4.90158 |  |  |
| 604 | 0.0067228 | 0.172492 | 0.429405 | 0.119068 | 3.60639 |  |  |
| 605 | 1.56161 | 13.057 | 4.28883 | 0.593675 | 7.22421 |  |  |
| 606 | 0.02401 | 0.525555 | 0.771835 | 0.158202 | 4.87879 |  |  |
| 607 | 0.0009604 | 0.032799 | 0.109284 | 0.143766 | 0.760155 |  |  |
| 608 | 0.0979608 | 1.94254 | 1.55879 | 0.505622 | 3.08291 |  |  |
| 609 | 0.0172872 | 0.634965 | 1.89754 | 0.12802 | 14.8222 |  |  |
| 610 | 0.124372 | 1.64052 | 1.61682 | 0.34742 | 4.6538 |  |  |
| 611 | 0.170471 | 1.95609 | 1.63419 | 0.366987 | 4.45299 |  |  |
| 612 | 1.4017 | 14.6251 | 6.83246 | 0.601795 | 11.3535 |  |  |
| 613 | 0.205045 | 2.2111 | 1.81411 | 0.376771 | 4.81488 |  |  |
| 614 | 0.0859558 | 1.27628 | 1.16255 | 0.298502 | 3.89463 |  |  |
| 615 | 0.169991 | 2.34266 | 2.24972 | 0.337636 | 6.66314 |  |  |
| 616 | 0.0921984 | 1.17791 | 1.21687 | 0.34742 | 3.50258 |  |  |
| 617 | 0.0100842 | 0.277379 | 0.727442 | 0.149251 | 4.87396 |  |  |
| 618 | 0.060025 | 1.45422 | 1.30476 | 0.167986 | 7.76709 |  |  |
| 619 | 0.0004802 | 0.019606 | 0.0595339 | 0.0595339 | 1 |  |  |
| 620 | 0.153664 | 3.616 | 3.07433 | 0.344092 | 8.93461 |  |  |
| 621 | 0.0004802 | 0.019606 | 0.0595339 | 0.0595339 | 1 |  |  |
| 622 | 0.0004802 | 0.019606 | 0.0595339 | 0.0595339 | 1 |  |  |
| 623 | 0.343343 | 4.00748 | 2.98305 | 0.375939 | 7.93491 |  |  |
| 624 | 0.238659 | 2.79215 | 1.77721 | 0.306621 | 5.7961 |  |  |
| 625 | 0.0273714 | 0.509488 | 0.657693 | 0.218568 | 3.0091 |  |  |
| 626 | 0.0009604 | 0.0381041 | 0.213139 | 0.0595339 | 3.58013 |  |  |
| 627 | 0.0028812 | 0.107541 | 0.356027 | 0.0791013 | 4.50091 |  |  |
| 628 | 0.0076832 | 0.277101 | 0.567296 | 0.0986687 | 5.7495 |  |  |
| 629 | 0.0177674 | 0.353961 | 0.58706 | 0.228352 | 2.57086 |  |  |
| 630 | 0.0139258 | 0.341894 | 0.617399 | 0.268858 | 2.29637 |  |  |
| 631 | 0.012005 | 0.238132 | 0.400582 | 0.228352 | 1.75423 |  |  |
| 632 | 0.0028812 | 0.0848895 | 0.293119 | 0.119068 | 2.46178 |  |  |
| 633 | 0.004802 | 0.142049 | 0.429933 | 0.189882 | 2.26421 |  |  |
| 634 | 0.36063 | 5.21745 | 3.06269 | 0.325357 | 9.41333 |  |  |
| 635 | 0.0662676 | 1.12472 | 1.22322 | 0.207953 | 5.88222 |  |  |
| 636 | 0.0014406 | 0.0566022 | 0.308397 | 0.0595339 | 5.18019 |  |  |
| 637 | 0.0062426 | 0.22727 | 0.567294 | 0.0986687 | 5.74948 |  |  |
| 638 | 0.0220892 | 0.776793 | 0.977136 | 0.108452 | 9.00982 |  |  |
| 639 | 0.012005 | 0.42764 | 0.724479 | 0.088885 | 8.15075 |  |  |
| 640 | 0.004802 | 0.151663 | 0.401192 | 0.128851 | 3.11361 |  |  |
| 641 | 0.0067228 | 0.219565 | 0.520028 | 0.119068 | 4.36749 |  |  |
| 642 | 0.0340942 | 0.807392 | 1.00866 | 0.187554 | 5.378 |  |  |
| 643 | 0.0172872 | 0.50712 | 1.28413 | 0.176938 | 7.25754 |  |  |
| 644 | 0.0052822 | 0.167518 | 0.439976 | 0.128851 | 3.4146 |  |  |
| 645 | 0.016807 | 0.536507 | 1.16571 | 0.128852 | 9.04695 |  |  |
| 646 | 0.0028812 | 0.0984465 | 0.304721 | 0.119068 | 2.55921 |  |  |
| 647 | 0.007203 | 0.220149 | 0.491903 | 0.128852 | 3.81759 |  |  |
| 648 | 0.0009604 | 0.0381041 | 0.208249 | 0.0925057 | 2.2512 |  |  |
| 649 | 0.0004802 | 0.019606 | 0.0595339 | 0.0595339 | 1 |  |  |
| 650 | 0.0014406 | 0.0560173 | 0.275414 | 0.0693176 | 3.97323 |  |  |
| 651 | 0.0004802 | 0.019606 | 0.0595339 | 0.0595339 | 1 |  |  |
| 652 | 0.0057624 | 0.210464 | 0.517091 | 0.088885 | 5.81752 |  |  |
| 653 | 0.0014406 | 0.0560173 | 0.279906 | 0.0693181 | 4.03799 |  |  |
| 654 | 0.0009604 | 0.0381041 | 0.213139 | 0.0595339 | 3.58013 |  |  |
| 655 | 0.0052822 | 0.194829 | 0.628523 | 0.088885 | 7.07119 |  |  |
| 656 | 0.0028812 | 0.110342 | 0.480114 | 0.088885 | 5.40152 |  |  |
| 657 | 0.0014406 | 0.0566022 | 0.308397 | 0.0595339 | 5.18019 |  |  |
| Mean | 4.81886 | 8.14316 | 1.28764 | 0.321068 | 3.88878 |  |  |
|  |  |  |  |  |  |  |  |
|  |  |  |  |  |  |  |  |
